# Association of Intraoperative Opioid Administration With Postoperative Pain and Opioid Use

**DOI:** 10.1001/jamasurg.2023.2009

**Published:** 2023-06-14

**Authors:** Laura A. Santa Cruz Mercado, Ran Liu, Kishore M. Bharadwaj, Jasmine J. Johnson, Rodrigo Gutierrez, Proloy Das, Gustavo Balanza, Hao Deng, Akriti Pandit, Tom A. D. Stone, Teresa Macdonald, Caroline Horgan, Si Long (Jenny) Tou, Timothy T. Houle, Edward A. Bittner, Patrick L. Purdon

**Affiliations:** 1 Department of Anesthesia, Critical Care, and Pain Medicine, Massachusetts General Hospital, Boston; 2 Department of Anesthesia, Harvard Medical School, Boston, Massachusetts; 3 Department of Anesthesia, Critical Care, and Pain Medicine, Beth Israel Deaconess Medical Center, Boston, Massachusetts; 4 Center for Perioperative Care, Department of Surgery, Massachusetts General Hospital, Boston

## Abstract

**Importance:**

Opioids administered to treat postsurgical pain are a major contributor to the opioid crisis, leading to chronic use in a considerable proportion of patients. Initiatives promoting opioid-free or opioid-sparing modalities of perioperative pain management have led to reduced opioid administration in the operating room, but this reduction could have unforeseen detrimental effects in terms of postoperative pain outcomes, as the relationship between intraoperative opioid usage and later opioid requirements is not well understood.

**Objective:**

To characterize the association between intraoperative opioid usage and postoperative pain and opioid requirements.

**Design, Setting, and Participants:**

This retrospective cohort study evaluated electronic health record data from a quaternary care academic medical center (Massachusetts General Hospital) for adult patients who underwent noncardiac surgery with general anesthesia from April 2016 to March 2020. Patients who underwent cesarean surgery, received regional anesthesia, received opioids other than fentanyl or hydromorphone, were admitted to the intensive care unit, or who died intraoperatively were excluded. Statistical models were fitted on the propensity weighted data set to characterize the effect of intraoperative opioid exposures on primary and secondary outcomes. Data were analyzed from December 2021 to October 2022.

**Exposures:**

Intraoperative fentanyl and intraoperative hydromorphone average effect site concentration estimated using pharmacokinetic/pharmacodynamic models.

**Main Outcomes and Measures:**

The primary study outcomes were the maximal pain score during the postanesthesia care unit (PACU) stay and the cumulative opioid dose, quantified in morphine milligram equivalents (MME), administered during the PACU stay. Medium- and long-term outcomes associated with pain and opioid dependence were also evaluated.

**Results:**

The study cohort included a total of 61 249 individuals undergoing surgery (mean [SD] age, 55.44 [17.08] years; 32 778 [53.5%] female). Increased intraoperative fentanyl and intraoperative hydromorphone were both associated with reduced maximum pain scores in the PACU. Both exposures were also associated with a reduced probability and reduced total dosage of opioid administration in the PACU. In particular, increased fentanyl administration was associated with lower frequency of uncontrolled pain; a decrease in new chronic pain diagnoses reported at 3 months; fewer opioid prescriptions at 30, 90, and 180 days; and decreased new persistent opioid use, without significant increases in adverse effects.

**Conclusions and Relevance:**

Contrary to prevailing trends, reduced opioid administration during surgery may have the unintended outcome of increasing postoperative pain and opioid consumption. Conversely, improvements in long-term outcomes might be achieved by optimizing opioid administration during surgery.

## Key Points

**Question:**

What is the association between intraoperative opioid administration and postoperative pain and opioid use?

**Findings:**

In this cohort study of 61 249 individuals undergoing surgery, greater intraoperative fentanyl and hydromorphone administration was associated with decreased pain and opioid administration in postanesthesia care units. In particular, greater fentanyl administration was associated with decreased new chronic pain diagnoses at 3 months, decreased opioid prescriptions at 30, 90, and 180 days, and decreased new persistent opioid use, without significant increases in adverse effects.

**Meaning:**

The results indicate that intraoperative opioid administration was associated with short- and long-term postoperative pain and opioid outcomes; reduced opioid administration during surgery may have the unintended outcome of increasing postoperative pain and opioid use.

## Teaser

This cohort study evaluates associations between intraoperative opioid use and postoperative pain and opioid use.

## Introduction

The opioid crisis is a serious public health issue in the US.^1,2^ About 1.7 million people experienced substance use disorders related to prescription opioid use in 2017.^3^ In 2021, drug overdoses caused more than 100 000 deaths in the US; opioids were responsible for more than 75 000 of these deaths.^4^ Postsurgical pain management is a major contributor to the crisis.^5^ Of the 51 million patients who undergo surgery each year in the US, between 9% and 13% continue long-term use of opioids.^6,7^ Moreover, a considerable proportion of surgical patients experience persistent postsurgical pain^8^ and chronic pain,^9^ and 8% to 12% of these individuals develop opioid use disorder.^10^ As a result, initiatives began promoting opioid-free or opioid-sparing modalities of perioperative pain management.^11,12,13^

In anesthesiology, the drive toward opioid reduction in surgical pain management has led to reduced intraoperative opioid usage during general anesthesia.^14^ However, the effects of reduced intraoperative opioid usage on postoperative pain outcomes have not been well studied.^15^ Some investigators have argued that this reduction may have unknown outcomes^16^ and may prove detrimental for postoperative pain management.^17^ Some studies indicate that intraoperative opioid administration can effect lasting reductions in postoperative pain, and thus reduce net opioid usage.^18,19^ Conversely, inadequate perioperative pain relief has been associated with adverse patient outcomes. Uncontrolled pain can hinder postsurgical recovery, increasing mortality, duration of stay, and the risk of chronic pain, which in turn increases the likelihood of chronic opioid usage.^8,20,21^

This study seeks to understand the association between intraoperative opioid administration and postoperative pain and opioid usage over the short and long term. We conducted a retrospective analysis of electronic health record data from a large cohort of surgical cases, using propensity weighting^22^ to adjust for confounders on intraoperative opioid exposure.

## Methods

### Design, Data Source, and Study Population

The study design and a waiver of informed consent were approved by the Massachusetts General Hospital institutional review board (2020P000301). We established an a priori statistical analysis plan that defined all variables, exposures, and outcomes, and outlined analysis procedures (eMethods in Supplement 1).

The study included patients who underwent noncardiac surgery with general anesthesia at a quaternary academic medical center (Massachusetts General Hospital, Boston, Massachusetts) between April 1, 2016, and March 31, 2020. We used data from patient electronic health records and conducted a complete case analysis. Race and ethnicity were included as patient baseline variables, and their association with intraoperative exposure and clinician decision making were controlled for in our propensity weighting. These variables were also reported to provide an accurate account of the demographic characteristics of our study cohort. Categories were determined based on how these variables were recorded in the electronic health record (Epic) and on National Institutes of Health notice NOT-OD-15-089 (https://grants.nih.gov/grants/guide/notice-files/not-od-15-089.html). We included patients 18 years and older who were admitted to the postanesthesia care unit (PACU) after surgery. We excluded patients who underwent cesarean surgery, received regional anesthesia as a complement to general anesthesia, received opioids other than fentanyl or hydromorphone during the exposure window, received postoperative patient-controlled analgesia, were admitted to the intensive care unit, or who died during the intraoperative period. Data were analyzed from December 2021 to October 2022.

### Exposures

The exposures we studied were the mean intraoperative effect site concentrations for fentanyl and hydromorphone. Effect site concentrations were computed using previously validated pharmacokinetic/pharmacodynamic models^23,24^ parameterized by patient demographic characteristics and records of opioid administration (eMethods in Supplement 1).

### Outcomes

The primary study outcomes were maximal pain score during the PACU stay and cumulative opioid dose administered in the PACU, quantified in morphine milligram equivalents (MME). Secondary outcomes included frequency of uncontrolled pain at 24 hours; new instances of chronic pain diagnosis between 3 months and 1 year; total opioid use at 24 hours and in the hospital; opioid prescriptions at 30, 90, and 180 postoperative days; frequency of new persistent opioid use at 90 and 180 days; maximal pain score in the first 24 hours and in the hospital; incidence of opioid-related complications in the PACU (ie, postoperative nausea and vomiting, sedation, and respiratory depression), length of stay in the PACU and in the hospital; 30-day readmission; and 30-day mortality (eMethods in Supplement 1).

### Covariates

We included additional adjustment variables as covariates in each model to account for potential confounds occurring concurrently with or after the exposure (eTable 1 in Supplement 1). These variables increase modeling precision but are not interpreted, as they are not controlled for confounding.

### Statistical Analysis

We calculated a multivariate propensity score^25^ for each patient using the specified confounding variables (eMethods in Supplement 1). This weighting controls for the effects of the confounding variables on the exposures. We calculated the correlation with exposures before and after reweighting using Pearson correlation for continuous and binary variables and the Kendall rank correlation^26^ for ordinal variables. Outlier propensity weights above the 99.5th percentile were discarded.

We rescaled continuous variables to a mean of 0 and standard deviation of 1. We binarized categorical variables by one-hot encoding, which represents each category by an indicator variable. We used ordinal regression to model pain score outcomes (ie, PACU maximum pain score, 24-hour postoperative maximum pain score, and in-hospital maximum pain score). We used hurdle models consisting of a binomial logistic regression and a log-normal component for opioid usage outcomes (ie, PACU total MME, 24-hour postoperative total MME, and in-hospital total MME). We used Cox proportional hazard models to model time-to-event outcomes (ie, recovery from sedation, PACU length of stay, and hospital length of stay). We fitted logistic regression models for each binary outcome (ie, frequency of uncontrolled pain; 3-month and 12-month new chronic pain diagnoses; 30-day, 90-day, and 180-day opioid prescription; new persistent opioid use; postoperative nausea and vomiting in the PACU; respiratory depression; 30-day postoperative mortality; and 30-day postoperative readmission).

We conducted sensitivity analyses with control individuals for confounding of exposures by comorbidities and surgical complexity as represented by *International Statistical Classification of Diseases and Related Health Problems, Tenth Revision* (*ICD-10*) and *Current Procedural Terminology* (*CPT*) codes through propensity weighting on the impact of interaction terms in models of primary and secondary outcomes (eFigures 1-3 in Supplement 1) and in the subgroup of patients with body mass index (calculated as weight in kilograms divided by height in meters squared) greater than 30 (eFigures 4-5 in Supplement 1).

We made counterfactual predictions of the mean expected difference in each outcome resulting from the additional administration of intraoperative fentanyl, 100 µg, or intraoperative hydromorphone, 500 µg, using fitted models, computed on the unweighted study population (eMethods in Supplement 1). For predictions of expected event times using Cox proportional hazard models,^27^ we assumed a constant baseline hazard rate. We computed confidence intervals by bootstrap. Statistical analyses were performed using R version 4.2.2 (R Foundation).

## Results

**Figure 1.**
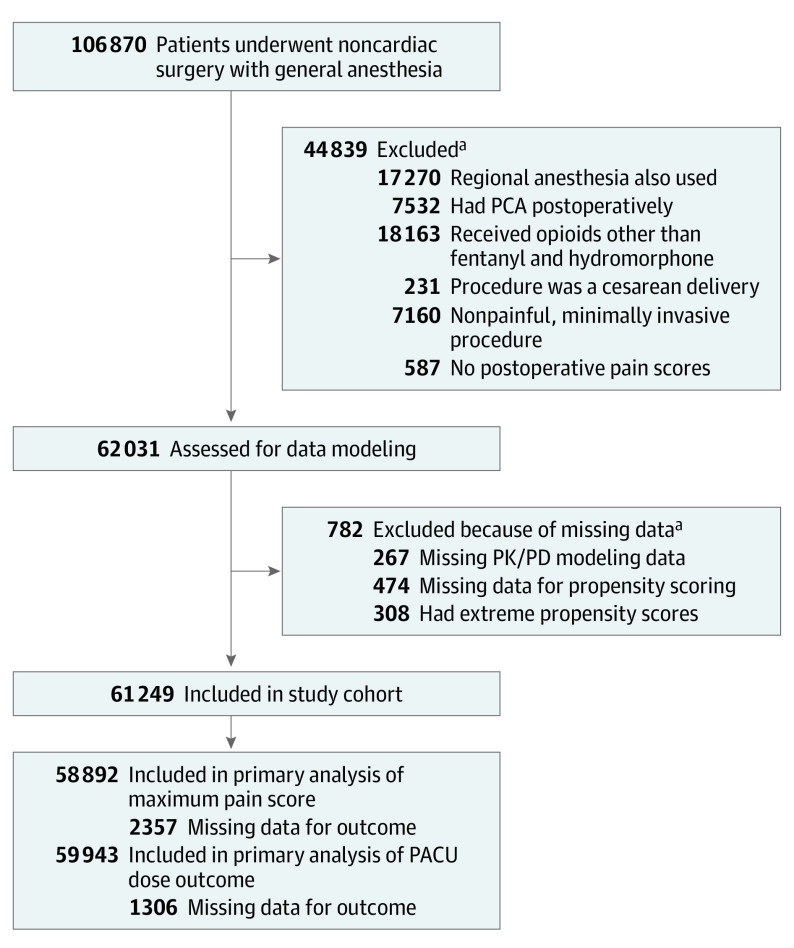
Flow Diagram of Study Population PACU indicates postanesthesia care unit; PCA, patient-controlled analgesia; PK/PD, pharmacokinetic/pharmacodynamic. ^a^Some individuals met multiple exclusion criteria.

The study cohort included 61 249 individuals (mean [SD] age, 55.44 [17.08] years; 32 778 [53.5%] female) (Figure 1). A total of 2084 individuals (3.4%) were Asian, 2972 (4.9%) were Black, 273 (0.4%) were Hispanic, 50 093 (81.8%) were White, and 5827 (9.5%) were of another race or ethnicity (including American Indian or Alaska Native, Native Hawaiian or Other Pacific Islander, or recorded values that did not fall into any of the National Institutes of Health Office of Management and Budget–defined categories, such as unavailable or multiple; these categories were consolidated as their numbers were too few for accurate statistical inference or had values that were not informative) (Table 1). Orthopedic, general, urological, gynecological, and plastic were the most frequent operations, and together made up 72.3% of all included procedures. A total of 1957 unique *CPT* codes were associated with these procedures; the 25 most frequent made up 37.2% of operations (eTable 2 in Supplement 1). Propensity weighting reduced correlations between exposures and confounding variables (eMethods in Supplement 1).

**Table 1.** Baseline Statistics and Clinical Characteristics With Propensity-Weighted Correlations of Study Population

Statistic	No. (%)	Correlation with fentanyl (unweighted/weighted)	Correlation with hydromorphone (unweighted/weighted)
Total No. of patients	61 249 (100)	NA	NA
Demographic characteristics			
Age, mean (SD) y	55.44 (17.08)	−0.06/−0.02	−0.12/−0.08
Sex			
Male	28 471 (46.48)	−0.09/−0.04	−0.02/−0.01
Female	32 778 (53.52)		
Race and ethnicity			
Asian	2084 (3.4)	0.04/−0.01	0.00/0.01
Black	2972 (4.9)	−0.01/−0.01	0.00/0.05
Hispanic	273 (0.4)	0.00/−0.01	−0.01/−0.02
White	50 093 (81.8)	−0.02/0.01	−0.00/−0.05
Other^b^	5827 (9.5)	0.01/0.01	0.01/0.03
Clinical characteristics			
ASA physical status classification			
I	6951 (11.35)	−0.06/−0.00	−0.05/−0.00
II	36 013 (58.80)		
III	17 405 (28.42)		
IV	880 (1.44)		
BMI^c^	27.26 (23.78 to 31.63)	−0.25/−0.05	−0.09/−0.10
Ambulatory surgery	29 165 (47.62)		
Inpatient surgery	32 084 (52.38)	−0.03/−0.03	0.17/0.13
Surgical service			
Orthopedic surgery	14 033 (22.91)	−0.05/−0.04	0.12/0.11
General surgery	10 539 (17.21)	0.03/0.03	−0.06/−0.03
Urology	8596 (14.03)	−0.06/−0.01	−0.09/−0.08
Gynecology	6177 (10.09)	−0.00/0.05	−0.02/−0.03
Plastic surgery	4920 (8.03)	0.01/−0.00	0.05/0.04
Other	16 984 (27.73)	0.06/−0.01	−0.01/−0.03
Procedural severity score			
Morbidity	33.45 (19.02)	0.03/−0.02	−0.02/0.01
Mortality	53.40 (34.68)	0.05/−0.00	−0.04/−0.00
30-d Mortality	256 (0.42)	0.00/0.01	−0.01/−0.02
30-d Readmission	4407 (7.20)	−0.01/−0.03	0.05/0.05
Surgical duration, median, IQR, h	1.15 (0.63 to 1.98)	−0.01/−0.03	0.02/0.05
PACU length of stay, median, IQR, h	1.52 (1.05 to 2.17)	−0.01/−0.03	0.02/0.05
Inpatient hospital length of stay, median, IQR, h	56.14 (30.95 to 130.25)	0.00/−0.05	0.16/0.16
Elixhauser comorbidity index, median, IQR	1.00 (0.0 to 7.0)	0.00/−0.08	−0.04/−0.03
Opioid naivety	49 428 (80.70)	0.02/−0.01	−0.04/−0.02
Intraoperative characteristics			
Mean blood pressure, mm Hg	81.53 (9.44)	−0.04/0.07	0.01/−0.01
Heart rate, bpm	71.50 (11.65)	0.05/0.04	0.11/0.12
Hypertensive duration, median, IQR, min	5.00 (0.00 to 17.00)	−0.03/−0.00	0.06/0.09
Hypotensive duration, median, IQR, min	44.00 (18.00 to 83.00)	−0.14/−0.13	0.08/0.06
Vasopressors, median, IQR, µg/kg/min norepinephrine equivalents	0.00 (0.00 to 0.01)	−0.01/−0.02	−0.01/−0.02
Blood loss, median, IQR, mL	2.00 (0.00 to 50.00)	−0.05/−0.04	−0.01/−0.02
Blood transfusion	1852 (3.02)	−0.01/−0.04	0.07/0.07
Anesthesia			
N2O	43 245 (70.61)	−0.01/−0.04	0.01/0.05
Desflurane	733 (1.20)	−0.22/−0.23	−0.07/−0.15
Isoflurane	4628 (7.56)	−0.02/−0.03	0.04/0.08
Propofol	59 188 (96.64)	−0.01/−0.01	0.00/0.03
Sevoflurane	54 025 (88.21)	−0.06/−0.06	0.10/0.09
Neuromuscular blocks	20 086 (32.79)	−0.11/−0.05	0.08/0.05
Antiemetic prophylaxis	12 349 (20.16)	−0.02/−0.05	0.15/0.13
Nonopioid analgesia			
Ketorolac	15 685 (25.61)	0.03/0.03	−0.03/−0.05
Acetaminophen	2275 (3.71)	−0.03/−0.03	0.00/−0.03
Ketamine	3367 (5.50)	−0.06/−0.06	0.08/0.05
Dexmedetomidine	1395 (2.28)	−0.05/−0.04	−0.01/−0.04
Esmolol	7071 (11.54)	−0.01/−0.01	0.00/0.03
Lidocaine	49 888 (81.45)	−0.03/−0.03	0.01/−0.01
Postoperative complications			
Respiratory depression	941 (1.54)	−0.00/0.00	0.01/0.02
PONV in PACU	1642 (2.68)	0.00/0.01	0.01/0.03
Difficult intubation	257 (0.42)	−0.01/−0.01	−0.01/−0.02

Abbreviations: ASA, American Society of Anesthesiologists; BMI, body mass index; NA, not applicable; PACU, postanesthesia care unit; PONV, postoperative nausea and vomiting.

^a^ Race and ethnicity were included as patient baseline variables, and their association with intraoperative exposure and clinician decision-making were controlled for in our propensity weighting. These variables were also reported to provide an accurate account of the demographic characteristics of our study cohort. Categories were determined based on how these variables were recorded in the electronic health record (Epic) and on National Institutes of Health notice NOT-OD-15-089 (https://grants.nih.gov/grants/guide/notice-files/not-od-15-089.html).

^b^ Including American Indian or Alaska Native, Native Hawaiian or Other Pacific Islander, or recorded values that did not fall into any of the National Institutes of Health Office of Management and Budget–defined categories, such as unavailable or multiple; these categories were consolidated as their numbers were too few for accurate statistical inference or had values that were not informative.

^c^ Calculated as weight in kilograms divided by height in meters squared.

**Figure 2.**
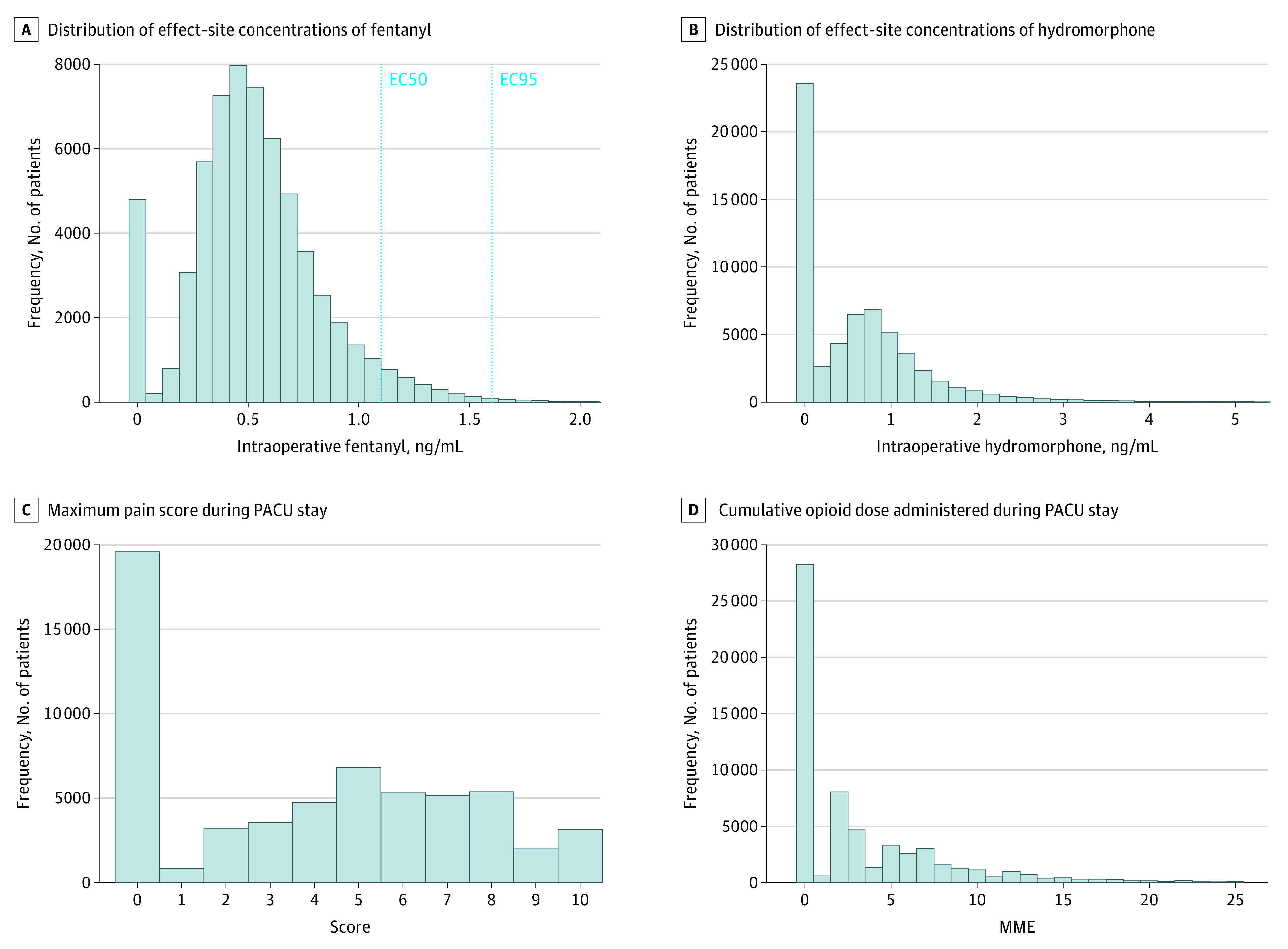
Population Distribution of Exposures and Primary Outcomes A, Vertical dashed lines represent the EC50 and EC95 for intraoperative fentanyl based on Vuyk et al.^28^ MME indicates morphine milligram equivalent; PACU, postanesthesia care unit.

Both intraoperative exposure variables are bimodally distributed (Figure 2A and B),^28^ with 1 peak at 0. The median (IQR) exposures were 0.50 (0.35-0.68) ng/mL of fentanyl and 0.50 (0.00-0.96) ng/mL of hydromorphone, and the 95th percentile exposures were 1.07 ng/mL of fentanyl and 2.04 ng/mL of hydromorphone. The 2 exposure variables were approximately independent, with a Pearson correlation of −0.03. During our defined exposure window, 4501 patients (7.3%) received no fentanyl, and 22 299 patients (36.4%) received no hydromorphone. Fentanyl administration tended to occur toward the beginning of the exposure window, while hydromorphone administration tended to be homogenously distributed along the exposure window (eFigure 6 in Supplement 1). PACU maximum pain score (Figure 2C) and PACU total MME (Figure 2D) also followed a bimodal distribution, with a maximum pain score of 0 in the PACU and no opioids given in the PACU as modal values. Maximum pain score in the 24 postoperative hours and in the hospital were distributed similarly to PACU maximum pain (eFigure 7 in Supplement 1). The median (IQR) number of pain assessments per patient was 3 (4-6) in the PACU, 14 (11-18) in the 24 hours following surgery, and 23 (14-43) in the hospital (eFigure 8 in Supplement 1). We also examined mean opioid administration by year at Massachusetts General Hospital, which showed a decrease over time (eFigure 9 in Supplement 1), and the total dosage of fentanyl and hydromorphone administered between patient arrival in the operating room and the end of the exposure window (eFigure 10 in Supplement 1).

### Modeling of Primary and Secondary Outcomes

**Figure 3.**
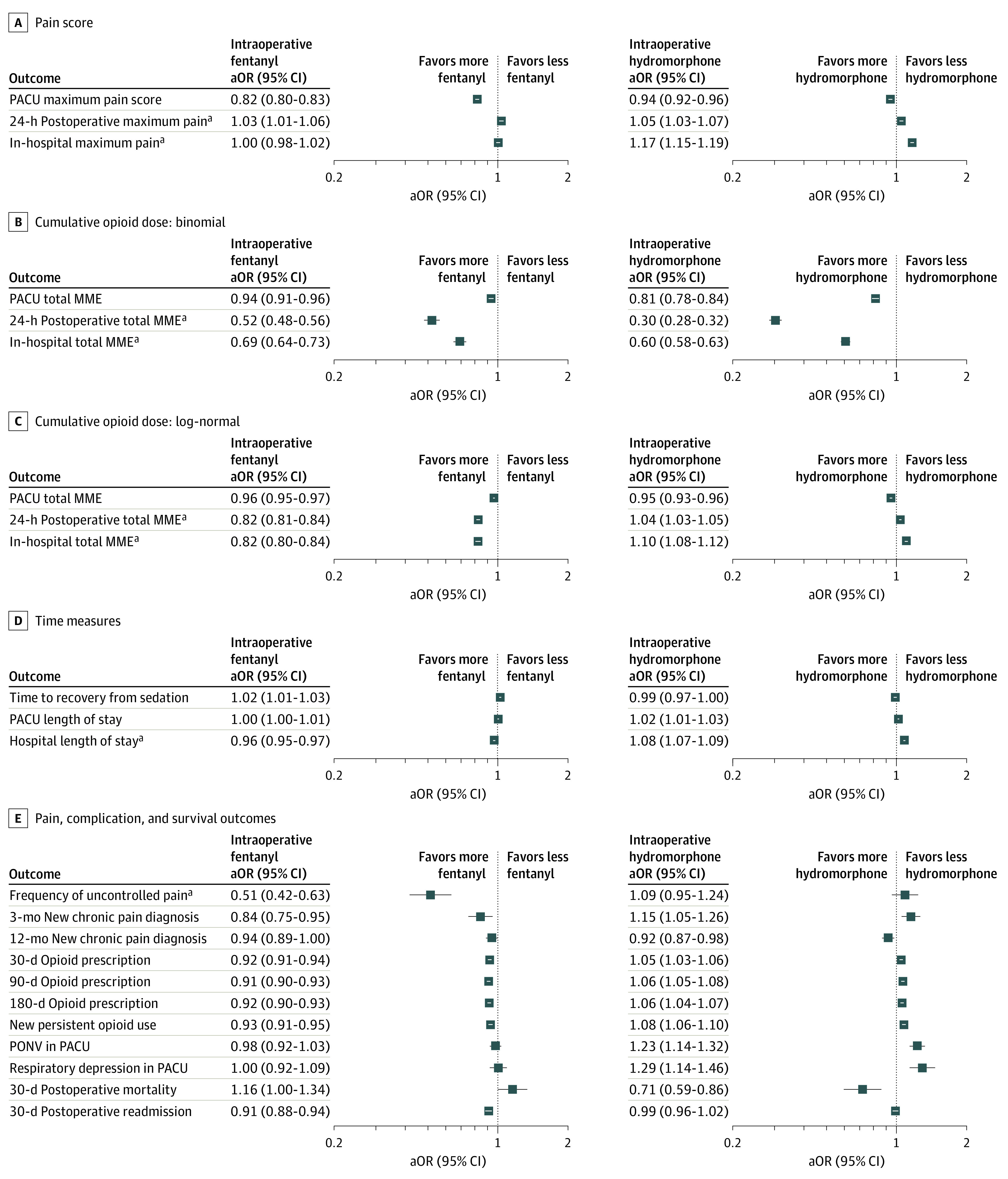
Adjusted Odds Ratios (aOR) for Primary and Secondary Outcomes The forest plot represents adjusted odds ratios with confidence intervals for each outcome for intraoperative fentanyl and hydromorphone exposure. We calculated odds ratios by exponentiating model coefficients. For hierarchical models, both components are presented (binomial and log-normal). For Cox proportional hazards models, adjusted odds ratios for expected time to event are presented. MME indicates morphine milligram equivalent; PACU, postanesthesia care unit; PONV, postoperative nausea and vomiting. ^a^Secondary outcomes that were evaluated only in the subset of inpatients. ^b^We computed hazard ratios by exponentiating coefficients of Cox proportional hazards models, which are ratios of rates of event occurrence to population baseline rates. Greater hazard rates predict shorter event occurrence times; for example, for a 1-SD increase in intraoperative fentanyl exposure, our model predicts a hazard ratio with a 95% CI from 1.04 to 1.10, indicating an increased rate of hospital discharge, and a shorter length of stay.

We fit statistical models on the propensity-weighted data set to describe the effect of intraoperative opioid exposures on primary and secondary outcomes (Figure 3). Intraoperative fentanyl and intraoperative hydromorphone were both associated with lower maximum pain scores in the PACU. Both exposures were also associated with a lower probability of opioid administration in the PACU, indicated by a negative coefficient in the binomial component of the PACU total MME hurdle model, and lower total opioid dosage in the PACU, indicated by a negative coefficient in the log-normal component.

Hydromorphone and fentanyl had different patterns of model-predicted associations with some medium- to long-term outcomes. Intraoperative fentanyl was associated with reduced total opioid administration over the postoperative 24-hour period and with reduced total in-hospital opioid administration. Intraoperative hydromorphone administration was also associated with reduced total opioid administration over the postoperative 24-hour period and with a lower probability of opioid administration in the hospital, but was associated with greater opioid administration in the hospital conditioned on nonzero opioid administration. Intraoperative fentanyl exposure was associated with a shorter hospital length of stay, whereas intraoperative hydromorphone was associated with a longer hospital length of stay. Intraoperative fentanyl was associated with reduced frequency of uncontrolled pain as well as reduced opioid prescriptions at 30, 90, and 180 days, whereas intraoperative hydromorphone was associated with a slight increase in 30-, 90-, and 180-day opioid prescriptions.

### Counterfactual Predictions

We computed the expected association of the addition of fentanyl, 100 µg, or hydromorphone, 500 µg, with the observed intraoperative exposure of each patient in our study population (Table 2). These analyses indicated that a 100-µg increase in intraoperative fentanyl would correspond to a 0.49-point mean reduction in maximum pain score in the PACU and a 500-µg increase in intraoperative hydromorphone would correspond to a 0.08-point mean reduction in maximum pain score in the PACU.

**Table 2.** Counterfactual Predictions

Change in outcome	Expected change (95% CI)	
	With additional 100 µg of fentanyl	With additional 500 µg of hydromorphone
Primary PACU maximum pain score	−0.49 (−0.53 to −0.44)	−0.08 (−0.11 to −0.06)
24-h Postoperative maximum pain	0.09 (0.03 to 0.15)	0.07 (0.04 to 0.10)
In-hospital maximum pain	0.01 (−0.05 to 0.06)	0.24 (0.21 to 0.27)
Primary PACU total MME^a^	−0.36 (−0.43 to −0.29); −12.7 (−15.3 to −10.2)	−0.41 (−0.46 to −0.35); −14.4 (−16.3 to −12.5)
24-h Postoperative total MME^a^	−4.9 (−5.2 to −4.6); −45.6 (−48.2 to −42.8)	−1.5 (−1.6 to −1.3); −13.4 (−14.8 to −12.0)
In-hospital total MME^a^	−16.6 (−18.6 to −14.9); −38.6 (−42.9 to −34.4)	3.5 (2.6 to 4.6); 8.2 (6.0 to 10.7)
Time to recovery from sedation, min^a^	1.80 (1.47 to 3.72); 5.1 (4.2 to 10.6)	−0.57 (−1.70 to 0.11); −1.6 (−4.9 to 0.3)
PACU length of stay, min^a^	0.48 (−0.96 to 2.34); 0.7 (−1.4 to 3.4)	1.54 (1.30 to 3.17); 2.2 (1.9 to 4.6)
Hospital length of stay, min^a^	−444.8 (−569.9 to −317.5); −11.1 (−14.3 to −8.0)	563.9 (493.7 to 632.0); 14.1 (12.4 to 15.8)
Frequency of uncontrolled pain (No. per 1000)^a^	−4.71 (−6.46 to −4.16); −56.3 (−77.3 to −49.8)	0.59 (−0.33 to 1.76); 7.1 (−4.0 to 21.0)
3-mo New chronic pain diagnosis (No. per 1000)^a^	−1.34 (−2.25 to −0.46); −30.3 (−50.9 to −10.3)	0.80 (0.33 to 1.50); 18.0 (7.4 to 33.9)
12-mo New chronic pain diagnosis (No. per 1000)^a^	−2.28 (−4.31 to −0.06); −11.9 (−22.6 to −0.3)	−1.79 (−2.94 to −0.62); −9.3 (−15.4 to −3.3)
30-d Opioid prescription (No. per 1000)^a^	−31.16 (−37.54 to −24.69); −12.2 (−14.7 to −9.7)	10.29 (6.39 to 14.11); 4.0 (2.5 to 5.5)
90-d Opioid prescription (No. per 1000)^a^	−36.16 (−42.74 to −29.32); −13.3 (−15.7 to −10.8)	14.60 (10.68 to 18.58); 5.4 (3.9 to 6.8)
180-d Opioid prescription (No. per 1000)^a^	−35.04 (−41.72 to −28.70); −12.3 (−14.7 to −10.1)	13.08 (9.16 to 17.05); 4.6 (3.2 to 6.0)
New persistent opioid use (No. per 1000)^a^	−21.29 (−26.98 to −15.74); −12.4 (−15.7 to −9.2)	12.74 (9.45 to 16.07); 7.4 (5.5 to 9.3)
PONV in PACU (No. per 1000)^a^	−1.41 (−4.56 to 2.06); −4.8 (−15.6 to 7.0)	7.78 (4.73 to 11.07); 26.6 (16.2 to 37.8)
Respiratory depression in PACU (No. per 1000)^a^	0.13 (−2.08 to 2.86); 0.9 (−14.5 to 19.9)	4.97 (2.44 to 8.16); 34.7 (17.0 to 56.9)
30-d Postoperative mortality (No. per 1000)^a^	0.93 (−0.029 to 2.34); 29.4 (−0.9 to 73.9)	−0.89 (−1.47 to −0.45); −28.2 (−46.6 to −14.3)
30-d Postoperative readmission (No. per 1000)^a^	−10.48 (−13.94 to −6.77); −16.2 (−21.5 to −10.4)	−0.75 (−2.54 to 1.20); −1.2 (−3.9 to 1.8)

Abbreviations: MME, morphine milligram equivalents; PACU, postanesthesia care unit; PONV, postoperative nausea and vomiting.

^a^ Also reported as percentages with 95% CIs.

For postoperative opioid administration, we found that a 100-µg increase in intraoperative fentanyl would correspond to mean reductions of 0.36 MME postoperative opioid administration in the PACU (−12.7%), 4.9 MME at 24 hours (−45.6%), and 16.6 MME in the hospital (−38.6%). Meanwhile, a 500-µg increase in intraoperative hydromorphone would correspond to mean reductions of 0.41 MME of opioid administration in the PACU (−14.4%) and 1.5 MME at 24 hours (−17.7%) and an increase of 3.5 MME in the hospital (+8.2%). We also found that a 100-µg increase in intraoperative fentanyl would correspond to a 5.1-hour reduction in hospital length of stay (−7.7%), whereas a 500-µg increase in intraoperative hydromorphone would correspond to a 6.5-hour increase in hospital length of stay (+9.8%).

For opioid prescriptions, we found that a 100-µg increase in intraoperative fentanyl would correspond to decreases of 31.2 instances per 1000 after 30 days (−12.2%), 36.2 instances per 1000 after 90 days (−13.3%), and 35.0 instances per 1000 after 180 days (−12.3%), alongside a decrease of 21.3 instances per 1000 of new persistent opioid use (−12.4%). A 500-µg increase in intraoperative hydromorphone would correspond to increases of 10.3 opioid prescriptions per 1000 after 30 days (+4.0%), 14.6 opioid prescriptions per 1000 after 90 days (+5.4%), and 13.1 opioid prescriptions per 1000 after 180 days (+4.6%), alongside an increase of 12.7 instances of new persistent opioid use per 1000 (+7.4%).

## Discussion

### Clinical Significance

In this cohort study, we sought to understand the associations between intraoperative opioid administration and both short- and long-term outcomes with respect to postoperative pain and opioid administration through statistical modeling of a large study cohort representative of a broad general population of surgical patients. Our primary analyses indicate that increased intraoperative administration of both fentanyl and hydromorphone was associated with reduced postoperative pain in the PACU and lower quantities of opioids administered in the PACU. Our results also suggest that intraoperative mitigation of surgical nociception may have lasting effects on long-term pain and subsequent opioid use. Therefore, it may be necessary to reevaluate opioid-sparing anesthetic regimens since reductions in intraoperative opioid usage may have the unintended effect of worsening long-term pain and increasing overall opioid use.

Intraoperative opioid administration may also impact health care costs and efficiency. We found that higher fentanyl administration was associated with reduced length of stay and 30-day readmissions without an increase in adverse effects. Other the other hand, increased hydromorphone administration was associated with increased length of stay, respiratory depression, and postoperative nausea and vomiting.

We were surprised by the extent to which intraoperative administration of opioids was associated with medium- and long-term outcomes. This may relate to the fact that if inadequate amounts of opioids are administered in the operating room, patients may emerge from general anesthesia in pain, a phenomenon that has a known association with persistent postsurgical pain.^8,9,29,30^

Fentanyl and hydromorphone appear to have had different associations with some medium- and long-term outcomes. This difference may involve the timing of drug administration. In our cohort, fentanyl tended to be administered earlier than hydromorphone. Furthermore, fentanyl has a much shorter duration of effect than hydromorphone^31,32,33^ so its activity is largely confined to the intraoperative period, whereas hydromorphone continues acting into the postoperative period. This may indicate that the moment-to-moment control of nociception during surgery could influence medium- and long-term outcomes more strongly than long-acting opioids administered near the end of a procedure with the intent of controlling postoperative pain. Ultimately, randomized clinical trials assessing the relationship between the timing of opioid administration and pain-related outcomes are necessary, as our models quantify exposure-outcome associations but cannot conclusively identify the mechanisms responsible.

### Counterfactual Predictions

Our counterfactual predictions contextualize the magnitude of effects stemming from typical clinically relevant incremental doses of fentanyl and hydromorphone.^34,35^ The predicted percentage reduction in hospital-administered MME, opioid prescriptions through 180 days, and new persistent opioid use after an incremental 100-µg dose of fentanyl appear comparable to the outcomes of major interventions in opioid prescribing practices.^36,37,38,39^ This same dose of fentanyl also predicted a significant reduction in length of stay. While opioid prescribing practices shift toward limitation, our counterfactual predictions would seem to reflect a reduction in the intrinsic need for opioids after surgery attained by improved control of intraoperative nociception. Our counterfactual analysis also predicted that incremental amounts of hydromorphone might increase opioid prescriptions through 180 days and new persistent opioid use, which may reflect differences in drug mechanisms and timing.

### Pharmacokinetic/Pharmacodynamic Modeling

The mean effect site concentration of fentanyl and hydromorphone during the exposure window was computed using pharmacokinetic/pharmacodynamic modeling. While pharmacokinetic/pharmacodynamic models cannot represent a drug’s behavior at the individual level, these models provide a sound theoretical basis for estimating opioid concentrations on average for a population of patients, accounting for variables such as weight, height, and age.^40^ Effect site concentrations place current patterns of opioid administration in the context of the known pharmacodynamics of these drugs: in this cohort, fentanyl is generally administered well below its EC50.^28^ Furthermore, we found that fentanyl administration in this cohort decreased between 2016 and 2020 (eFigure 9 in Supplement 1). This trend toward decreasing intraoperative opioid administration has been well documented at many other institutions in the US.^14^

### Limitations

This study has limitations. Because the study population is derived from patients at a single medical center, our findings reflect its patterns of practice. While our models predict overall improvements in outcome with increased intraoperative opioid administration, increased intraoperative opioid administration may prove detrimental in a clinical setting where typical care involves a higher level of intraoperative opioid administration. The generalizability of our study may also be limited by the relatively low proportion of patients from various racial and ethnicity groups.

While propensity weighting produced low correlations between exposures and confounders, some residual confounding inevitably remains. Our finding that greater hydromorphone exposure was associated with on some detrimental long-term outcomes may be influenced by residual confounding; that is, patients undergoing more painful operations may have received more hydromorphone even after rebalancing. Our sensitivity analysis (eFigure 1 in Supplement 1) showed that altering the set of controlled confounding variables to account for comorbidities and surgical complexity influenced the magnitude of the modeled effects of the intraoperative exposures but did not change their direction or subjective interpretation.

## Conclusions

The main implication of this study is that in the drive toward overall reduction of opioid usage in surgical pain management in the US, the role of intraoperative nociception in determining postoperative pain may have been overlooked to the detriment of patient outcomes. Adequate intraoperative nociception management, particularly with fentanyl, may result in improvements in postoperative pain management with a lower total quantity of opioid administration and a subsequent lower rate of new persistent opioid use, fewer opioid prescription refills, and fewer cases of chronic pain as far out as 12 months after surgery. These results raise important questions that require randomized clinical trials to verify causality between intraoperative opioid administration and long-term outcomes.

The structure and methodology of this study can be adapted for other exposures and outcomes, and we intend to analyze the effects of nonopioid and regional anesthetic treatment modalities in perioperative pain management. Further follow-up studies in the form of randomized clinical trials are also required to investigate the relationship between the timing of opioid administration and pain-related outcomes.

The opioid crisis presents a clear need to reduce overall opioid exposure to reduce the risk of dependence. However, the overgeneralization of this imperative to the operating room may have led to worse outcomes. While our results suggest that intraoperative opioids are currently being underdosed on average, in similar fashion we would not want to overgeneralize. Following the recommendation of the American Pain Society^12^ that clinicians evaluate each patient on an individual basis, we propose that further work is needed to develop objective measures to support optimal intraoperative opioid administration on a personalized basis.

## Notes

Supplement 1. eMethods eTable 1. Model coefficients for adjusted model eTable 2. Most frequent CPT codes in study cohort eFigure 1. Forest plot for models without ICD and CPT adjustment eFigure 2. Forest plot for interaction models without ICD and CPT adjustment eFigure 3. Forest plot for interaction models with ICD and CPT adjustment eFigure 4. Forest plot for subgroup analysis in patients with BMI >30 without ICD and CPT adjustment eFigure 5. Forest plot for subgroup analysis in patients with BMI >30 with ICD and CPT adjustment eFigure 6. Intraoperative opioid administration timing eFigure 7. Pain distributions (24h, in hospital) eFigure 8. Frequency of pain assessments in each outcome window eFigure 9. Intraoperative opioid administration by year eFigure 10. Total dosage of intraoperative opioid administration

Supplement 2. Data sharing statement
