# Supplementary material for: Association of Intraoperative Opioid Administration With Postoperative Pain and Opioid Use
Source: JAMA Surg. 2023 Jun 14;158(8):854–64. doi: 10.1001/jamasurg.2023.2009 (PMC10267849; doi:10.1001/jamasurg.2023.2009)
Supplement: Supplement 1. — eMethods eTable 1. Model coefficients for adjusted model eTable 2. Most frequent CPT codes in study cohort eFigure 1. Forest plot for models without ICD and CPT adjustment eFigure 2. Forest plot for interaction models without ICD and CPT adjustment eFigure 3. Forest plot for interaction models with ICD and CPT adjustment eFigure 4. Forest plot for subgroup analysis in patients with BMI >30 without ICD and CPT adjustment eFigure 5. Forest plot for subgroup analysis in patients with BMI >30 with ICD and CPT adjustment eFigure 6. Intraoperative opioid administration timing eFigure 7. Pain distributions (24h, in hospital) eFigure 8. Frequency of pain assessments in each outcome window eFigure 9. Intraoperative opioid administration by year eFigure 10. Total dosage of intraoperative opioid administration [file jamasurg-e232009-s001.pdf]

Supplemental Online Content

Santa Cruz Mercado LA, Liu R, Bharadwaj KM, et al. Association of intraoperative opioid administration with postoperative pain and opioid use. *JAMA Surg*. Published online June 14, 2023. doi:10.1001/jamasurg.2023.2009

eMethods

eTable 1. Model coefficients for adjusted model

eTable 2. Most frequent CPT codes in study cohort

eFigure 1. Forest plot for models without ICD and CPT adjustment

eFigure 2. Forest plot for interaction models without ICD and CPT adjustment

eFigure 3. Forest plot for interaction models with ICD and CPT adjustment

eFigure 4. Forest plot for subgroup analysis in patients with BMI >30 without ICD and CPT adjustment

eFigure 5. Forest plot for subgroup analysis in patients with BMI >30 with ICD and CPT adjustment

eFigure 6. Intraoperative opioid administration timing

eFigure 7. Pain distributions (24h, in hospital)

eFigure 8. Frequency of pain assessments in each outcome window

eFigure 9. Intraoperative opioid administration by year

eFigure 10. Total dosage of intraoperative opioid administration

This supplemental material has been provided by the authors to give readers additional information about their work.

© 2023 Santa Cruz Mercado LA et al. *JAMA Surgery.*

eMethods

A. Patient Selection Procedures
1. Data extraction
2. Painful vs non-painful surgeries

B. Variable Definitions
1. Summary of variables
2. Drug dictionary
3. MME conversion table
4. Ideal Body Weight Calculation
5. Time stamp definitions
6. ICD code definitions

C. Assessment of Minimal Clinically Important Difference (MCID)

D. Verification procedures

E. Pk/PD Model Structures

F. Adjustment for Bias and Confounding
1. DAG
2. Baseline variables
3. Intraoperative case characteristics
4. Additional adjustment variables
5. Propensity score method

G. Counterfactual estimates

H. Pain Management in PACU

eTables and eFigures

eTable 1. Model coefficients for adjusted model
eTable 2. Most frequent CPT codes in study cohort
eFigure 1. Forest plot for models without ICD and CPT adjustment
eFigure 2. Forest plot for interaction models without ICD and CPT adjustment
eFigure 3. Forest plot for interaction models with ICD and CPT adjustment
eFigure 4. Forest plot for subgroup analysis in patients with BMI >30 without ICD and CPT adjustment
eFigure 5. Forest plot for subgroup analysis in patients with BMI >30 with ICD and CPT adjustment
eFigure 6. Intraoperative opioid administration timing
eFigure 7. Pain distributions (24h, in hospital)
eFigure 8. Frequency of pain assessments in each outcome window
eFigure 9. Intraoperative opioid administration by year
eFigure 10. Total dosage of intraoperative opioid administration

© 2023 Santa Cruz Mercado LA et al. *JAMA Surgery.*

eMethods

A. Patient Selection Procedures

1. Data Extraction

Medical records were extracted from the EPIC database. All data variables from the database were properly identified with a database atlas that included variable names and definitions and queried using SQL. Due to implementation of EPIC at MGH in 2016, we did not have access to data preceding this timepoint. Thus, we chose to begin our patient selection search criteria with timepoints beginning after April 2016. We ended the search in March 2020 to allow for study of 30-, 60- and 180-day post-operative variables. A preliminary search of the database identified 355234 surgical log records. Based upon preidentified exclusionary and inclusionary criteria, we removed pediatric cases (patients under the age of 18), cardiac cases and endoscopy cases. Additionally, we removed cases that would have no CPC or PACU outcome data. This included records where the case was cancelled, where the patient died in the OR, where the patient was transferred to the ICU, or where the case occurred in a non-surgical location such as an electrophysiology lab.

We accessed the data using the Server Query Language (SQL) through the program Microsoft SQL Server Management Studio. As the scope of the study was quite broad, we queried for basic identifiers for all patients meeting the study's inclusion criteria. The inclusion criteria of patients aged 18 years or older, date of surgery within April 2016 to March 2020, non-cardiac surgeries/procedures, and transferal to ICU were direct filters to implement in the query. As there were many cases of minor procedures such as endoscopies, which are excluded per the exclusion criteria, we also filtered out cases based on location and service name to significantly improve query performance. As noted in the following section, we were unable to easily exclude all non-painful surgeries/procedures and later had to do a more careful search with keywords. Due to the mislabeling of non-general anesthesia cases, we created an algorithm to identify true general anesthesia cases within the database from mislabeled cases. We defined general anesthesia as the presence of any one of the following criteria between the 'In Room' and 'Out of Room' time points within the general anesthesia record in EPIC: Intubation Time Point, Extubation Time Point, LMA placement/removal or ETT tube placement and removal. We also included cases that were flagged as having an intubation. After removing cases that did not meet these criteria, we were left with 107298 surgical cases who went to the PACU or CPC after surgery. These cases were further studied to determine those that met the selection criteria. From this patient list, we then extracted all associated patient data and applied the remaining exclusion criteria in our MATLAB data processing pipeline.

2. Painful vs. Non-painful Surgeries/Procedures

To best address our research question, we wanted to ensure the exclusion of non-painful, non-surgical cases where the standard practice would not require administration of opioids intra-operatively. To identify, select and exclude non-painful procedures, two clinical anesthesiologists constructed and reviewed a list of 58 keywords to help identify non-painful procedures. If any of the keywords was contained in the name of the procedure, as listed in the medical record, it was marked as a non-painful and excluded from the analysis.

<u>Keywords for non-painful procedures:</u> wash, need*, close, lithotripsy, angiogram, endoscopy, dilatation, examination, dressing, vac, embolization, eua, esophagogastroduodenoscopy, sclerotherapy, ebus, egd, sigmoidoscopy flexible, cone, leep, polyp, arteriovenous fistula, colonoscopy, anoscopy, banding, pouchoscopy, Bartholin, marsupialization, manipulation, ligation of inter, turbt, fulguration, suspension, botox, steroid, macroplastique, sclerotherapy, injection, tbbx, port-a-cath, port, ommaya reservoir, transjugular, insertion, vagal, battery, scar, tips, anal canal, biopsy bladder, anoscopy with biopsy, anoscopy and biopsy Nasopharyngoscopy, esophagoscopy, cystosc*, cystoscopy, bronc*, colonoscopy, endoscopic.

For cases not classified by the previous keywords, two clinical anesthesiologists reviewed the cases and classified them to be excluded if any of the following were true:

- Procedure name was unclear or there was no procedure name.
- The procedure could be otherwise done at the doctor's office.
- Standard practice would not require administration of opioids intra-operatively.

B. Variable Definitions

1. Summary of variables

Group	Variable Name	Definition	Extraction Summary	Variable Type
**EXP.1 Primary Exposure**				
**EXP.1**	**Intraoperative Fentanyl**	PK/PD modeling was used to evaluate the effect site concentration of Fentanyl across the surgical window. The area under the curve (AUC) was evaluated and divided by time, effectively calculating the time weighted average of the effect site concentration of Fentanyl across the surgical period.	Drug records on day of surgery	Numeric
**EXP.1**	**Intraoperative Hydromorphone**	PK/PD modeling was used to evaluate the effect site concentration of Hydromorphone across the surgical window. The area under the curve (AUC) was evaluated and divided by time, effectively calculating the time weighted average of the effect site concentration of Hydromorphone across the surgical period.	Drug records on day of surgery	Numeric
**EXP.2 Other Exposures**				
**EXP.2**	**Blocks/Epidural Use**	Use of blocks or epidurals on the day of surgery.	Block and epidural records on day of surgery	Binary
**EXP.2**	**PCA Use**	Use of patient-controlled anesthesia (PCA) on the day of surgery.	Drug records on day of surgery	Binary
**FAC Measurement Facets**				
**FAC**	**CPC Vs PACU**	Whether the maximum pain score recorded was taken in the PACU, CPC, or another location.	Location records	Categorical
**FAC**	**Phase Of Care**	Whether the maximum pain score recorded was taken during Phase 1, Phase 2, or another phase of postoperative care.	Timestamp records	Categorical
**FAC**	**Time Of Day**	The time of day that the maximum pain score was recorded. Bins of 4hr windows	Timestamp of pain score	Categorical
**FAC**	**Midpoint of PACU stay**	The midpoint of PACU stay (4-hour bins). Time of day and day of week for the midpoint for MME outcome.	Timestamp records	Categorical
**FAC**	**Day Of Week**	The day of the week that the maximum pain score was recorded, with Sunday being 1.	Timestamp of pain score	Categorical
**FAC**	**Numeric Vs String**	Whether the pain score was directly recorded numerically (0-10) or was converted from the visual/pain score.	Pain score records on day of surgery	Binary

© 2023 Santa Cruz Mercado LA et al. *JAMA Surgery*.

**IOC.1 Other intraoperative opioid administration**				
**IOC.1**	**Intra-op Non-Analgesic Opioids**	Total MME/time of non-analgesic opioids administered intraoperatively (See section B2 – Drug dictionary)	Drug records	Numeric
**IOC.2 Other medications administered**				
**IOC.2**	**Vasopressors**	NE (in mcg)/time equivalent amount of vasopressor administered intraoperatively (See section B2 – Drug dictionary)	Drug records	Numeric
**IOC.2**	**CYP3A4 Inducers**	Exposure to CYP3A4 inducer medication intraoperatively (See section B2 – Drug dictionary)	Drug records	Binary
**IOC.2**	**CYP3A4 Inhibitors**	Exposure to CYP3A4 inhibitor medication intraoperatively (See section B2 – Drug dictionary)	Drug records	Binary
**IOC.2**	**Antihypertensive Drugs**	Exposure to antihypertensive medication intraoperatively (See section B2 – Drug dictionary)	Drug records	Binary
**IOC.2**	**Naloxone**	Exposure to Naloxone: For Max Pain outcome = exposure start-max pain in PCU; For MME outcome= Exposure start-end of PACU stay (See section B2 – Drug dictionary)	Drug records	Binary
**IOC.2**	**Analgesics And Anesthetic Adjuncts**	Exposure to non-opioid analgesics and anesthetic adjunct medication intraoperatively (See section B2 – Drug dictionary)	Drug records	Numeric vector
**IOC.3 Adverse Clinical Events**				
**IOC.3**	**Hypotensive Duration**	The total number of minutes that a patient is hypotensive during the surgery, calculated by comparing the baseline systolic/diastolic blood pressure against the blood pressure across the duration of surgery. The baseline blood pressure is defined as either the last blood pressure recording prior to surgical visit, or if unavailable, the initial blood pressure recording on the day of surgery. A 20% decrease from either the systolic or diastolic baseline is considered hypotensive.	Blood pressure records	Numeric
**IOC.3**	**Hypertensive Duration**	The total number of minutes that a patient is hypertensive during the surgery, calculated by comparing the baseline systolic/diastolic blood pressure against the blood pressure across the duration of surgery. The baseline blood pressure is defined as either the last blood pressure recording prior to surgical visit, or if unavailable, the initial blood pressure recording on the day of surgery. A 20% increase from either the systolic or diastolic	Blood pressure records	Numeric

© 2023 Santa Cruz Mercado LA et al. *JAMA Surgery.*

		baseline is considered hypertensive.		
**IOC.3**	**Tachycardic Duration**	The total number of minutes that a patient is tachycardic during the surgery, calculated by checking the heart rate across the duration of surgery. A heart rate of > 100 bpm is considered tachycardic.	Heart rate records	Numeric
**IOC.3**	**Bradycardic Duration**	The total number of minutes that a patient is bradycardic during the surgery, calculated by checking the heart rate across the duration of surgery. A heart rate of < 60 bpm is considered bradycardic.	Heart rate records	Numeric
**IOC.4 Anesthesia Characteristics**				
**IOC.4**	**General Anesthesia Type**	The type(s) of general anesthesia used intraoperatively, flagged as binary values. Order as follows: N2O, Desflurane, Isoflurane, Propofol, Sevoflurane.(See section B2 – Drug dictionary)	Anesthetic records on day of surgery	Binary vector
**IOC.4**	**Anesthetic Dose**	The dosage amounts of the general anesthesia type(s) administered during the surgery. For inhalatory agents, we calculated the time-weighted average of the end tidal concentration. For propofol we calculated the total infuse dose.	Anesthetic records on day of surgery	Numeric vector
**IOC.4**	**Neuromuscular Blocking Agents**	Exposure to anesthesia neuromuscular blocking agent medication intraoperatively (See section B2 – Drug dictionary).	Drug records on day of surgery	Binary
**Other intra-operative characteristics**				
**IOC.5**	**Intraoperative Blood Pressure**	The time weighted average systolic and diastolic blood pressure intraoperatively.	Blood pressure records	Numeric
**IOC.5**	**Intraoperative Heart Rate**	The time weighted average heart rate intraoperatively.	Heart rate records	Numeric
**IOC.5**	**Surgical Duration**	The length of surgery in minutes. (See section B5 – Time stamp definitions)	Time Stamps	Numeric
**IOC.5**	**Fluids Given**	The amount of fluids given intraoperatively (total mL).	Fluids records	Numeric
**IOC.5**	**Blood Loss**	The amount of blood lost intraoperatively (total mL).	Surgical records	Numeric
**IOC.5**	**Intraoperative Transfusion**	Blood transfusion administered intraoperatively.	Transfusion records	Binary
**IOC.5**	**Conversion**	Whether the patient underwent a laparoscopic surgery that was converted to open. If yes, then 1, otherwise 0.	Surgery name records	Binary

**IOC.5**	**Difficult Intubation**	Presence of specified Difficult intubation in the anesthesia record.	Anesthestic records on day of surgery	Binary
**POC.1 Primary Outcome**				
**POC.1**	**PACU Total MME**	The total MME administered within the PACU window. (See section B5 – Time stamp definitions)	Drug records on day of surgery	Numeric
**POC.1**	**PACU Max Pain Score**	The maximum pain score within the PACU window. (See section B5 – Time stamp definitions)	Pain score records on day of surgery using the Numeric Rating Scale (values from 0 to 10). Sometimes clinicians recorded 0 pain as "no pain" string variable.	Categorical
**POC.2 Short-term Outcomes**				
**POC.2**	**In Hospital Total MME**	The total MME administered postoperatively to discharge. (See section B5 – Time stamp definitions)	Drug records	Numeric
**POC.2**	**Frequency Of Uncontrolled Pain**	The number of acute pain consults requested postoperatively.	Acute pain consults notes	Binary
**POC.2**	**Postop 24 Hour MME**	The total MME administered postoperatively in a 24-hour window. (See section B5 – Time stamp definitions)	Drug records	Numeric
**POC.2**	**PACU Length of Stay**	Length of stay in the PACU in minutes.	PACU end - PACU start	Numeric
**POC.2**	**Hospital Length of Stay**	Length of stay from admission to discharge in minutes.	Discharge - Admission	Numeric
**POC.2**	**Postop Opioid Complications in PACU: PONV**	Presence of PONV in PACU (See section B6 – ICD definitions)	PACU flowsheet	Binary
**POC.2**	**Postop Opioid Complications in PACU: Sedation**	Time required to reach a Modified Aldrete consciousness score of 2 or RASS 0 or 1 in PACU.	PACU flowsheet	Numeric
**POC.2**	**Postop Opioid Complications in PACU: Respiratory depression**	Respiratory depression will be defined as a Modified Aldrete respiratory score of < 2	PACU flowsheet	Binary
**POC.3 Long-term Outcomes**				
**POC.3**	**3-Month Chronic Pain**	Presence of a postoperative chronic pain diagnosis within 3 months after surgery.	ICD records postoperatively	Binary
**POC.3**	**12-Month Chronic Pain**	Presence of a postoperative chronic pain diagnosis within 12 months after surgery.	ICD records postoperatively	Binary
**POC.3**	**30-Day Opioid Prescription**	Presence of an opioid prescription from 0-30 days postoperatively.	Prescription Records	Binary
**POC.3**	**90-Day Opioid Prescription**	Presence of an opioid prescription from 0-90 days postoperatively.	Prescription Records	Binary
**POC.3**	**180-Day Opioid Prescription**	Presence of an opioid prescription from 0-180 days postoperatively.	Prescription Records	Binary
**POC.3**	**Persistent Use**	Define as the presence of a new opioid prescription from 90-180 days postoperatively.	Presence of drug in intraoperative window	Binary

© 2023 Santa Cruz Mercado LA et al. *JAMA Surgery.*

**POC.3**	**30-Day Postop Mortality**	Indicated if a patient dies within 30 days after surgery.	Death records 30 days after date of surgery	Binary
**POC.3**	**30-Day Postop Readmission**	Indicated if a patient is readmitted within 30 days after surgery.	Admission records 30 days after date of surgery	Binary
**PRBV Provider Baseline Variables**				
**PRBV**	**Provider Experience**	How experienced is the provider associated with the surgery, categorized into low, medium, and high levels of experience. The number of cases for each provider across MGH was measured, and the 25th and 75th percentiles were evaluated. Providers that fell under the 25th percentile of cases (1906) were marked as low experience, providers that fell between the 25th and 75th (4325) percentile were marked as medium experience, and providers that fell on or above the 75th percentile were marked as high experience.	Provider records associated with surgeries	Categorical
**PRBV**	**RN Anesthetist Experience**	How experienced is the nurse anesthetist associated with the surgery, categorized into low, medium, and high levels of experience. The number of cases for each nurse across MGH was measured, and the 25th and 75th percentiles were evaluated. Nurses that fell under the 25th percentile of cases (1997) were marked as low experience, nurses that fell between the 25th and 75th (3548) percentile were marked as medium experience, and nurses that fell on or above the 75th percentile were marked as high experience.	Nurse anesthetist records associated with surgery.	Categorical
**PTBV.1 Demographics**				
**PTBV.1**	**Age**	The age of the patient on the day of surgery.	Date of service timestamp - date of birth timestamp	Numeric
**PTBV.1**	**Gender**	The gender (male or female) of the patient on the day of the surgery.	Gender record	Binary
**PTBV.1**	**Race**	The race of the patient on the day of surgery	Race record	Categorical
**PTBV.1**	**Ethnic Group**	The ethnic group (hispanic or nonhispanic) on the day of surgery.	Ethnic group record	Categorical
**PTBV.1**	**Preferred Language**	The preferred language (English/Non-English) of the patient on the day of surgery.	Preferred language record	Binary
**PTBV.1**	**BMI**	The BMI of the patient on the day of surgery. If the BMI is unavailable on the day of surgery, the BMI last recorded was obtained.	BMI records	Numeric

© 2023 Santa Cruz Mercado LA et al. *JAMA Surgery.*

**PTBV.1**	**Height**	The height of the patient on the day of surgery (in inches). If the height is unavailable on the day of surgery, the height last recorded was obtained.	Height records	Numeric
**PTBV.1**	**Weight**	The weight of the patient on the day of surgery (in kg). If the weight is unavailable on the day of surgery, the weight last recorded was obtained.	Height and weight records	Numeric
**PTBV.1**	**IBW**	The calculated IBW (or for obese patients, the ABW) using the formula as defined in section B4.	Height and weight records	Numeric
**PTBV.2 Medical History**				
**PTBV.2**	**Tobacco Use History**	Presence of any specified ICD codes (See section B6) across the 6 months prior to surgery.	ICD records 6 months prior to surgery to DOS	Binary
**PTBV.2**	**Alcohol Use History**	Presence of any specified ICD codes (See section B6) across the 6 months prior to surgery.	ICD records 6 months prior to surgery to DOS	Binary
**PTBV.2**	**Non-Opioid Substance Abuse History**	Presence of any specified ICD codes (See section B6) across the 6 months prior to surgery.	ICD records 6 months prior to surgery to DOS	Binary
**PTBV.2**	**Opioid Abuse History**	Presence of any specified ICD codes (See section B6) across the 6 months prior to surgery.	ICD records 6 months prior to surgery to DOS	Binary
**PTBV.2**	**Chronic pain History**	Presence of any specified ICD codes (See section B6) across the 6 months prior to surgery.	ICD records 6 months prior to surgery to DOS	Binary
**PTBV.2**	**Anxiety History**	Presence of any specified ICD codes (See section B6) across the 6 months prior to surgery.	ICD records 6 months prior to surgery to DOS	Binary
**PTBV.2**	**Depression History**	Presence of any specified ICD codes (See section B6) across the 6 months prior to surgery.	ICD records 6 months prior to surgery to DOS	Binary
**PTBV.2**	**Other Psychiatric History**	Presence of any specified ICD codes (See section B6) across the 6 months prior to surgery.	ICD records 6 months prior to surgery to DOS	Binary
**PTBV.2**	**Neurological History**	Presence of any specified ICD codes (See section B6) across the 6 months prior to surgery.	ICD records 6 months prior to surgery to DOS	Binary
**PTBV.2**	**Dialysis History**	Presence of any specified ICD codes (See section B6) across the 6 months prior to surgery.	ICD records 6 months prior to surgery to DOS	Binary
**PTBV.2**	**Other Kidney Disorder History**	Presence of any specified ICD codes (See section B6) across the 6 months prior to surgery.	ICD records 6 months prior to surgery to DOS	Binary
**PTBV.2**	**Chronic Renal Failure History**	Presence of any specified ICD codes (See section B6) across the 6 months prior to surgery.	ICD records 6 months prior to surgery to DOS	Binary
**PTBV.2**	**Acute Renal Failure History**	Presence of any specified ICD codes (See section B6) within 24 hours prior to surgery.	ICD records 24 hours prior to surgery to DOS	Binary
**PTBV.2**	**Liver Disease History**	Presence of any specified ICD codes (See section B6) across the 6 months prior to surgery.	ICD records 6 months prior to surgery to DOS	Binary

© 2023 Santa Cruz Mercado LA et al. *JAMA Surgery.*

**PTBV.2**	**Cancer History**	Presence of any specified ICD codes (See section B6) across the 6 months prior to surgery.	ICD records 6 months prior to surgery to DOS	Binary
**PTBV.2**	**DM2 History**	Presence of any specified ICD codes (See section B6) across the 6 months prior to surgery.	ICD records 6 months prior to surgery to DOS	Binary
**PTBV.2**	**Rheumatological History**	Presence of any specified ICD codes (See section B6) across the 6 months prior to surgery.	ICD records 6 months prior to surgery to DOS	Binary
**PTBV.2**	**Opioid Allergy History**	Presence of any specified ICD codes (See section B6) across the 6 months prior to surgery.	ICD records 6 months prior to surgery to DOS	Binary
**PTBV.2**	**OSA History**	Presence of any specified ICD codes (See section B6) across the 6 months prior to surgery.	ICD records 6 months prior to surgery to DOS	Binary
**PTBV.2**	**Pulmonary Disease History**	Presence of any specified ICD codes (See section B6) across the 6 months prior to surgery.	ICD records 6 months prior to surgery to DOS	Binary
**PTBV.2**	**Difficult Intubation History**	Presence of any specified ICD codes (See section B6) across the 6 months prior to surgery.	ICD records 6 months prior to surgery to DOS	Binary
**PTBV.2**	**Hematological History**	Presence of any specified ICD codes (See section B6) across the 6 months prior to surgery.	ICD records 6 months prior to surgery to DOS	Binary
**PTBV.2**	**Chronic Steroid Use History**	Presence of any specified ICD codes (See section B6) across the 6 months prior to surgery.	ICD records 6 months prior to surgery to DOS	Binary
**PTBV.2**	**Systemic Sepsis History**	Presence of any specified ICD codes (See section B6) within 48 Hours Prior to Surgery	ICD records 48 hours prior to surgery to DOS	Binary
**PTBV.2**	**PONV History**	Presence of specified ICD codes (See section B6) across the 6 months prior to surgery.	ICD records 6 months prior to surgery to DOS	Binary
**PTBV.2**	**Communication Impairment History**	Presence of any specified ICD codes (See section B6) across the 6 months prior to surgery.	ICD records 6 months prior to surgery to DOS	Binary
**PTBV.2**	**Dementia History**	Presence of specified ICD codes (See section B6) across the 6 months prior to surgery.	ICD records 6 months prior to surgery to DOS	Binary
**PTBV.2**	**Delirium History**	Presence of specified ICD code (See section B6) across the 6 months prior to surgery.	ICD records 6 months prior to surgery to DOS	Binary
**PTBV.2**	**Mild Cognitive Impairment History**	Presence of any specified ICD codes (See section B6) across the 6 months prior to surgery.	ICD records 6 months prior to surgery to DOS	Binary
**PTBV.2**	**Other Pain Diagnosis History**	Presence of any specified ICD codes (See section B6) across the 6 months prior to surgery.	ICD records 6 months prior to surgery to DOS	Binary
**PTBV.2**	**Cardiac any History**	Presence of specified ICD codes (See section B6) across the 6 months prior to surgery.	ICD records 6 months prior to surgery to DOS	Binary
**PTBV.3 Surgical History**				
**PTBV.3**	**Surgical Complication History**	Presence of specified ICD codes (See section B6) across the 6 months prior to surgery.	ICD records 6 months prior to surgery to DOS	Binary
**PTBV.3**	**Past Surgical History**	Presence of CPT codes 6 months prior to surgery	CPT records for the surgeries 6 months prior to date of surgery	Binary

© 2023 Santa Cruz Mercado LA et al. *JAMA Surgery.*

**PTBV.3**	**Intraoperative Opioid Exposure History**	Exposure to intraoperative opioid use 6 months prior to surgery	Record of intraoperative opioid use 6 months prior to date of surgery	Binary
**PTBV.3**	**General Anesthesia Exposure History**	Exposure to general anesthesia 6 months prior to surgery	Record of general anesthesia use 6 months prior to date of surgery	Binary
**PTBV.4 Risk Indexes**				
**PTBV.4**	**Frailty Index**	An adaptation of the frailty index defined in Hope, et al. 2015: “Frailty Before Critical Illness and Mortality for Elderly Medicare Beneficiaries.” ICD codes relevant to 12 categories of frailty as listed in Hope et al. were compiled and checked against patient records over the past 6 months. Patients with ICD codes matching within a category were given a point, for up to 12 indicating the highest frailty. (See section B6)	ICD records 6 months prior to surgery to date of surgery	Numeric
**PTBV.4**	**Procedural Severity Score**	An adaptation of the Procedural Severity Score as defined in Dalton, et al 2011: “Development and Validation of a Risk Quantification Index for 30-Day Postoperative Mortality and Morbidity in Noncardiac Surgical Patients.” CPT codes were assigned a mortality and morbidity risk, referred to as a Procedural Severity Score (PSS). CPT codes associated with patients were referenced against the PSS.	PSS table from Dalton supplement, CPT record	Numeric vector
**PTBV.4**	**Comorbidity Index**	The Elixhauser comorbidity index has 31 distinct categories with associated ICD codes. ICD codes assigned to patients over the past 6 months were compared against each category, with a match within a category adding to the index score (See section B6).	ICD records 6 months prior to surgery to date of surgery	Numeric
**PTBV.4**	**ASA Status**	The recorded ASA status from the patient record on the day of surgery	ASA status record	Categorical
**PTBV.5 Baseline Medication Exposure**				
**PTBV.5**	**Baseline Opioid Use**	Exposure to opioids 6 months prior to surgery	Drug records 6 months prior to surgery	Binary
**PTBV.5**	**Opioid Naivety**	A patient is considered as "naïve" to opioids if they were not exposed to opioids between 1 year and 31 days prior to surgery.	Drug records 1 year to 31 days prior to surgery	Binary
**PTBV.5**	**Baseline Non-Opioid Medication Use**	Exposure to specific medications (See section B2) that are nonopioids but have analgesic or other associated effects.	Drug records 6 months prior to surgery	Binary

© 2023 Santa Cruz Mercado LA et al. *JAMA Surgery.*

**PTBV.5**	**Baseline Benzodiazepine Use**	Exposure to benzodiazepines 1 year prior to surgery	Drug records 1 year prior to surgery	Binary
**PTBV.5**	**Baseline Opioid Antagonists Use**	Exposure to opioid antagonist medication intraoperatively (See section B2)	Drug records on day of surgery	Binary
**PTBV.6 Baseline Clinical Measures**				
**PTBV.6**	**Pain Score at Last Visit**	A patient's pain score (0-10) recorded in their most recent clinical visit prior to surgery. Patients without a recorded pain score prior to surgery are binned separately.	Pain score records prior to day of surgery	Categorical
**PTBV.7 Pre-operative Interventions**				
**PTBV.7**	**Anxiolysis Pre-medication**	Exposure to anxiolysis medication preoperatively (See section B2) no more than 4 hours before surgery	Drug records on day of surgery	Binary
**PTBV.7**	**Preoperative Transfusion**	Blood transfusion administered preoperatively.	Transfusion records	Binary
**PTBV.7**	**Preemptive Non-Opioid analgesia**	Exposure to non-opioid analgesic medication preoperatively the day of surgery (See section B2)	Drug records on day of surgery	Binary
**PTBV.7**	**Preemptive Opioid Analgesia**	Exposure to opioid medication preoperatively (See section B2)	Drug records on day of surgery	Binary
**PTBV.7**	**Preoperative CYP3A4 Inducers**	Exposure to CYP3A4 inducer medication preoperatively (See section B2)	Drug records on day of surgery	Binary
**PTBV.7**	**Preoperative CYP3A4 Inhibitors**	Exposure to CYP3A4 inhibitor medication preoperatively (See section B2)	Drug records on day of surgery	Binary
**PTBV.8 Pre-operative Clinical Measures**				
**PTBV.8**	**Preop Pain Score**	A patient's pain score (0-10) recorded preoperatively. Patients without a preoperative pain score are binned separately.	Pain score records on day of surgery	Categorical
**SBV Surgical Baseline Variables**				
**SBV**	**Surgery Urgency**	Whether a particular surgery is defined as emergent, urgent, non-urgent, or elective. If patient records have the surgery urgency recorded, the record is used. Otherwise, the scheduled start time is compared to the actual procedure start time. Within 1 hr: Emergent, within 4 hrs: Urgent, within 24 hrs: non-urgent, otherwise elective.	Surgery Urgency record, procedure start timestamp, scheduled start timestamp	Categorical
**SBV**	**Inpatient Vs Ambulatory**	Whether a patient is classified as inpatient (1) or ambulatory (0).	Record for inpatient or ambulatory	Binary
**SBV**	**Laparoscopic Vs Open**	Whether the procedure the patient underwent was laparoscopic (1), open (0), or if the patient had a combination of open and laparoscopic procedures (2).	Surgery name records and laparoscopic flag	Categorical
**SBV**	**Surgery Indication**	The ICD code(s) associated with the procedure.	ICD records associated with indication for surgery	Categorical Vector

© 2023 Santa Cruz Mercado LA et al. *JAMA Surgery.*

**SBV**	**CPT Code**	The CPT code(s) associated with the procedure.	CPT records for the surgeries	Categorical Vector
**SBV**	**Surgery Service**	The surgical service (out of 24 possible services) that the particular surgery was classified under.	String Label	Categorical
**SBV**	**ERAS**	Whether the case underwent ERAS protocols, as defined by whether the date of surgery fell on or after ERAS implementation for the surgery type. If so, then 1, otherwise 0.	Date of service timestamp	Binary
**SBV**	**Year Of Surgery**	The year of surgery (2016-2020)	Date of service timestamp	Categorical
**TIME Time-Stamps**				
**TIME**	**Preop Start**	See section B5	Hospital Admission	DateTime
**TIME**	**Preop End**	See section B5	AnesthesiaStartDTS, AnesthesiaStart, or Start Data Collection	DateTime
**TIME**	**Preop Time End**	See section B5	Patient In Room	DateTime
**TIME**	**Surgical Duration Start**	See section B5	ProcedureStartDTS	DateTime
**TIME**	**Surgical Duration End**	See section B5	ProcedureCompletedDTS	DateTime
**TIME**	**Exposure Start**	Start of the intra-operative anesthesia care	ProcedureStartDTS	DateTime
**TIME**	**Exposure End**	End of intra-operative anesthesia care	Extubation or stop of CO2 Ventilator + 10 minutes	DateTime
**TIME**	**Anesthesia Maintenance Start**	See section B5	Time of first sedative or hypnotic administration	DateTime
**TIME**	**Anesthesia Maintenance End**	See section B5	Time at which last sedative or hypnotic is discontinued	DateTime
**TIME**	**Post Emergence Opioid Administration Start**	See section B5	Extubation or Stop of CO2 Ventilator + 10 minutes	DateTime
**TIME**	**Post Emergence Opioid Administration End**	See section B5	InPhase1 or In PACU	DateTime
**TIME**	**PACU Hold Start**	See section B5	Extubation or Stop of CO2 Ventilator	DateTime
**TIME**	**PACU Hold End**	See section B5	InPhase1 or In PACU	DateTime
**TIME**	**PACU Start**	See section B5	InPhase1 or In PACU	DateTime
**TIME**	**PACU End**	See section B5	Ready for PACU Discharge	DateTime
**TIME**	**In Hospital Start**	See section B5	Hospital Admission	DateTime
**TIME**	**In Hospital End**	See section B5	Hospital Discharge	DateTime
**TIME**	**Post Op 24-hour Start**	See section B5	InPhase1 or In PACU	DateTime
**TIME**	**Post Op 24-hour End**	See section B5	24 Hours post In PACU	DateTime
**TIME**	**In Hospital Postop Start**	See section B5	Ready for PACU Discharge	DateTime
**TIME**	**In Hospital Postop End**	See section B5	Hospital Discharge	DateTime
**ADJ.1 Adjustment variables for Max Pain Outcome**				

© 2023 Santa Cruz Mercado LA et al. *JAMA Surgery.*

**ADJ.1**	**Effect site concentration of fentanyl and hydromorphone at first max-pain**	PK/PD modeling was used to evaluate the effect site concentration of fentanyl and or hydromorphone at the moment of first max-pain score in PACU.	Drug records on day of surgery	Numeric
**ADJ.1**	**Cumulative MME for opioids given after exposure end and before first max-pain in PACU**	Cumulative MME for opioids given between the end of the exposure window and first Max Pain in PACU (except hydromorphone and fentanyl)	Drug records on day of surgery	Numeric
**ADJ.1**	**Non-Analgesic Opioids administered in PACU**	Total MME/time of non-analgesic opioids administered in PACU (See section B2)	Drug records on day of surgery	Numeric
**ADJ.1**	**Postoperative Non-Opioid Analgesics in PACU**	Exposure to non-opioid analgesic medication in PACU (See section B2)	Drug records on day of surgery	Numeric vector
**ADJ.2 Adjustment variables for Total MME in PACU outcome**				
**ADJ.2**	**Effect site concentration of fentanyl and hydromorphone at PACU admission**	PK/PD modeling was used to evaluate the effect site concentration of fentanyl and or hydromorphone at PACU admission.	Drug records on day of surgery	Numeric
**ADJ.2**	**Antiemetic Prophylaxis**	Exposure to antiemetic prophylaxis medication preoperatively or intraoperatively (See section B2)	Drug records on day of surgery	Binary
**ADJ.2**	**PONV in PACU**	Instances of PONV recorded during PACU stay	PACU flowsheet	Binary
**ADJ.2**	**Pasero Opioid-induced Sedation Scale (POSS)**	POSS variable as a binary variable = "1" if score >= 3 for any POSS score recorded during PACU stay.	PACU flowsheet	Binary
**ADJ.2**	**Post-operative Non-Opioid Analgesics in PACU**	Exposure to non-opioid analgesic medication in PACU (See section B2)	Drug records on day of surgery	Numeric vector

© 2023 Santa Cruz Mercado LA et al. *JAMA Surgery.*

2. **Drug dictionary**
   Certain variables depend on identifying administration of specific drugs. Below, drug lists for variables are provided. Data extraction and processing involved filtering for these drug labels.

   a. **Intraop Opioid Exposure Hx:** Fentanyl, Hydromorphone, Morphine, Methadone, Oxycodone, Meperidine, Remifentanil, Codeine, Hydrocodone, Oxymorphone, Sufentanil, Alfentanil, Nalbuphine, Buprenorphine, Butorphanol, Levorphanol, Pentazocine, Tramadol, Tapentadol, Dihydrocodeine, Opium, Paregoric

   b. **General Anesthesia Exposure Hx**: Nitrous Oxide, Propofol, Desflurane, Isoflurane, Sevoflurane

   c. **Baseline Opioid Use:** Fentanyl, Hydromorphone, Morphine, Methadone, Oxycodone, Meperidine, Remifentanil, Codeine, Hydrocodone, Oxymorphone, Sufentanil, Alfentanil, Nalbuphine, Buprenorphine, Butorphanol, Levorphanol, Pentazocine, Tramadol, Tapentadol, Dihydrocodeine, Opium, Paregoric

   d. **Baseline Non-Opioid Analgesic Medication Use:** Aspirin, Celecoxib, Diclofenac, Diflunisal, Etodolac, Fenoprofen, Flurbiprofen, Ibuprofen, Indomethacin, Ketorolac, Ketoprofen, Magnesium salicylate, Meclofenamate, Mefenamic acid, Meloxicam, Nabumetone, Naproxen, Oxaprozin, Piroxicam, Salsalate, Sulindac, Tolmetin, Acetaminophen, Gabapentin, Pregabalin, Carbamazepine, Oxacarbamazepine, Valproic acid, Topiramate, Dexamethasone, Prednisone, Amitriptyline, Nortriptyline, Doxepin, Clomipramine, Duloxetine, Venlafaxine, Milnacipran, Desvenlafaxine, Lamotrigine, Cyclobenzaprine, Methocarbamol, Baclofen, Tizanidine, Clonidine, Propranolol, Verapamil, Almotriptan, Eletriptan, Frovatriptan, Naratriptan, Rizatriptan, Sumatriptan, Zolmitriptan, Ketamine, Lidocaine, Pamidronate, Zoledronic acid, Denosumab, Capsaicin,-Diclofenac

   e. **Baseline Benzodiazepine Use:** Alprazolam, Bromazepam, Chlordiazepoxide, Clobazam, Clonazepam, Clorazepate, Diazepam, Estazolam, Flurazepam, Lorazepam, Midazolam, Nitrazepam, Oxazepam, Quazepam, Temazepam, Triazolam

   f. **Anxiolysis Pre-medication:** Midazolam, Lorazepam, Diazepam, Alprazolam, Temazepam, Oxazepam, Hydroxyzine

   g. **Preemptive Non-Opioid analgesia:** a) NSAIDS and cox selective: Aspirin, Celecoxib , Diclofenac, Diflunisal, Etodolac, Fenoprofen, Flurbiprofen, Ibuprofen, Indomethacin, Ketorolac, Ketoprofen, Magnesium salicylate, Meclofenamate, Mefenamic acid, Meloxicam, Nabumetone, Naproxen, Oxaprozin, Piroxicam, Salsalate, Sulindac, Tolmetin; b) Other: Acetaminophen c) Gabapentinoids: Gabapentin, Pregabalin; d) NMDA receptor antagonists: Ketamine.

   h. **Preemptive Opioid Analgesia:** Fentanyl, Hydromorphone, Morphine, Methadone, Oxycodone, Meperidine, Remifentanil, Codeine, Hydrocodone, Oxymorphone, Sufentanil, Alfentanil, Nalbuphine, Buprenorphine, Butorphanol, Levorphanol, Pentazocine, Tramadol, Tapentadol, Dihydrocodeine, Opium, Paregoric

   i. **Antiemetic Prophylaxis:** Dexamethasone (Up to 4mg antiemetic dose), Ondansetron, Dolasetron, Granisetron, Palonosetron, Scopolamine, Droperidol, Haloperidol, Amisulpride, Dimenhydrinate, Diphenhydramine, Perphenazine, Promethazine, Prochlorperazine

   j. **Preop CYP3A4 Inducers:** Apalutamide, Carbamazepine, Enzalutamide, Fosphenytoin, Lumacaftor, Lumacaftor-ivacaftor, Mitotane, Phenobarbital, Phenytoin, Primidone, Rifampin, rifampicin, Bexarotene, Bosentan, Cenobamate, Dabrafenib, Dexamethasone, Efavirenz, Elagolix, Eslicarbazepine, Etravirine, Lorlatinib, Modafinil, Nafcillin, Pexidartinib, Rifabutin, Rifapentine, St. John's wort, Nevirapine, Griseofulvin

   k. **Preop CYP3A4 Inhibitors:** Atazanavir, Ceritinib, Clarithromycin, Cobicistat, Darunavir, Idelalisib, Indinavir, Itraconazole, Ketoconazole, Lonafarnib, Lopinavir, Mifepristone, Nefazodone, Nelfinavir, Ombitasvir-paritaprevir-ritonavir, Ombitasvir-paritaprevir-ritonavir-dasabuvir, Posaconazole, Ritonavir, Saquinavir, Tucatinib, Voriconazole, Amiodarone, Aprepitant, Berotralstat, Cimetidine, Conivaptan, Crizotinib, Cyclosporine, Diltiazem, Duvelisib, Dronedarone, Erythromycin, Fedratinib, Fluconazole, Fosamprenavir, Fosaprepitant, Fosnetupitant-palonosetron, Imatinib, Isavuconazole, isavuconazonium sulfate, Lefamulin, Letermovir, Netupitant, Nilotinib, Ribociclib, Verapamil, Metronidazole

   l. **Pre-Op, Intra-Op or Post-Op Non-Analgesic Opioids:** Meperidine < or = 25 mg (Shivering), Levorphanol, Codeine, Pentazocine, Nalbuphine, Buprenorphine, Butorphanol and Paregoric.

© 2023 Santa Cruz Mercado LA et al. *JAMA Surgery*.

m. **Vasopressors:** Dopamine (100 mcg/kg/min), Ephedrine, Epinephrine (1 mcg/kg/min), Norepinephrine (1 mcg/kg/min), Phenylephrine (10 mcg/kg/min), Vasopressin (0.4 mcg/kg/min). Note that Vasopressors are converted into NE Equivalents. Equivalent values to 1 mcg/kg/min Norepinephrine are in parentheses, with the exception of Ephedrine, where a 10 mg bolus is equivalent to a 5 mcg bolus of Norepinephrine.

n. **CYP3A4 Inducers**: Apalutamide, Carbamazepine, Enzalutamide, Fosphenytoin, Lumacaftor, Lumacaftor-ivacaftor, Mitotane, Phenobarbital, Phenytoin, Primidone, Rifampin, rifampicin, Bexarotene, Bosentan, Cenobamate, Dabrafenib, Dexamethasone, Efavirenz, Elagolix, Eslicarbazepine, Etravirine, Lorlatinib, Modafinil, Nafcillin, Pexidartinib, Rifabutin, Rifapentine, St. John's wort, Nevirapine, Griseofulvin

o. **CYP3A4 Inhibitors:** Atazanavir, Ceritinib, Clarithromycin, Cobicistat, Darunavir, Idelalisib, Indinavir, Itraconazole, Ketoconazole, Lonafarnib, Lopinavir, Mifepristone, Nefazodone, Nelfinavir, Ombitasvir-paritaprevir-ritonavir, Ombitasvir-paritaprevir-ritonavir-dasabuvir, Posaconazole, Ritonavir, Saquinavir, Tucatinib, Voriconazole, Amiodarone, Aprepitant, Berotralstat, Cimetidine, Conivaptan, Crizotinib, Cyclosporine, Diltiazem, Duvelisib, Dronedarone, Erythromycin, Fedratinib, Fluconazole, Fosamprenavir, Fosaprepitant, Fosnetupitant-palonosetron, Imatinib, Isavuconazole, isavuconazonium sulfate, Lefamulin, Letermovir, Netupitant, Nilotinib, Ribociclib, Verapamil, Metronidazole

p. **Antihypertensive Drugs:** Esmolol, Metoprolol, Propranolol, Labetalol, Nicardipine, Clevidipine, Hydralazine, Nitroglycerin, Glyceryl trinitrate, Nitroprusside, Fenoldopam, Verapamil, Diltiazem

q. **Opioid Antagonists:** Naltrexone, Methylnaltrexone, Alvimopam

r. **Analgesics and Anesthetic Adjuncts:** Diclofenac, Ibuprofen, Indomethacin, Ketorolac, Meloxicam, Acetaminophen, Lidocaine, Ketamine, Dexmedetomidine, Esmolol, Magnesium (sulfate), Dexamethasone

s. **General Anesthesia Type/Anesthesia Dose:** Nitrous Oxide, Propofol, Desflurane, Sevoflurane, Isoflurane

t. **Neuromuscular Blocking Agents:** Succinylcholine, Vecuronium, Rocuronium, Pancuronium, Mivacurium, Atracurium, Cisatracurium

u. **PACU Total MME:** Fentanyl, Hydromorphone, Morphine, Methadone, Oxycodone, Meperidine, Remifentanil, Codeine, Hydrocodone, Oxymorphone, Sufentanil, Alfentanil, Nalbuphine, Buprenorphine, Butorphanol, Levorphanol, Pentazocine, Tramadol, Tapentadol, Dihydrocodeine, Opium, Paregoric

v. **24 Hour Postop MME:** Fentanyl, Hydromorphone, Morphine, Methadone, Oxycodone, Meperidine, Remifentanil, Codeine, Hydrocodone, Oxymorphone, Sufentanil, Alfentanil, Nalbuphine, Buprenorphine, Butorphanol, Levorphanol, Pentazocine, Tramadol, Tapentadol, Dihydrocodeine, Opium, Paregoric

w. **30/90/180 Day Postop Prescribed MME/Day:** Fentanyl, Hydromorphone, Morphine, Methadone, Oxycodone, Meperidine, Remifentanil, Codeine, Hydrocodone, Oxymorphone, Sufentanil, Alfentanil, Nalbuphine, Buprenorphine, Butorphanol, Levorphanol, Pentazocine, Tramadol, Tapentadol, Dihydrocodeine, Opium, Paregoric

x. **90-180 Day Postop New Opioid Rx:** Fentanyl, Hydromorphone, Morphine, Methadone, Oxycodone, Meperidine, Remifentanil, Codeine, Hydrocodone, Oxymorphone, Sufentanil, Alfentanil, Nalbuphine, Buprenorphine, Butorphanol, Levorphanol, Pentazocine, Tramadol, Tapentadol, Dihydrocodeine, Opium, Paregoric

y. **PCA Use:** Any drug on day of surgery with PCA tag

z. **Postoperative Non-Opioid analgesia in PACU:** a) NSAIDS and cox selective: Aspirin, Celecoxib, Diclofenac, Diflunisal, Etodolac, Fenoprofen, Flurbiprofen, Ibuprofen, Indomethacin, Ketorolac, Ketoprofen, Magnesium salicylate, Meclofenamate, Mefenamic acid, Meloxicam, Nabumetone, Naproxen, Oxaprozin, Piroxicam, Salsalate, Sulindac, Tolmetin; b) Other: Acetaminophen c) Gabapentinoids: Gabapentin, Pregabalin; d) NMDA receptor antagonists: Ketamine.

© 2023 Santa Cruz Mercado LA et al. *JAMA Surgery.*

3. **MME conversion table**

Below is the conversion to IV Morphine Milligram Equivalents for various opioids. If drug information was found in the database but the route of administration was unspecified and/or did not match a listed route, the general MME conversion for the drug was used.

Drug	Route	Unit	IV MME Conversion	Reference
**Morphine**		mg	0.33	1
	Epidural	mg	10	1
	Intravenous	mg	1	1
	Intrathecal	mg	100	1
	Oral	mg	0.33	1
**Hydromorphone**		mg	1.33	2
	Intravenous	mg	6.7	3
	Oral	mg	1.33	2
**Fentanyl**		mcg	0.04	2
	Intravenous	mcg	0.1	3
	Oral	mcg	0.04	3
	Transdermal	mcg	0.8	2
	Intranasal	mcg	0.05	2
	Buccal	mcg	0.04	2
	Sublingual	mcg	0.04	2
	Intrathecal	mcg	1.25	1,3
**Oxymorphone**		mg	1	2
**Methadone***		mg	≤ 20 mg: 4<br>20-40 mg: 8<br>40-60 mg: 10<br>> 60 mg: 12	4
**Meperidine**		mg	0.03	3
	Intravenous	mg	0.13	3
	Oral	mg	0.03	3
**Sufentanil**		mcg	1	3
**Alfentanil**		mcg	0.015	3
**Remifentanil**		mcg	0.1	3
**Levorphanol**		mg	3.7	2
**Codeine**		mg	0.05	2
**Hydrocodone**		mg	0.33	2
**Oxycodone**		mg	0.5	2
**Pentazocine**		mg	0.12	2
**Nalbuphine**		mg	0.33	5
**Buprenorphine**		mg	25	6
	Sublingual	mg	25	6
	Parenteral	mg	11.1	6
**Butorphanol**		mg	2.33	7
**Tramadol**		mg	0.03	2
**Tapentadol**		mg	0.13	2
**Opium**		mg	0.33	2
**Dihydrocodeine**		mg	0.33	2

**Methadone uses a sliding scale MME conversion based on dosage amount.*

References:

1. https://www.openanesthesia.org/aba_opioid_conversion_-_iv_and_it/
2. CDC_opioid NDC_oralMME_2019
3. Maheshwari_Anesthesiology_2020
4. https://www.cms.gov/Medicare/Prescription-Drug-Coverage/PrescriptionDrugCovContra/Downloads/Oral-MME-CFs-vFeb-2018.pdf

© 2023 Santa Cruz Mercado LA et al. *JAMA Surgery.*

5. https://www.cms.gov/medicare/prescription-drug-coverage/prescriptiondrugcovcontra/downloads/opioid-morphine-eq-conversion-factors-march-2015.pdf
6. Davis_Treating Chronic Pain/ An Overview of Clinical Studies Centered on the Buprenorphine Option_Drugs_2018
7. Mosby, Pharmacology, Chapter 25:"Sedation", 16th edition,2018.
Nagashima H, Karamanian A, Malovany R, et al: Respiratory and circulatory effects of intravenous butorphanol and morphine. Clin Pharmacol Ther 19(6):738–745, 1976.

© 2023 Santa Cruz Mercado LA et al. *JAMA Surgery.*

4. Ideal Body Weight Calculation

Ideal Body Weight Formulas (See References 1-3):

Ideal Body Weight (IBW) shall be calculated using the formula described below.

For male patients: IBW = 50 kg + 2.3 kg x (Height (inches) – 60)
For female Patients: IBW = 45.5 kg + 2.3 kg x (Height (inches) – 60)

Additionally, patients whose actual body weight exceeds their IBW will then have their Adjusted Body Weight (ABW) computed and used as their IBW value.

ABW = IBW + 0.4 x (Actual Body Weight (kg) - IBW)

References:

1. Pai, MP, and Paloucek, FP. "The Origin of the "ideal" Body Weight Equations." The Annals of Pharmacotherapy 34, no. 9 (2000): 1066-069.
2. Devine BJ. “Gentamicin therapy”. Drug Intell Clin Pharm. 1974;8:650–655.
3. Erstad BL. Dosing of medications in morbidly obese patients in the intensive care unit setting. Intensive Care Med 2004; 30:18.

© 2023 Santa Cruz Mercado LA et al. *JAMA Surgery.*

5. **Time stamp definitions**

Multiple variables are considered within the context of certain time windows and events. Below are descriptions of time windows and events, as well an explanation of the programming logic used with collected EDW variables.

a. **Hospital Length of Stay:** The period from admission to discharge.
   Start: HospitalAdmissionDTS
   End: HospitalDischargeDTS

b. **Pre-Operative:** Occurring prior to the start of surgery but on the day of surgery.
   Start: HospitalAdmissionDTS, CPC/PACU admission
   End: AnesthesiaStartDTS, AnesthesiaStart, or Start Data Collection

c. **Patient In Room:** This timestamp was used exclusively to compute pre-emptive analgesia defined as any analgesic treatment given before induction. Due to variability in use of intubation and induction variables, arrival to operating room was used instead
   End: PatientInRoomDTS, In Room

d. **Surgical Duration**: The period of active surgery.
   Start: ProcedureStartDTS
   End: ProcedureCompletedDTS

e. **Intra-operative Anesthesia Care:** The intra-operative period during which opioid drugs are given to treat intra-operative nociception and as prophylaxis for postoperative pain. This period will be used as the *"Primary Exposure Window"*.
   Start: ProcedureStartDTS (T_1 in Figure 3 of the SAP)
   End: Extubation or Stop of CO2 Ventilator + 10 minutes

f. **Anesthesia Maintenance:** The period during which sedative/hypnotic doses are administered in the operating room.
   Start: Time of first sedative or hypnotic administration.
   End: Time at which last sedative or hypnotic is discontinued.

g. **Post Emergence Opioid Administration:** The interval after the end of exposure and arrival to PACU in which opioids may be administered. We used this time window to compute MME of opioids administered to be added to the total PACU MME
   Start: Extubation or Stop of CO2 Ventilator + 10 minutes
   End: InPhase1 or In PACU

h. **PACU Hold:** In the event that patients have completed their surgery but are unable to be transferred to the PACU, it is possible that post-operative treatment can begin while still in the operating room. To consider this, the PACU Hold window is defined.
   Start: Extubation or Stop of CO2 Ventilator
   End: InPhase1 or In PACU

i. **PACU Stay:** This is the period which patients are receiving initial post-operative care. This is also the time window in which we evaluated our primary outcomes.
   Start: InPhase1 or In PACU
   End: Ready for PACU Discharge (see below)

j. **PACU Stay for primary outcomes:** This is the period which patients are receiving initial post-operative care.
   Start: Extubation or Stop of CO2 Ventilator + 10 minutes
   End: Ready for PACU Discharge (see below)

k. **Ready For PACU Discharge:** The end of the PACU Stay time window. Ambulatory and Inpatient cases have different considerations for what denotes the end of their PACU stay. This is defined as follows:

Ambulatory:

- If only 1 Anesthesia Postprocedure Evaluation (APE) Note exists that is associated with a post-operative provider and the timestamp of the note is at least 1 hour from the start of PACU, we used this timestamp.

© 2023 Santa Cruz Mercado LA et al. *JAMA Surgery.*

- If only 1 APE Note exists that is associated with the post-operative provider and the timestamp of the note is less than 1 hour from the start of PACU, we calculated the duration from the start of the PACU stay to the Phase II Care Complete and APE Note timestamp, and we chose the larger duration's timestamp.
- If multiple APE Notes exist, we ignored the first note. Then we used the timestamp of the first note that is associated with a post-operative provider.
- If there are no APE Notes available on record or none of the above criteria apply, we used Phase II Care Complete.

Inpatient:

- If only 1 APE Note exists that is associated with a post-operative provider and the timestamp of the note is at least 1 hour from the start of PACU, we used this timestamp.
- If only 1 APE Note exists that is associated with the post-operative provider and the timestamp of the note is less than 1 hour from the start of PACU, we calculated the duration from the start of the PACU stay to the Phase I Care Complete and APE Note timestamp and chose the larger duration's timestamp.
- If multiple APE Notes exist, we ignored the first note. Then we used the timestamp of the first note that is associated with a post-operative provider.
- If there are no APE Notes available on record or none of the above criteria apply, we used Phase I Care Complete.

l. 24-Hours Post-operatively: This event is defined as 24 hours from the start of the PACU stay time window.
Start: InPhase1 or In PACU
End: Start + 24 hours

m. In-Hospital Post-operatively: This apply only for inpatients, and corresponds to the period of time between the arrival to the floor and discharge from the hospital.
Start: Ready for PACU Discharge
End: HospitalDischargeDTS

6. **ICD code definitions**
Certain variables are defined using the presence or absence of International Classification of Diseases (ICD) codes in the patient records. Below, ICD code information for these variables are provided. Unless otherwise specified, ICD-10 codes were used (see Reference 1).

Variable	Associated ICD codes
Tobacco Use History	Z72.0, Z87.891, F17
Alcohol Use History	F10
Non-Opioid Substance Abuse History:	F12, F13, F14, F15, F16, F18, F19, F55
Opioid Abuse History:	F11
Chronic pain History	G89.4, G89.2
Anxiety History	F41, F42
Depression History	F33
Other Psychiatric History	F04, F07-F09, F20, F21, F23-F32, F34-F40, F43-F54, F56-F79, F81-F83, F85-F99
Neurological History	I60-I69, G00-G09, G11-G19, G22-G29, G31-G88, G90-G99
Dialysis History	Z99.2, Z49, Z94.0
Other Kidney Disorder History	P96.0, N14, R39.2, O00-O08, T79.5, O90.4, N99.0
Chronic Renal Failure History	N18, N19
Acute Renal Failure History	N17
Liver Disease History	K70-K77
Cancer History	C00-C99, C7A, C7B, D00-D49, D3A
DM2 History	E08, E09, O24.4, E13, E11
Rheumatological History	M00-M99
Opioid Allergy History	Z88.5, Z88.6
OSA History	G47.3
Pulmonary Disease History	J00-J99
Difficult Intubation History	T88.4
Hematological History	D65-D69, R79
Chronic Steroid Use History	Z79.5
Systemic Sepsis History	R65.2, R65.1, A41, A42.7, A22.7, B37.7, A26.7, A28.2, A54.86, B00.7, A32.7, A24.1, A39.2, A39.3, A39.4, A20.7, A21.7, A48.3
PONV History	K91.0, R11*<br>* To validate these entries, the database was searched specifically for tags of post-operative nausea and vomiting
Communication Impairment History	H91,3, R47, I69.02, I69.12, I69.82, I69.92, F84, F80, I69.328, F84, I69.22, R41.841
Dementia History	G31.09, F03, F01, G31.83, F18.97, F02, F10.97, F19.97, F13.97, F19.27, A50.45, G10, G21, G30, G31.83, G90.3, A52.19, F22, R41.0, F84.2, G31.01, G20, A81.00, A81.09, Z91.83, F06
Delirium History	F05
Mild Cognitive Impairment History	G31.84
Other Pain Diagnosis History	G89.0, G89.1, G89.3
Cardiac History	I20-I25, I60-I69, I11.0, I42.0, I42.1, I50, N03, N04, N17-N19, R34, I12, I13, Z99.2
Surgical Complication History	T80-T88
**Frailty Index:** The Frailty Index is adapted from the frailty index established in Hope et al. 2015 (see Reference 2). 12 categories categorizing frailty were defined, and if a patient has an ICD code on file that matches within the category, a point is added to the index.	
Alzheimer's, Dementia, Senility:	F03, F05, F01, G30, G20, G21, R41
Delirium:	F05
Parkinson's Disease	G20, G21
Pathologic fracture	M84

© 2023 Santa Cruz Mercado LA et al. *JAMA Surgery.*

Urinary incontinence	R39.81
Dehydration	E86.0
Debility	R53
Protein-calorie malnutrition, kwashiorkor, nutritional marasmus	E40, E41, E43, E45, E46
Accidental falls	W01, W03, W05-W19, V00.3
gait abnormality or lack of coordination	R26, R27
pressure ulcers	L89
abnormal loss of weight or underweight or failure to thrive	R63.4, R63.6, R62.7
**Comorbidity Index:** The Comorbidity Index adapts the Elixhauser Comorbidity Index (see Reference 3). Thirty-one categories were defined, and if a patient has an ICD code on file that matches within the category, a point is added to the index.	
Congestive Heart Failure	I09.9, I11.0, I13.0, I13.2, I25.5, I42.0, I42.5-I42.9, I43, I50, P29.0
Cardiac Arrhythmia	I44.1-I44.3, I45.6, I45.9, I47-I49, R00.0, R00.1, R00.8, T82.1, Z45.0, Z95.0
Valvular Disease	A52.0, I05-I08, I09.1, I09.8, I34-I39, Q23.0-Q23.3, Z95.2-Z95.4
Pulmonary Circulation Disorders	I26, I27, I28.0, I28.8, I28.9
Peripheral Vascular Disorders	I70, I71, I73.1, I73.8, I73.9, I77.1, I79.0, I79.2, K55.1, K55.8, K55.9, Z95.8, Z95.9
Hypertension without Complications	I10
Hypertension with Complications	I11-I13, I15
Paralysis	G04.1, G11.4, G80.1, G80.2, G81, G82, G83.0-G83.4, G83.9
Other Neurological Disorders	G10-G13, G20-G22, G25.4, G25.5, G31.2, G31.8, G31.9, G32, G35-G37, G40, G41, G93.1, G93.4, R47.0, R56
Chronic Pulmonary Disease	I27.8, I27.9, J40-J47, J60-J67, J68.4, J70.1, J70.3
Diabetes without Complications	E10.0, E10.1, E10.9, E11.0, E11.1, E11.9, E12.0, E12.1, E12.9, E13.0, E13.1, E13.9, E14.0, E14.1, E14.9
Diabetes with Complications	E10.2-E10.8, E11.2-E11.8, E12.2-E12.8, E13.2-E13.8, E14.2-E14.8
Hypothyroidism	E00-E03, E89.0
Renal Failure	I12.0, I13.1, N18, N19, N25.0, Z49.0-Z49.2, Z94.0, Z99.2
Liver Disease	B18, I85, I86.4, I98.2, K70, K71.1, K71.3-K71.5, K71.7, K72-K74, K76.0, K76.2-K76.9, Z94.4
Peptic Ulcer Disease excluding Bleeding	K25.7, K25.9, K26.7, K26.9, K27.7, K27.9, K28.7, K28.9
HIV/AIDS	B20-B22, B24
Lymphoma	C81-C85, C88, C96, C90.0, C90.2
Metastatic Cancer	C77-C80
Solid Tumor without Metastasis	C00-C26, C30-C34, C37-C41, C43, C45-C58, C60-C76, C97
Rheumatoid Arthritis/Collagen	L94.0, L94.1, L94.3, M05, M06, M08, M12.0, M12.3, M30, M31.0-M31.3, M32-M35, M45, M46.1, M46.8, M46.9
Coagulopathy	D65-D68, D69.1, D69.3-D69.6
Obesity	E66
Weight Loss	E40-E46, R63.4, R64
Fluid and Electrolyte Disorders	E22.2, E86, E87
Blood Loss Anemia	D50.0

© 2023 Santa Cruz Mercado LA et al. *JAMA Surgery.*

Deficiency Anemia	D50.8, D50.9, D51-D53
Alcohol Abuse	E52, F10, G62.1, I42.6, K29.2, K70.0, K70.3, K70.9, T51, Z50.2, Z71.4, Z72.1
Drug Abuse	F11 E52, F16, F18, F19, Z71.5, Z72.2
Psychoses	F20, F22-F25, F28, F29, F30.2, F31.2, F31.5
Depression	F20.4, F31.3-F31.5, F32, F33, F34.1, F41.2, F43.2
Conversion	
Laparoscopic surgical procedure converted to open procedure	V64.41
Code for Procedure converted to open procedure	Z53.3
Laparoscopic procedure converted to open	Z53.31
Arthroscopic procedure converted to open	Z53.33
Arthroscopic procedure converted to open	Z53.39

Postop Opioid Complications: To confirm opioid complications, along with the ICD definition as described above in PONV Hx, postoperative diagnoses were identified by filtering for the following in the diagnosis title: Postoperative nausea and vomiting (PONV) in PACU, Postoperative nausea and vomiting (PONV) in hospital, Postoperative shivering, Pruritus, Ileus, Respiratory depression, Unplanned intubation, Aspiration, Pneumonia, Ileus, Constipation, Sedation, and Urinary retention.

3 Month/12 Month Postoperative Chronic Pain Diagnosis: To confirm chronic pain diagnosis postoperatively, along with the ICD definition as described above for Chronic Pain Hx, postoperative diagnoses were identified by filtering for "chronic pain" in the diagnosis title.

References:

1. https://www.aapc.com/codes/icd-10-codes
2. https://agsjournals.onlinelibrary.wiley.com/doi/10.1111/jgs.13436
3. http://mchp-appserv.cpe.umanitoba.ca/viewConcept.php?printer=Y&conceptID=1436
4. https://icd10cmtool.cdc.gov/?fy=FY2022&query=converted%20to%20open

© 2023 Santa Cruz Mercado LA et al. *JAMA Surgery.*

C. Assessment of Minimal Clinically Important Difference (MCID)

Following previous work and published viewpoints established for clinical trials in the anesthesiology and pain management literature, we defined the MCID for maximum post-operative pain to be a 1-point change on the numerical rating scale (NRS) for pain (Maheshwari 2020, Olsen 2017, Myles 2017). Similarly, we defined the MCID for opioid administration as a 20% change in MME (Kehlet 2005, Goudman 2020, Nowak 2020). In order to evaluate this MCID over an exposure range representative of the observed data, we chose to assess the change in these outcomes over a counterfactual 2 standard deviation increase in each exposure. As shown in **Tables C1 and C2**, the change in MME for a 2 standard deviation increase in both Fentanyl or Hydromorphone is statistically indistinguishable from the pre-defined 20% MCID value, since the 95% confidence intervals in both cases overlap the 20% MCID value. Meanwhile, the magnitude of the change in maximum post-operative pain appeared significantly lower than the pre-defined 1-point NRS value (i.e., 95% confidence intervals for the change in the maximum post-operative pain spanned a range less than 1-point in magnitude).

We also evaluated secondary outcomes for pain and MME over 24 hour and in hospital intervals, respectively, with respect to our MCID criteria (**Table C3**). Similar to the primary outcomes, the change in total MME at 24 hours and in hospital after a 2 standard deviation increase in Fentanyl exposure were indistinguishable from the pre-defined 20% MCID value. Similarly, the change in total MME at 24 hours after a 2 standard deviation increase in Hydromorphone exposure was indistinguishable from the pre-defined 20% MCID value. The magnitude of change for the remaining secondary outcomes (max pain at 24 hours and in hospital for both drugs, total MME in hospital for Hydromorphone) was significantly lower than the respective pre-defined MCID values.

**Predicted change in outcome**	**+2SD Fentanyl**	**+2SD Hydromorphone**
Max Pain in PACU (NRS)	-0.24 (-0.38, -0.01)	-0.20 (-0.33, -0.01)
Total MME in PACU (%)	-15.4% (-30.3%, -0.0%)	-15.3% (-25.0%, -9.1%)

Table C1. Changes in pain and MME outcomes after a 2 standard deviations increase in Fentanyl or Hydromorphone exposure under the model employing ICD + CPT codes in the propensity weighting.

**Predicted change in outcome**	**+2SD Fentanyl**	**+2SD Hydromorphone**
Max Pain in PACU (NRS)	-0.20 (-0.32, -0.01)	-0.23 (-0.38, -0.01)
Total MME in PACU (%)	-12.5% (-25.8%, -0.0%)	-24.4% (-44.4%, -12.1%)

Table C2. Changes in pain and MME outcomes after a 2 standard deviations increase in Fentanyl or Hydromorphone exposure under the model without ICD + CPT codes in the propensity weighting.

**Predicted change in outcome**	**+2SD Fentanyl**	**+2SD Hydromorphone**
Max Pain at 24 h (NRS)	0.11 (0.04, 0.17)	0.02 (-0.03, 0.07)
Max Pain in hospital (NRS)	0.05 (0.01, 0.10)	0.27 (0.18, 0.34)
Total MME at 24 h (%)	-28.0% (-68.8%, -0.0%)	-30.0% (-61.1%, -0.0%)
Total MME in hospital (%)	-13.2% (-26.1%, -0.0%)	-3.8% (-14.5%, -0.0%)

Table C3. Changes in secondary outcomes for pain and MME at 24 h and in hospital after a 2 standard deviation increase in Fentanyl or Hydromorphone exposure under the model employing ICD + CPT codes in the propensity weighting.

References:

- Maheshwari, Kamal, Avitsian, Rafi, Sessler, Daniel I, Makarova, Natalya, Tanios, Marianne, Raza, Syed, Traul, David, Rajan, Shobana, Manlapaz, Mariel, Machado, Sandra, Krishnaney, Ajit, Machado, Andre, Rosenquist, Richard, and Kurz, Andrea. "Multimodal Analgesic Regimen for Spine Surgery." Anesthesiology (Philadelphia) 132, no. 5 (2020): 992-1002
- Olsen, Mette Frahm, Bjerre, Eik, Hansen, Maria Damkjaer, Hilden, Jorgen, Landler, Nino Emanuel, Tendal, Britta, and Hrobjartsson, Asbjorn. "Pain Relief That Matters to Patients: Systematic Review of Empirical Studies Assessing the Minimum Clinically Important Difference in Acute Pain." BMC Medicine 15, no. 1 (2017): 35.
- Myles, P.S, Myles, D.B, Galagher, W, Boyd, D, Chew, C, MacDonald, N, and Dennis, A. "Measuring Acute Post-operative Pain Using the Visual Analog Scale: The Minimal Clinically Important Difference and Patient Acceptable Symptom State." British Journal of Anaesthesia : BJA 118, no. 3 (2017): 424-29
- Kehlet, H. "Post-operative Opioid Sparing to Hasten Recovery - What Are the Issues?" Anesthesiology (Philadelphia) 102, no. 6 (2005): 1083-085.

© 2023 Santa Cruz Mercado LA et al. *JAMA Surgery.*

- Goudman, Lisa, De Smedt, Ann, Forget, Patrice, and Moens, Maarten. "Determining the Minimal Clinical Important Difference for Medication Quantification Scale III and Morphine Milligram Equivalents in Patients with Failed Back Surgery Syndrome." Journal of Clinical Medicine 9, no. 11 (2020): 3747.
- Nowak, Hartmuth, Zech, Nina, Asmussen, Sven, Rahmel, Tim, Tryba, Michael, Oprea, Guenther, Grause, Lisa, Schork, Karin, Moeller, Manuela, Loeser, Johannes, Gyarmati, Katharina, Mittler, Corinna, Saller, Thomas, Zagler, Alexandra, Lutz, Katrin, Adamzik, Michael, and Hansen, Ernil. "Effect of Therapeutic Suggestions during General Anaesthesia on Post-operative Pain and Opioid Use: Multicentre Randomised Controlled Trial." BMJ 371 (2020): M4284.

D. Data Verification Procedures

Data were initially acquired through the EDW database using SQL queries designed to find information pertinent to the variables listed above. Queries were built to draw from records that could help characterize a variable directly (e.g., height and weight information) or indirectly (e.g., reviewing ICD records to determine the presence or absence of specific diagnoses). Once the information was acquired, Matlab was used to both evaluate and process the data. For some variables, information was directly incorporated without additional processing (e.g., height information directly documented in the medical record). Other variables were calculated using the relevant records (e.g., MME values were calculated from the drug administration records). Code reviews were performed regularly throughout the project (RL, KMB, JJ, RG, PD, TADS).

After the first pass of processing, the data distribution for individual variables were analyzed using R and Matlab. Unusual skews, multimodal behavior, or significant missing data was reviewed and acknowledged by physicians familiar with perioperative care (LSC, RG, GB, EB), and each previous step of the data acquisition and processing pipeline was verified to ensure accuracy. In addition, for each variable, a random sampling of at least ten cases was selected, and EPIC records were manually reviewed by said physicians and compared against the processed data to ensure accuracy of information. Variance inflation factors were calculated between all variables to ensure that variables were sufficiently uncorrelated for subsequent analyses.

© 2023 Santa Cruz Mercado LA et al. *JAMA Surgery.*

E. Pk/Pd Model

Below are tables containing information for Pk/PD models for exposure drugs.

Volume Compartments
V1: Central Compartment Volume
V2: Fast Compartment Volume (vessel-rich group)
V3: Slow Compartment Volume (vessel-poor group)

Drug	V1	V2	V3
**Fentanyl[1,2]**	12.1 x weight/70	35.7 x weight/70	224 L x weight/70
**Hydromorphone[1,3]**	0.16 x weight	V1 x k12/k21	V1 x k13/k31

Clearances:
CL1: Elimination Clearance
CL2: Clearance Between V1 and V2
CL3: Clearance Between V1 and V3

Drug	CL1	CL2	CL3
**Fentanyl[1,2]**	0.632 x (weight/70)^0.75	2.8 x (weight/70)^0.75	1.55 x (weight/70)^0.75
**Hydromorphone[1,3]**	V1 x k10	V1 x k12	V1 x k13

Rate Constants
k10: Elimination Rate Constant
ke0: Equilibrium Rate Constant (Between blood and effect site)
k12, k21: Rate Constants Between V1 and V2
k13, k31: Rate Constants Between V1 and V3

Drug	k10	k12	k13	k21	k31	Ke0
**Fentanyl[1,2]**	CL1/V1	CL2/V1	CL3/V1	CL2/V2	CL3/V3	0.203282577
**Hydromorphone[1,3]**	0.116	0.3	0.08	0.03	0.00095	0.014996316

References:
1. McClain and Hug, Clin Pharmacol Ther 1980
2. Shafer et al, Anesthesiology 1990
3. Drover et al, Anesthesiology 2002

© 2023 Santa Cruz Mercado LA et al. *JAMA Surgery.*

F. Adjustment for Bias and Confounding

The following diagram represents the general structure of the primary model design for this study.

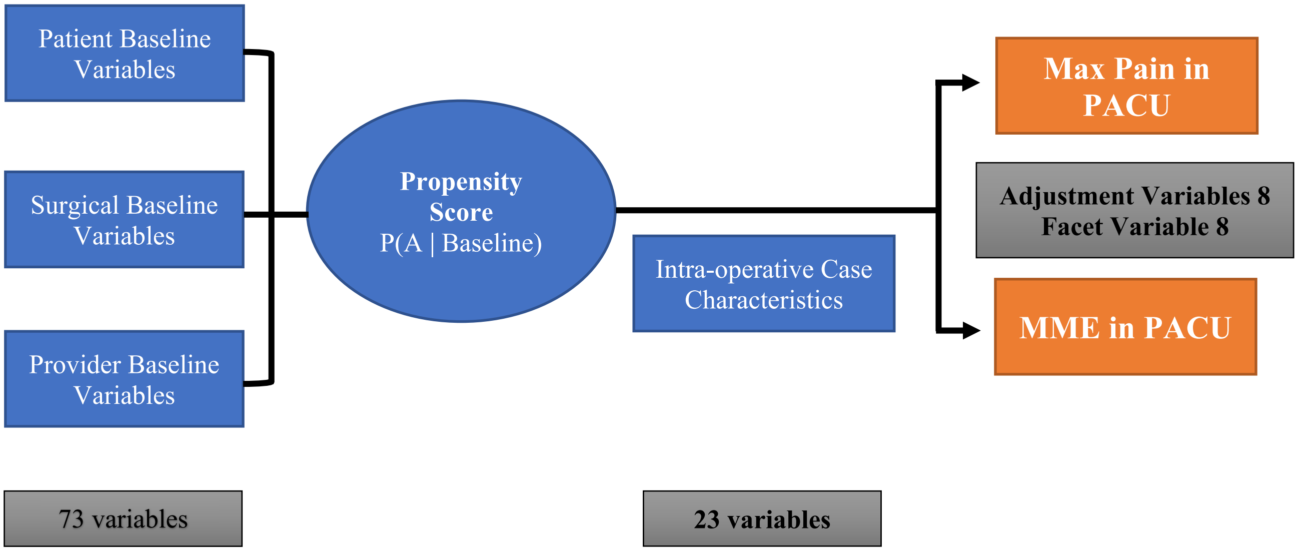

1. **Direct Acyclic Graph (DAG) Analysis**
   A direct acyclic graph (DAG) of potential relationships between the studied variables was created using the Dagity web-tool (http://www.dagitty.net/dags.html). A visualization of direct relationships between variables is presented in Figure XXX. An interactive version showcasing specific variable interactions is accessible online (https://observablehq.com/d/a4b50f86523921b5), and variable definitions can be found above.

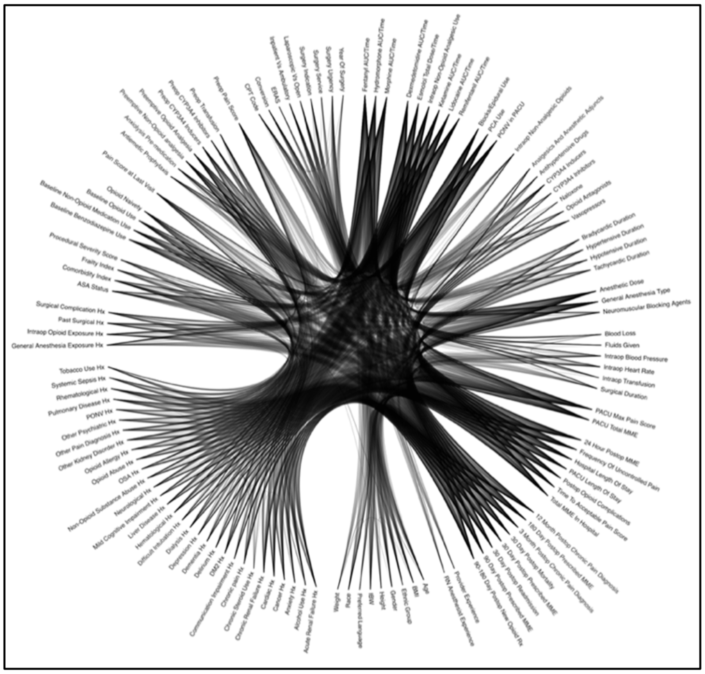

2. **Baseline variables:** These variables were used to construct the propensity weights:

Patient Variables	Surgical Variables	Provider Variables

© 2023 Santa Cruz Mercado LA et al. *JAMA Surgery*.

Age	Other Psychiatric Hx	Surgical Complication Hx	Provider Experience
Gender	Neurological Hx	Past Surgical Hx	RN Anesthetist Experience
Race	Dialysis	Intraoperative Opioid Exposure Hx	
Ethnic Group	Other Kidney Disorder Hx	General Anesthesia Exposure Hx	
Preferred Language	Acute Renal Failure	Procedural Severity Score	
BMI	Chronic Renal Failure Hx	Surgery Urgency	
Height	Liver Disease Hx	Inpatient vs Ambulatory	
Weight	Cancer Hx	Laparoscopic vs Open	
IBW	DM2 Hx	Surgery Indication	
Tobacco Use Hx	Rheumatological Hx	CPT Code	
Alcohol Use Hx	Opioid Allergy Hx	Surgery Service	
Non-Opioid Substance Abuse Hx	OSA Hx	ERAS	
Opioid Abuse Hx	Pulmonary Disease Hx	Year of Surgery	
Chronic Pain Hx	Difficult Intubation Hx		
Anxiety Hx	Hematological Hx		
Depression Hx	Chronic Steroid use		
PONV Hx	Systemic Sepsis		
Communication Impairment	Dementia Hx		
Delirium Hx	Mild Cognitive Impairment Hx		
Other Pain Diagnosis	Cardiac Hx		
Frailty Index	Comorbidity Index		
ASA status	Baseline Opioid Use		
Baseline Non-Opioid Medication use	Baseline benzodiazepine use		
Baseline Opioid Antagonists Use	Pain Score at last visit		
Blood Pressure at last visit	Heart Rate at last Visit		
Anxiolysis Pre-medication	Preoperative Transfusion		
Preemptive Non-Opioid Analgesia	Preemptive Opioid Analgesia		
Preop CYP3A4 Inducers	Preop CYP3A4 Inhibitors		
Preop Pain Score			

Hx: history

© 2023 Santa Cruz Mercado LA et al. *JAMA Surgery.*

3. **Intraoperative Case Characteristics:** These variables were used as adjustment covariates in our models to account for additional confounding and to increase model precision. Since these variables represent intraoperative events occurring concurrently with the exposures, they were employed as covariates rather than components of the propensity score. These variables were not controlled for confounding, and thus were not interpreted.
    a. Surgical Duration
    b. Conversion
    c. Vasopressors
    d. CYP3A4 Inducers
    e. CYP3A4 Inhibitors
    f. Antihypertensive Drugs
    g. Naloxone
    h. Hypotensive Duration
    i. Hypertensive Duration
    j. Tachycardic Duration
    k. Bradycardic Duration
    l. Intraoperative Blood Pressure
    m. Intraoperative Heart Rate
    n. General Anesthesia Type
    o. Anesthetic Dose
    p. Neuromuscular Blocking Agents
    q. Fluids Given
    r. Blood Loss
    s. Intraoperative Transfusion
    t. Difficult airway
    u. Inpatient vs. Ambulatory
    v. Analgesics and Anesthetic Adjuncts
    w. Intraoperative Non-Analgesic Opioids

© 2023 Santa Cruz Mercado LA et al. *JAMA Surgery*.

4. **Additional adjustment variables**

In addition to exposures and intraoperative case characteristic variables, measurement facets and additional adjustment variables were included as covariates in order to adjust for potential confounding and to improve model precision. Since these variables represent post-operative events or circumstances they were employed as covariates rather than components of the propensity weight. These variables were not controlled for confounding, and thus were not interpreted. A full list of variables and fitted coefficients for each model are reported in **Supplemental Table 1**.

Measurement facet variables were employed to account for variance introduced by rater observations in our outcome measures of pain. These include:

- Location of postoperative care (where the pain measurement was obtained): PACU, CPC, or other
- Time of day that the record was obtained
- Day of week that the record was obtained
- Pain score record type: Defined as either numeric (using the 0-10 NRS pain scale) or a verbal report ("string") that was translated to the NRS equivalent (No/Denies Pain = 0, Mild Pain = 2, Moderate Pain = 4, Severe Pain = 6, Very Severe Pain = 8, Worst Pain Possible = 10).

Adjustment variables were added to account for the influence of opioids administered after the exposure window:

- Effect site concentration at the first time of recorded max pain score for fentanyl (PACU and 24h postop max pain only)
- Effect site concentration at the first time of recorded max pain score for hydromorphone (PACU and 24h postop max pain only)
- Cumulative MME for opioids (except for hydromorphone and fentanyl) given between the start of the exposure window (extubation + 10 minutes) and the first time of recorded max pain score (PACU and 24h postop max pain) or hospital discharge (In-hospital max pain).
- Postoperative non-opioid analgesic usage (ketorolac, acetaminophen, gabapentin)
- Hurdle models of opioid usage (PACU total MME, 24h Postop MME, In-Hospital total MME):
- Location of postoperative care: PACU, CPC, or other
- Time of day of midpoint of PACU stay
- Day of week of midpoint of PACU stay
- Effect site concentration at time of PACU admission for fentanyl (PACU total MME only)
- Effect site concentration at time of PACU admission for hydromorphone (PACU total MME only)
- Mean postop effect site concentration for fentanyl (24h postop and In-hospital total MME only)
- Mean postop effect site concentration for hydromorphone (24h postop and In-hospital total MME only)
- Postoperative non-opioid analgesic usage (ketorolac, acetaminophen, gabapentin)
- Postoperative nausea and vomiting (PONV) in PACU
- Non-analgesic opioids administered in PACU

Cox proportional hazards models for time-to-event outcomes (Time to recovery from sedation, PACU length of stay, Hospital length of stay) employed the following set of adjustment variables:

- Surgery type (ambulatory/inpatient)
- Location of postoperative care: PACU, CPC, or other
- Effect site concentration at time of PACU admission for fentanyl (Recovery from sedation only)
- Effect site concentration at time of PACU admission for hydromorphone (Recovery from sedation only)
- Non-analgesic opioids administered in PACU
- Postoperative non-opioid analgesic usage (ketorolac, acetaminophen, gabapentin)

Logistic regression models for binary outcomes (Frequency of uncontrolled pain, 3-, 12-month chronic pain, 30-, 90-, 180-day refill, persistent use, PONV in PACU, respiratory depression, 30-day postoperative mortality, 30-day postoperative readmission) employed the following set of adjustment variables:

- Surgery type (ambulatory/inpatient)
- Location of postoperative care: PACU, CPC, or other
- Non-analgesic opioids administered in PACU
- Pain score record type (uncontrolled pain only)
- Time of day of max pain score record (frequency of uncontrolled pain only)
- Day of week of max pain score record (frequency of uncontrolled pain only)

© 2023 Santa Cruz Mercado LA et al. *JAMA Surgery.*

- Effect site concentration at time of PACU admission for fentanyl (PONV, respiratory depression only)
- Effect site concentration at time of PACU admission for hydromorphone (PONV, respiratory depression only)
- Postoperative nausea and vomiting in PACU (30-, 90-, 180-day refill, 30-day postoperative mortality, 30-day postoperative readmission, persistent use)
- Cumulative MME for opioids (except for hydromorphone and fentanyl) given between the start of the exposure window (extubation + 10 minutes) and 24 hours postoperative (frequency of uncontrolled pain) or hospital discharge (3-, 12-month chronic pain, 30-day postoperative mortality, 30-day postoperative readmission).
- Postoperative non-opioid analgesic usage (ketorolac, acetaminophen, gabapentin)

© 2023 Santa Cruz Mercado LA et al. *JAMA Surgery.*

5. **Propensity Adjustment:**

First, we construct univariate propensity weights as the ratio between the marginal density of each exposure variable, and the conditional density of each exposure variable, given the values of the confounding variables.

Let x denote the exposure for a given surgical case, and c denote the confounding variables. $w(x)$ is then the propensity weight assigned to that case.

$$w(x,c) = \frac{f(x)}{f(x|c)}$$

We model the marginal distribution of x as a mixed random variable, which is 0 with some probability equal to the proportion of zero exposures, and if nonzero, normally distributed according to the maximum likelihood estimate of the population mean and variance of nonzero exposures.

Let z be an indicator for $x > 0$.

$$w(x,z,c) = \frac{f(x,z)}{f(x,z|c)} = \frac{f(x|z)f(z)}{f(x|z,c)f(z|c)}$$

$$f(z) = \begin{cases} p(x > 0) : z = 1 \\ p(x = 0) : z = 0 \end{cases}$$

$$f(x|z) = \begin{cases} f_{N\left(\hat{\mu}_{x[z=1]}, \hat{\sigma}^2_{x[z=1]}\right)}(x) & : z = 1 \\ \delta(x) & : z = 0 \end{cases}$$

We model the conditional distribution of x also as a mixed random variable. The conditional probability of $x > 0$ is predicted using XGBoost[1], a gradient boosting machine learning model, and if nonzero, $x|c$ is modeled as normally distributed, with mean predicted by a separate XGBoost model, and variance equal to the residual variance of model predictions.

Let $m(c)$ denote the model prediction of x, given the value of confounding variables c.

$$f(x|z,c) = \begin{cases} f_{N\left(m(c), \hat{\sigma}^2_{x-m(c)}\right)}(x) & : z = 1 \\ \delta(x) & : z = 0 \end{cases}$$

$$w(x,z,c) = \begin{cases} \dfrac{f_{N\left(\hat{\mu}_{x[z=1]}, \hat{\sigma}^2_{x[z=1]}\right)}(x)p(x > 0)}{f_{N\left(m(c), \hat{\sigma}^2_{x-m(c)}\right)}(x)p(x > 0|c)} & : z = 1 \\ \dfrac{p(x = 0)}{p(x = 0|c)} & : z = 0 \end{cases}$$

As we find that the distributions for intraoperative fentanyl and hydromorphone administration are approximately independent, we construct our multivariate propensity weights as the product of the univariate propensity weights for intraoperative fentanyl and hydromorphone.

© 2023 Santa Cruz Mercado LA et al. *JAMA Surgery.*

G. Counterfactual Estimates

Counterfactual predictions were computed as the mean difference in model-adjusted predictions at the observed values in our dataset before propensity weighting, and at the observed values with the addition of 100 mcg fentanyl or 500 mcg hydromorphone. We estimated the average effect that these additions in intraoperative opioid administration would produce in mean effect site concentration through regression analysis between effect site concentrations and intraoperative opioid dosages. This yielded ratios of 151.58 mcg fentanyl to 1 ng/mL, and 410.76 mcg hydromorphone to 1 ng/mL.

Confidence intervals were computed by bootstrapping from the multivariate gaussian distribution of each model's parameters, with mean equal to the model's parameter estimates, and covariance equal to the model covariance matrix. While the parameters of the binomial and log-normal components of the hurdle models are not independent, we sample from their parameter distributions independently for ease of computation, which yields an upper bound to the width of the computed confidence interval.

© 2023 Santa Cruz Mercado LA et al. *JAMA Surgery.*

H. Pain Management in PACU

At MGH we follow ASPAN Guidelines and MGH Policy to guide analgesics in the PACU. These guidelines calls for the use of a developmentally appropriate pain scale and to select the type of analgesics based on the pain intensity. Even though we do not have set pain level cut offs, patients experiencing mild pain (NRS 1-3) usually receive acetaminophen, patients experiencing moderate pain (NRS 4-6) usually receive Oxycodone and patients with severe pain (NRS > 6) receive fentanyl or hydromorphone, as first and second line respectively. All opioids should have to have pain scale in the order for PRN (as needed) administration.

Reference:

- https://www.aspan.org/Publications-Resources/ASPAN-Publications/ASPAN-Standards

© 2023 Santa Cruz Mercado LA et al. *JAMA Surgery.*

eTable 1: Model coefficients for adjusted model

A. PACU Max Pain Score (primary)

Coefficient	Value	Std. Error
Intraoperative fentanyl exposure	-0.2045	0.0096
Intraoperative hydromorphone exposure	-0.0614	0.0098
Fentanyl effect site concentration at time of max pain	0.4148	0.0110
Hydromorphone effect site concentration at time of max pain	0.6877	0.0232
Pain scale (verbal/numeric)	4.6743	0.0431
Intraoperative non-analgesic opioid usage	-0.0056	0.0916
Intraoperative Vasopressors	0.0248	0.0131
Intraoperative CYP3A4 Inducers	-0.0971	0.0206
Intraoperative CYP3A4 Inhibitors	0.2092	0.0937
Intraoperative Antihypertensive Drugs	-0.0000	0.0271
Naloxone usage	-0.1353	0.1065
Intraoperative hypotensive duration	0.0344	0.0122
Intraoperative hypertensive duration	0.0054	0.0104
Intraoperative tachycardic duration	-0.0219	0.0102
Intraoperative bradycardic duration	-0.0916	0.0127
N2O dosage	0.1047	0.0086
Isoflurane dosage	0.0115	0.0084
Propofol dosage	0.2042	0.0605
Sevoflurane dosage	0.1503	0.0096
Neuromuscular blocking agent usage	0.0794	0.0201
Intraoperative mean blood pressure	0.0762	0.0093
Intraoperative mean heart rate	0.0519	0.0138
Surgical duration	0.0286	0.0145
Intraoperative blood loss	0.0261	0.0098
Intraoperative blood transfusions	-0.1545	0.0628
Surgery type (Ambulatory/Inpatient)	0.9659	0.0223
Intraoperative antiemetic prophylaxis	0.2107	0.0206
Intraoperative ketorolac dosage	-0.0053	0.0083
Intraoperative acetaminophen dosage	0.0450	0.0087
Intraoperative lidocaine dosage	-0.0228	0.0103
Intraoperative ketamine dosage	0.0115	0.0076
Intraoperative dexmedetomidine dosage	0.0182	0.0094
Intraoperative esmolol dosage	-0.0084	0.0030
Intraoperative dexamethasone dosage	0.0180	0.0085
Difficult intubation	-0.1423	0.1121
Conversion	-0.1745	0.2027
Time of day of max pain, 12AM to 4AM	-0.2411	0.0942
Time of day of max pain, 4AM to 8AM	0.1440	0.1173
Time of day of max pain, 8AM to 12PM	0.4187	0.0444
Time of day of max pain, 12PM to 4PM	0.4496	0.0427
Time of day of max pain, 4PM to 8PM	0.2424	0.0432
Day of week of max pain, Sunday	0.3915	0.0825
Day of week of max pain, Monday	0.0385	0.0534
Day of week of max pain, Tuesday	-0.0807	0.0529
Day of week of max pain, Wednesday	0.1221	0.0534
Day of week of max pain, Thursday	-0.1572	0.0541
Day of week of max pain, Friday	-0.0361	0.0530
Postoperative care location: PACU	0.6398	0.0309
Postoperative care location: CPC	-0.7159	0.0265
Postoperative acetaminophen dosage	0.3391	0.0087
Postoperative gabapentin dosage	0.0730	0.0084
Postoperative ketorolac dosage	0.1298	0.0069
Total opioid administration between time of extubation and max pain	1.5223	0.0331

© 2023 Santa Cruz Mercado LA et al. *JAMA Surgery.*

Coefficient	Value	Std. Error
0|1	3.3941	0.0781
1|2	3.5517	0.0783
2|3	4.0794	0.0787
3|4	4.5536	0.0791
4|5	5.1030	0.0794
5|6	5.8562	0.0799
6|7	6.4673	0.0803
7|8	7.1952	0.0808
8|9	8.2431	0.0820
9|10	8.8758	0.0831

© 2023 Santa Cruz Mercado LA et al. *JAMA Surgery.*

B. 24h Postop Max Pain

Coefficient	Value	Std. Error
Intraoperative fentanyl exposure	0.0341	0.0112
Intraoperative hydromorphone exposure	0.0473	0.0096
Pain scale (verbal/numeric)	1.1684	0.0285
Intraoperative non-analgesic opioid usage	-0.1208	0.0905
Intraoperative Vasopressors	-0.0226	0.0131
Intraoperative CYP3A4 Inducers	-0.1822	0.0266
Intraoperative CYP3A4 Inhibitors	-0.1482	0.1079
Intraoperative Antihypertensive Drugs	-0.0046	0.0296
Intraoperative hypotensive duration	-0.0051	0.0127
Intraoperative hypertensive duration	0.0214	0.0109
Intraoperative tachycardic duration	-0.0243	0.0116
Intraoperative bradycardic duration	-0.0416	0.0133
N2O dosage	0.0690	0.0107
Isoflurane dosage	0.0559	0.0109
Propofol dosage	0.0698	0.0748
Sevoflurane dosage	0.1979	0.0129
Neuromuscular blocking agent usage	0.0447	0.0232
Intraoperative mean blood pressure	0.0160	0.0116
Intraoperative mean heart rate	0.0558	0.0172
Surgical duration	0.0547	0.0151
Intraoperative blood loss	0.0297	0.0094
Intraoperative blood transfusions	-0.0664	0.0589
Intraoperative antiemetic prophylaxis	0.3555	0.0246
Intraoperative ketorolac dosage	0.0251	0.0116
Intraoperative acetaminophen dosage	0.0323	0.0093
Intraoperative lidocaine dosage	0.0068	0.0116
Intraoperative ketamine dosage	0.0181	0.0081
Intraoperative dexmedetomidine dosage	0.0315	0.0089
Intraoperative esmolol dosage	0.0043	0.0038
Intraoperative dexamethasone dosage	-0.1010	0.0139
Difficult intubation	0.0257	0.1365
Conversion	0.3546	0.0011
Postoperative ketorolac dosage	0.0132	0.0021
Postoperative acetaminophen dosage	0.0005	0.0000
Postoperative gabapentin dosage	0.0003	0.0001
Postoperative care location: PACU	0.3357	0.0320
Postoperative care location: CPC	0.2991	0.0234
Naloxone usage	0.0427	0.0088
Total opioid administration between time of extubation and PACU admission	0.0994	0.0136
Fentanyl effect site concentration at time of max pain	-0.4290	0.0509
Hydromorphone effect site concentration at time of max pain	0.2850	0.0186
Total opioid administration between PACU admission and 24 hours postop	1.6168	0.0334
0|1	-1.7708	0.0458
1|2	-1.5640	0.0448
2|3	-0.9534	0.0429
3|4	-0.4069	0.0423
4|5	0.1960	0.0422
5|6	0.9828	0.0427
6|7	1.6280	0.0433
7|8	2.3637	0.0441
8|9	3.4498	0.0456
9|10	4.1806	0.0471

© 2023 Santa Cruz Mercado LA et al. *JAMA Surgery.*

C. In-hospital Max Pain

Coefficient	Value	Std. Error
Intraoperative fentanyl exposure	0.0025	0.0096
Intraoperative hydromorphone exposure	0.1543	0.0089
Pain scale (verbal/numeric)	1.4598	0.0281
Intraoperative Vasopressors	-0.0252	0.0131
Intraoperative CYP3A4 Inducers	-0.2247	0.0267
Intraoperative CYP3A4 Inhibitors	-0.1553	0.1072
Intraoperative Antihypertensive Drugs	-0.0569	0.0299
Intraoperative hypotensive duration	-0.0148	0.0127
Intraoperative hypertensive duration	0.0197	0.0108
Intraoperative tachycardic duration	0.0027	0.0120
Intraoperative bradycardic duration	-0.0726	0.0134
N2O dosage	0.1032	0.0108
Isoflurane dosage	0.0830	0.0110
Propofol dosage	0.2840	0.0747
Sevoflurane dosage	0.2481	0.0129
Neuromuscular blocking agent usage	-0.0040	0.0233
Intraoperative mean blood pressure	0.0086	0.0116
Intraoperative mean heart rate	0.0324	0.0173
Surgical duration	0.0322	0.0145
Intraoperative blood loss	0.0411	0.0096
Intraoperative blood transfusions	-0.0807	0.0576
Intraoperative antiemetic prophylaxis	0.3866	0.0246
Intraoperative ketorolac dosage	0.0290	0.0116
Intraoperative acetaminophen dosage	0.0302	0.0094
Intraoperative lidocaine dosage	0.0197	0.0112
Intraoperative ketamine dosage	0.0641	0.0079
Intraoperative dexmedetomidine dosage	0.0448	0.0089
Intraoperative esmolol dosage	0.0064	0.0038
Intraoperative dexamethasone dosage	-0.1389	0.0139
Difficult intubation	0.0034	0.1370
Conversion	0.2764	0.0011
Postoperative ketorolac dosage	0.0120	0.0020
Postoperative acetaminophen dosage	0.0005	0.0000
Postoperative gabapentin dosage	0.0009	0.0001
Postoperative care location: PACU	0.5384	0.0318
Postoperative care location: CPC	0.3462	0.0235
Naloxone usage	0.0323	0.0085
Total opioid administration between time of extubation and PACU admission	0.0831	0.0136
Total opioid administration between PACU admission and hospital discharge	1.0451	0.0564
0|1	-1.6446	0.0374
1|2	-1.4408	0.0360
2|3	-0.8426	0.0335
3|4	-0.3141	0.0326
4|5	0.2591	0.0324
5|6	0.9912	0.0329
6|7	1.5799	0.0335
7|8	2.2435	0.0344
8|9	3.2194	0.0362
9|10	3.8832	0.0379

© 2023 Santa Cruz Mercado LA et al. *JAMA Surgery.*

D. PACU total MME (primary) binomial

Coefficient	Value	Std. Error
(Intercept)	0.2095	3.6354
Intraoperative fentanyl exposure	-0.0672	0.0124
Intraoperative hydromorphone exposure	-0.2087	0.0174
Fentanyl effect site concentration at PACU admission	0.2202	0.0146
Hydromorphone effect site concentration at PACU admission	0.3367	0.0173
Intraoperative non-analgesic opioid usage	0.5657	0.3751
PACU non-analgesic opioid usage	5.5425	30.2755
Intraoperative Vasopressors	0.0614	0.0193
Intraoperative CYP3A4 Inducers	-0.1890	0.0236
Intraoperative CYP3A4 Inhibitors	0.4193	0.1219
Intraoperative Antihypertensive Drugs	0.0416	0.0326
Naloxone usage	-1.5156	0.1310
Intraoperative hypotensive duration	-0.0182	0.0142
Intraoperative hypertensive duration	-0.0542	0.0121
Intraoperative tachycardic duration	-0.0261	0.0124
Intraoperative bradycardic duration	-0.0943	0.0143
N2O dosage	0.0212	0.0096
Desflurane dosage	0.1046	0.0119
Isoflurane dosage	0.1076	0.0110
Propofol dosage	0.3474	0.0676
Sevoflurane dosage	0.2411	0.0115
Neuromuscular blocking agent usage	0.1400	0.0235
Intraoperative mean blood pressure	0.1622	0.0106
Intraoperative mean heart rate	0.0296	0.0152
Surgical duration	0.0795	0.0169
Intraoperative blood loss	0.1021	0.0134
Intraoperative blood transfusions	-0.2637	0.0667
Surgery type (Ambulatory/Inpatient)	0.6959	0.0263
Intraoperative antiemetic prophylaxis	0.3182	0.0232
Postoperative nausea/vomiting in PACU	0.0852	0.0542
Intraoperative ketorolac dosage	-0.0099	0.0094
Intraoperative acetaminophen dosage	0.0269	0.0099
Intraoperative lidocaine dosage	-0.0176	0.0094
Intraoperative ketamine dosage	0.0345	0.0093
Intraoperative dexmedetomidine dosage	-0.0229	0.0100
Intraoperative esmolol dosage	-0.0033	0.0043
Intraoperative dexamethasone dosage	0.0416	0.0128
Difficult intubation	0.1349	0.1382
Conversion	0.1277	0.2900
Postoperative ketorolac dosage	0.1312	0.0095
Postoperative acetaminophen dosage	0.7964	0.0117
Postoperative gabapentin dosage	0.0998	0.0142
Midpoint of PACU stay, 12AM to 4AM	0.2057	0.0979
Midpoint of PACU stay, 4AM to 8AM	0.2973	0.1398
Midpoint of PACU stay, 8AM to 12PM	0.4157	0.0477
Midpoint of PACU stay, 12PM to 4PM	0.4137	0.0451
Midpoint of PACU stay, 4PM to 8PM	0.3525	0.0456
Midpoint of PACU stay, Sunday	-0.0874	0.0852
Midpoint of PACU stay, Monday	0.0715	0.0582
Midpoint of PACU stay, Tuesday	0.0725	0.0576
Midpoint of PACU stay, Wednesday	0.0602	0.0580
Midpoint of PACU stay, Thursday	-0.0588	0.0588
Midpoint of PACU stay, Friday	-0.0028	0.0575
Postoperative care location: PACU	0.0931	0.0364
Postoperative care location: CPC	-0.8377	0.0291

© 2023 Santa Cruz Mercado LA et al. *JAMA Surgery.*

E. PACU total MME (primary) log-normal

Coefficient	Value	Std. Error
(Intercept)	1.5045	0.0286
Intraoperative fentanyl exposure	-0.0391	0.0044
Intraoperative hydromorphone exposure	-0.0557	0.0064
Fentanyl effect site concentration at PACU admission	0.0898	0.0041
Hydromorphone effect site concentration at PACU admission	0.1268	0.0060
Intraoperative non-analgesic opioid usage	0.0566	0.0378
PACU non-analgesic opioid usage	-0.0099	0.0024
Intraoperative Vasopressors	-0.0048	0.0046
Intraoperative CYP3A4 Inducers	-0.0386	0.0097
Intraoperative CYP3A4 Inhibitors	-0.1831	0.0414
Intraoperative Antihypertensive Drugs	0.0653	0.0121
Naloxone usage	-0.2104	0.0626
Intraoperative hypotensive duration	-0.0057	0.0054
Intraoperative hypertensive duration	-0.0016	0.0046
Intraoperative tachycardic duration	0.0088	0.0047
Intraoperative bradycardic duration	-0.0122	0.0060
N2O dosage	0.0359	0.0040
Desflurane dosage	0.0150	0.0040
Isoflurane dosage	0.0087	0.0038
Propofol dosage	0.5488	0.0279
Sevoflurane dosage	0.0518	0.0045
Neuromuscular blocking agent usage	0.0137	0.0090
Intraoperative mean blood pressure	0.0198	0.0042
Intraoperative mean heart rate	-0.0181	0.0065
Surgical duration	-0.0282	0.0065
Intraoperative blood loss	0.0110	0.0041
Intraoperative blood transfusions	-0.0528	0.0271
Surgery type (Ambulatory/Inpatient)	0.2034	0.0099
Intraoperative antiemetic prophylaxis	0.0184	0.0093
Postoperative nausea/vomiting in PACU	-0.0295	0.0205
Intraoperative ketorolac dosage	0.0047	0.0039
Intraoperative acetaminophen dosage	0.0104	0.0041
Intraoperative lidocaine dosage	0.0004	0.0037
Intraoperative ketamine dosage	0.0366	0.0033
Intraoperative dexmedetomidine dosage	-0.0050	0.0043
Intraoperative esmolol dosage	0.0024	0.0017
Intraoperative dexamethasone dosage	-0.0398	0.0051
Difficult intubation	-0.1073	0.0503
Conversion	-0.2517	0.0873
Postoperative ketorolac dosage	0.0420	0.0030
Postoperative acetaminophen dosage	0.0952	0.0037
Postoperative gabapentin dosage	0.0440	0.0031
Midpoint of PACU stay, 12AM to 4AM	-0.2872	0.0364
Midpoint of PACU stay, 4AM to 8AM	-0.0801	0.0549
Midpoint of PACU stay, 8AM to 12PM	-0.0218	0.0192
Midpoint of PACU stay, 12PM to 4PM	0.0089	0.0179
Midpoint of PACU stay, 4PM to 8PM	-0.0604	0.0180
Midpoint of PACU stay, Sunday	0.0612	0.0348
Midpoint of PACU stay, Monday	-0.0153	0.0232
Midpoint of PACU stay, Tuesday	-0.0644	0.0229
Midpoint of PACU stay, Wednesday	-0.0035	0.0231
Midpoint of PACU stay, Thursday	-0.0907	0.0236
Midpoint of PACU stay, Friday	-0.0210	0.0230
Postoperative care location: PACU	0.1299	0.0130
Postoperative care location: CPC	-0.1783	0.0112

© 2023 Santa Cruz Mercado LA et al. *JAMA Surgery.*

F. 24h Postop total MME binomial

Coefficient	Value	Std. Error
(Intercept)	3.0788	0.0570
Intraoperative fentanyl exposure	-0.6529	0.0386
Intraoperative hydromorphone exposure	-1.1988	0.0307
PACU non-analgesic opioid usage	3.2384	0.6255
Intraoperative Vasopressors	0.0025	0.0218
Intraoperative CYP3A4 Inducers	-0.1328	0.0440
Intraoperative CYP3A4 Inhibitors	0.4692	0.2371
Intraoperative Antihypertensive Drugs	-0.0762	0.0547
Intraoperative hypotensive duration	0.0568	0.0218
Intraoperative hypertensive duration	0.0157	0.0187
Intraoperative tachycardic duration	0.0291	0.0252
Intraoperative bradycardic duration	-0.0338	0.0210
N2O dosage	0.0790	0.0177
Desflurane dosage	-0.0002	0.0177
Isoflurane dosage	0.0956	0.0193
Propofol dosage	-0.0925	0.1256
Sevoflurane dosage	0.2386	0.0225
Neuromuscular blocking agent usage	-0.0811	0.0402
Intraoperative mean blood pressure	0.0644	0.0200
Intraoperative mean heart rate	0.1193	0.0288
Surgical duration	-0.1731	0.0258
Intraoperative blood loss	0.1669	0.0244
Intraoperative blood transfusions	-0.0621	0.1011
Intraoperative antiemetic prophylaxis	0.4312	0.0420
Postoperative nausea/vomiting in PACU	-0.1238	0.0986
Intraoperative ketorolac dosage	0.0927	0.0204
Intraoperative acetaminophen dosage	-0.0308	0.0148
Intraoperative lidocaine dosage	0.0511	0.0265
Intraoperative ketamine dosage	0.1294	0.0165
Intraoperative dexmedetomidine dosage	0.0130	0.0147
Intraoperative esmolol dosage	0.5446	0.1486
Intraoperative dexamethasone dosage	-0.1361	0.0232
Difficult intubation	0.0578	0.2234
Conversion	-0.0701	0.4296
Postoperative ketorolac dosage	0.1363	0.0218
Postoperative acetaminophen dosage	0.2645	0.0202
Postoperative gabapentin dosage	0.1025	0.0285
Postoperative care location: PACU	-0.0420	0.0547
Postoperative care location: CPC	0.1736	0.0388
Naloxone usage	-0.9787	0.1299
Mean postop fentanyl effect site concentration	1.1864	0.0682
Mean postop hydromorphone effect site concentration	11.2038	0.2218

© 2023 Santa Cruz Mercado LA et al. *JAMA Surgery.*

G. 24h Postop total MME log-normal

Coefficient	Value	Std. Error
(Intercept)	2.3408	0.0119
Intraoperative fentanyl exposure	-0.1948	0.0092
Intraoperative hydromorphone exposure	0.0368	0.0055
PACU non-analgesic opioid usage	0.0073	0.0183
Intraoperative Vasopressors	-0.0132	0.0074
Intraoperative CYP3A4 Inducers	-0.0627	0.0149
Intraoperative CYP3A4 Inhibitors	-0.1822	0.0563
Intraoperative Antihypertensive Drugs	-0.0927	0.0163
Intraoperative hypotensive duration	-0.0157	0.0069
Intraoperative hypertensive duration	0.0096	0.0058
Intraoperative tachycardic duration	0.0221	0.0061
Intraoperative bradycardic duration	-0.0776	0.0074
N2O dosage	0.0719	0.0059
Desflurane dosage	0.0348	0.0058
Isoflurane dosage	0.0557	0.0062
Propofol dosage	0.5648	0.0403
Sevoflurane dosage	0.1450	0.0074
Neuromuscular blocking agent usage	-0.0972	0.0128
Intraoperative mean blood pressure	-0.0199	0.0062
Intraoperative mean heart rate	-0.0488	0.0093
Surgical duration	-0.0355	0.0078
Intraoperative blood loss	0.0177	0.0046
Intraoperative blood transfusions	-0.2100	0.0294
Intraoperative antiemetic prophylaxis	0.0298	0.0131
Postoperative nausea/vomiting in PACU	-0.1647	0.0311
Intraoperative ketorolac dosage	0.0221	0.0065
Intraoperative acetaminophen dosage	0.0088	0.0054
Intraoperative lidocaine dosage	0.0254	0.0054
Intraoperative ketamine dosage	0.0643	0.0040
Intraoperative dexmedetomidine dosage	0.0195	0.0047
Intraoperative esmolol dosage	0.0046	0.0023
Intraoperative dexamethasone dosage	-0.0356	0.0076
Difficult intubation	-0.1669	0.0724
Conversion	0.0259	0.1044
Postoperative ketorolac dosage	-0.0028	0.0049
Postoperative acetaminophen dosage	0.0708	0.0053
Postoperative gabapentin dosage	0.0678	0.0041
Postoperative care location: PACU	0.3242	0.0166
Postoperative care location: CPC	0.1172	0.0129
Naloxone usage	-0.5237	0.0499
Mean postop fentanyl effect site concentration	0.4259	0.0142
Mean postop hydromorphone effect site concentration	0.3968	0.0080

© 2023 Santa Cruz Mercado LA et al. *JAMA Surgery.*

H. In-hospital total MME binomial

Coefficient	Value	Std. Error
(Intercept)	1.3730	0.0365
Intraoperative fentanyl exposure	-0.3767	0.0316
Intraoperative hydromorphone exposure	-0.5052	0.0232
PACU non-analgesic opioid usage	0.0359	0.0554
Intraoperative Vasopressors	0.0049	0.0199
Intraoperative CYP3A4 Inducers	0.0666	0.0397
Intraoperative CYP3A4 Inhibitors	-0.3414	0.1712
Intraoperative Antihypertensive Drugs	-0.2239	0.0476
Intraoperative hypotensive duration	0.0169	0.0194
Intraoperative hypertensive duration	0.0224	0.0169
Intraoperative tachycardic duration	0.0095	0.0217
Intraoperative bradycardic duration	-0.0255	0.0192
N2O dosage	0.1041	0.0162
Desflurane dosage	-0.0115	0.0151
Isoflurane dosage	-0.0116	0.0168
Propofol dosage	0.0305	0.1125
Sevoflurane dosage	0.1383	0.0199
Neuromuscular blocking agent usage	-0.2099	0.0353
Intraoperative mean blood pressure	-0.0156	0.0176
Intraoperative mean heart rate	0.1955	0.0264
Surgical duration	-0.0616	0.0232
Intraoperative blood loss	0.1419	0.0221
Intraoperative blood transfusions	0.2720	0.1016
Intraoperative antiemetic prophylaxis	0.2341	0.0381
Postoperative nausea/vomiting in PACU	-0.0225	0.0859
Intraoperative ketorolac dosage	0.0454	0.0178
Intraoperative acetaminophen dosage	-0.0062	0.0136
Intraoperative lidocaine dosage	0.1142	0.0306
Intraoperative ketamine dosage	0.1027	0.0141
Intraoperative dexmedetomidine dosage	0.0428	0.0146
Intraoperative esmolol dosage	0.2907	0.1176
Intraoperative dexamethasone dosage	-0.1523	0.0207
Difficult intubation	-0.1541	0.1940
Conversion	0.4033	0.3610
Postoperative ketorolac dosage	0.0746	0.0162
Postoperative acetaminophen dosage	0.1213	0.0165
Postoperative gabapentin dosage	0.0856	0.0213
Postoperative care location: PACU	0.5287	0.0474
Postoperative care location: CPC	1.0481	0.0349
Naloxone usage	-0.0277	0.1347
Mean postop fentanyl effect site concentration	0.7124	0.0539
Mean postop hydromorphone effect site concentration	6.3149	0.1344

© 2023 Santa Cruz Mercado LA et al. *JAMA Surgery.*

I. In-hospital total MME log-normal

Coefficient	Value	Std. Error
(Intercept)	2.4998	0.0185
Intraoperative fentanyl exposure	-0.1967	0.0129
Intraoperative hydromorphone exposure	0.0963	0.0076
PACU non-analgesic opioid usage	-0.0837	0.0299
Intraoperative Vasopressors	0.0227	0.0123
Intraoperative CYP3A4 Inducers	-0.1901	0.0225
Intraoperative CYP3A4 Inhibitors	0.2835	0.0882
Intraoperative Antihypertensive Drugs	-0.1320	0.0247
Intraoperative hypotensive duration	-0.0170	0.0102
Intraoperative hypertensive duration	0.0464	0.0087
Intraoperative tachycardic duration	0.0091	0.0090
Intraoperative bradycardic duration	-0.0764	0.0112
N2O dosage	0.1568	0.0087
Desflurane dosage	0.0208	0.0089
Isoflurane dosage	0.0356	0.0094
Propofol dosage	0.3243	0.0602
Sevoflurane dosage	0.1515	0.0110
Neuromuscular blocking agent usage	-0.2400	0.0194
Intraoperative mean blood pressure	-0.0439	0.0093
Intraoperative mean heart rate	0.0209	0.0138
Surgical duration	-0.0622	0.0115
Intraoperative blood loss	0.1083	0.0068
Intraoperative blood transfusions	0.2737	0.0417
Intraoperative antiemetic prophylaxis	0.1519	0.0193
Postoperative nausea/vomiting in PACU	-0.2784	0.0473
Intraoperative ketorolac dosage	-0.0583	0.0097
Intraoperative acetaminophen dosage	-0.0150	0.0081
Intraoperative lidocaine dosage	0.0335	0.0079
Intraoperative ketamine dosage	0.1388	0.0060
Intraoperative dexmedetomidine dosage	0.0192	0.0070
Intraoperative esmolol dosage	0.0037	0.0034
Intraoperative dexamethasone dosage	-0.0445	0.0114
Difficult intubation	-0.0434	0.1149
Conversion	0.0701	0.1561
Postoperative ketorolac dosage	-0.0332	0.0074
Postoperative acetaminophen dosage	0.0682	0.0080
Postoperative gabapentin dosage	0.1015	0.0062
Postoperative care location: PACU	0.7995	0.0250
Postoperative care location: CPC	0.6872	0.0197
Naloxone usage	0.9412	0.0689
Mean postop fentanyl effect site concentration	0.3953	0.0191
Mean postop hydromorphone effect site concentration	0.3677	0.0083

© 2023 Santa Cruz Mercado LA et al. *JAMA Surgery.*

J. Time to Recovery from sedation

Coefficient	Value	Std. Error
(Intercept)	3.8725	0.0077
Intraoperative fentanyl exposure	0.0230	0.0051
Intraoperative hydromorphone exposure	-0.0134	0.0077
Fentanyl effect site concentration at PACU admission	-0.0263	0.0053
Hydromorphone effect site concentration at PACU admission	0.0404	0.0075
Intraoperative non-analgesic opioid usage	-0.3035	0.0562
PACU non-analgesic opioid usage	-0.0098	0.0173
Intraoperative Vasopressors	0.0217	0.0111
Intraoperative CYP3A4 Inducers	0.0012	0.0106
Intraoperative CYP3A4 Inhibitors	0.0620	0.0505
Intraoperative Antihypertensive Drugs	0.0520	0.0143
Naloxone usage	0.4744	0.0508
Intraoperative hypotensive duration	0.0339	0.0064
Intraoperative hypertensive duration	0.0415	0.0054
Intraoperative tachycardic duration	-0.0031	0.0061
Intraoperative bradycardic duration	0.0163	0.0066
N2O dosage	-0.0396	0.0044
Desflurane dosage	0.0084	0.0050
Isoflurane dosage	0.0293	0.0049
Propofol dosage	-0.5102	0.0289
Sevoflurane dosage	-0.0110	0.0051
Neuromuscular blocking agent usage	0.0999	0.0104
Intraoperative mean blood pressure	-0.0038	0.0048
Intraoperative mean heart rate	0.0759	0.0072
Surgical duration	0.1304	0.0078
Intraoperative blood loss	0.0068	0.0057
Intraoperative blood transfusions	-0.0934	0.0290
Surgery type (Ambulatory/Inpatient)	0.1185	0.0113
Intraoperative antiemetic prophylaxis	0.0165	0.0102
Intraoperative ketorolac dosage	0.0100	0.0042
Intraoperative acetaminophen dosage	0.0003	0.0045
Intraoperative lidocaine dosage	0.0049	0.0042
Intraoperative ketamine dosage	0.0159	0.0039
Intraoperative dexmedetomidine dosage	0.0153	0.0049
Intraoperative esmolol dosage	-0.0043	0.0023
Intraoperative dexamethasone dosage	0.0654	0.0058
Difficult intubation	0.2366	0.0617
Conversion	0.2963	0.1123
Postoperative ketorolac dosage	0.0269	0.0038
Postoperative acetaminophen dosage	0.0437	0.0044
Postoperative gabapentin dosage	0.0117	0.0040
Postoperative care location: PACU	-0.1562	0.0150
Postoperative care location: CPC	-0.0073	0.0125

© 2023 Santa Cruz Mercado LA et al. *JAMA Surgery.*

K. PACU length of stay

Coefficient	Value	Std. Error
(Intercept)	4.4539	0.0080
Intraoperative fentanyl exposure	0.0032	0.0038
Intraoperative hydromorphone exposure	0.0180	0.0038
Intraoperative non-analgesic opioid usage	-0.1448	0.0542
PACU non-analgesic opioid usage	0.0190	0.0169
Intraoperative Vasopressors	0.0362	0.0110
Intraoperative CYP3A4 Inducers	-0.0141	0.0106
Intraoperative CYP3A4 Inhibitors	-0.0326	0.0504
Intraoperative Antihypertensive Drugs	0.0992	0.0142
Intraoperative hypotensive duration	0.0245	0.0063
Intraoperative hypertensive duration	0.0277	0.0054
Intraoperative tachycardic duration	0.0023	0.0055
Intraoperative bradycardic duration	0.0352	0.0066
N2O dosage	-0.0104	0.0043
Desflurane dosage	0.0044	0.0104
Isoflurane dosage	0.0114	0.0047
Propofol dosage	-0.4337	0.0295
Sevoflurane dosage	-0.0217	0.0050
Neuromuscular blocking agent usage	0.0697	0.0104
Intraoperative mean blood pressure	0.0198	0.0047
Intraoperative mean heart rate	0.0383	0.0070
Surgical duration	0.0690	0.0075
Intraoperative blood loss	0.0200	0.0056
Intraoperative blood transfusions	-0.0430	0.0291
Surgery type (Ambulatory/Inpatient)	0.1980	0.0111
Intraoperative antiemetic prophylaxis	-0.0133	0.0102
Intraoperative ketorolac dosage	-0.0080	0.0042
Intraoperative acetaminophen dosage	0.0091	0.0045
Intraoperative lidocaine dosage	0.0070	0.0041
Intraoperative ketamine dosage	-0.0017	0.0039
Intraoperative dexmedetomidine dosage	0.0103	0.0046
Intraoperative esmolol dosage	-0.0004	0.0022
Intraoperative dexamethasone dosage	0.0330	0.0057
Difficult intubation	0.1753	0.0608
Conversion	-0.0853	0.1122
Postoperative ketorolac dosage	0.0065	0.0008
Postoperative acetaminophen dosage	0.0004	0.0000
Postoperative gabapentin dosage	0.0003	0.0001
Postoperative care location: PACU	-0.1286	0.0148
Postoperative care location: CPC	-0.1440	0.0124

© 2023 Santa Cruz Mercado LA et al. *JAMA Surgery.*

L. Hospital length of stay

Coefficient	Value	Std. Error
(Intercept)	7.9377	0.0131
Intraoperative fentanyl exposure	-0.0367	0.0054
Intraoperative hydromorphone exposure	0.0769	0.0047
Intraoperative non-analgesic opioid usage	0.0703	0.0533
PACU non-analgesic opioid usage	-0.0420	0.0207
Intraoperative Vasopressors	0.1776	0.0198
Intraoperative CYP3A4 Inducers	-0.0437	0.0153
Intraoperative CYP3A4 Inhibitors	0.2756	0.0615
Intraoperative Antihypertensive Drugs	-0.0413	0.0170
Intraoperative hypotensive duration	-0.0268	0.0069
Intraoperative hypertensive duration	0.0253	0.0062
Intraoperative tachycardic duration	-0.0307	0.0061
Intraoperative bradycardic duration	0.0156	0.0073
N2O dosage	0.0458	0.0060
Desflurane dosage	-0.0489	0.0123
Isoflurane dosage	-0.0680	0.0065
Propofol dosage	-0.4941	0.0419
Sevoflurane dosage	-0.0434	0.0078
Neuromuscular blocking agent usage	-0.1606	0.0135
Intraoperative mean blood pressure	-0.0123	0.0064
Intraoperative mean heart rate	0.1076	0.0094
Surgical duration	-0.0137	0.0079
Intraoperative blood loss	0.0836	0.0058
Intraoperative blood transfusions	0.7257	0.0299
Intraoperative antiemetic prophylaxis	0.4460	0.0139
Intraoperative ketorolac dosage	-0.1202	0.0068
Intraoperative acetaminophen dosage	-0.0028	0.0054
Intraoperative lidocaine dosage	0.0234	0.0076
Intraoperative ketamine dosage	0.0390	0.0044
Intraoperative dexmedetomidine dosage	-0.0015	0.0048
Intraoperative esmolol dosage	-0.0016	0.0027
Intraoperative dexamethasone dosage	-0.1073	0.0078
Difficult intubation	-0.0215	0.0754
Conversion	0.2713	0.1145
Postoperative ketorolac dosage	-0.0049	0.0012
Postoperative acetaminophen dosage	0.0001	0.0000
Postoperative gabapentin dosage	0.0003	0.0001
Postoperative care location: PACU	0.9718	0.0174
Postoperative care location: CPC	0.8484	0.0138

© 2023 Santa Cruz Mercado LA et al. *JAMA Surgery.*

M. Frequency of uncontrolled pain

Coefficient	Value	Std. Error
(Intercept)	-7.5144	11.9494
Intraoperative fentanyl exposure	-0.6668	0.1033
Intraoperative hydromorphone exposure	0.0834	0.0652
Pain scale (verbal/numeric)	0.6324	0.3243
Intraoperative non-analgesic opioid usage	-12.4919	913.8451
PACU non-analgesic opioid usage	-0.0931	0.3467
Intraoperative Vasopressors	-1.2317	0.5129
Intraoperative CYP3A4 Inducers	0.4646	0.2574
Intraoperative CYP3A4 Inhibitors	-13.7197	570.6342
Intraoperative Antihypertensive Drugs	1.2130	0.2049
Intraoperative hypotensive duration	-0.0722	0.0883
Intraoperative hypertensive duration	0.1913	0.0509
Intraoperative tachycardic duration	-0.4150	0.1738
Intraoperative bradycardic duration	-0.1019	0.1116
N2O dosage	-0.0630	0.0831
Isoflurane dosage	-0.0936	0.0993
Propofol dosage	0.5614	0.5902
Sevoflurane dosage	0.0225	0.1115
Neuromuscular blocking agent usage	-0.2190	0.1921
Intraoperative mean blood pressure	-0.2144	0.0911
Intraoperative mean heart rate	0.0961	0.1539
Surgical duration	0.0572	0.1050
Intraoperative blood loss	0.0156	0.0617
Intraoperative blood transfusions	0.6860	0.3284
Intraoperative antiemetic prophylaxis	-1.0943	0.2371
Intraoperative ketorolac dosage	-0.1465	0.1155
Intraoperative acetaminophen dosage	0.0747	0.0761
Intraoperative lidocaine dosage	0.0648	0.0170
Intraoperative ketamine dosage	0.0783	0.0468
Intraoperative dexmedetomidine dosage	0.0472	0.0611
Intraoperative esmolol dosage	-1.9165	1.1031
Intraoperative dexamethasone dosage	-0.9433	0.1315
Difficult intubation	1.2200	0.7159
Conversion	0.7517	1.0344
Postoperative ketorolac dosage	0.0075	0.0139
Postoperative acetaminophen dosage	0.0009	0.0002
Postoperative gabapentin dosage	0.0020	0.0003
Time of day of max pain, 12AM to 4AM	-0.5925	0.8312
Time of day of max pain, 4AM to 8AM	-14.5075	650.1206
Time of day of max pain, 8AM to 12PM	0.1192	0.3221
Time of day of max pain, 12PM to 4PM	-0.3866	0.2937
Time of day of max pain, 4PM to 8PM	-0.6026	0.3008
Day of week of max pain, Sunday	-0.5001	0.8823
Day of week of max pain, Monday	0.0501	0.5269
Day of week of max pain, Tuesday	0.9453	0.4854
Day of week of max pain, Wednesday	0.5830	0.4951
Day of week of max pain, Thursday	0.8984	0.5035
Day of week of max pain, Friday	0.0687	0.5102
Postoperative care location: PACU	0.6704	0.2307
Postoperative care location: CPC	0.2054	0.2020
Naloxone usage	0.0865	0.0470
Total opioid administration between time of extubation and PACU admission	-0.1791	0.1790
Total opioid administration between PACU admission and 24 hours postop	0.0568	0.0485
Mean postop fentanyl effect site concentration	0.6579	0.0996
Mean postop hydromorphone effect site concentration	0.2276	0.0501

© 2023 Santa Cruz Mercado LA et al. *JAMA Surgery.*

N. 3-month chronic pain

Coefficient	Value	Std. Error
(Intercept)	-7.1850	31.3663
Intraoperative fentanyl exposure	-0.1723	0.0600
Intraoperative hydromorphone exposure	0.1419	0.0463
Pain scale (verbal/numeric)	0.5151	0.1830
PACU non-analgesic opioid usage	-22.8867	718.6636
Intraoperative Vasopressors	0.0446	0.0429
Intraoperative CYP3A4 Inducers	-0.3344	0.1878
Intraoperative CYP3A4 Inhibitors	-0.3039	1.0095
Intraoperative Antihypertensive Drugs	0.8060	0.1917
Intraoperative hypotensive duration	-0.1199	0.1188
Intraoperative hypertensive duration	-0.0456	0.0890
Intraoperative tachycardic duration	0.0185	0.1180
Intraoperative bradycardic duration	-0.4392	0.1357
N2O dosage	0.1311	0.0602
Isoflurane dosage	0.0469	0.0254
Propofol dosage	-0.7422	0.5055
Sevoflurane dosage	0.0313	0.0706
Neuromuscular blocking agent usage	0.2643	0.1669
Intraoperative mean blood pressure	-0.0422	0.0714
Intraoperative mean heart rate	-0.4816	0.1184
Surgical duration	-0.3262	0.1391
Intraoperative blood loss	-0.0866	0.1200
Intraoperative blood transfusions	-0.8077	0.6028
Surgery type (Ambulatory/Inpatient)	-0.2835	0.1875
Intraoperative antiemetic prophylaxis	0.2167	0.1472
Intraoperative ketorolac dosage	-0.0684	0.0685
Intraoperative acetaminophen dosage	-4.6679	160.7067
Intraoperative lidocaine dosage	0.0036	0.0639
Intraoperative ketamine dosage	0.1300	0.0395
Intraoperative esmolol dosage	-0.0102	0.0687
Intraoperative dexamethasone dosage	-0.0550	0.0875
Postoperative ketorolac dosage	-0.0237	0.0163
Postoperative acetaminophen dosage	0.0002	0.0002
Postoperative gabapentin dosage	0.0019	0.0003
Postoperative care location: PACU	0.6775	0.2091
Postoperative care location: CPC	0.3658	0.2081
Total opioid administration between time of extubation and PACU admission	0.0581	0.1115
Total opioid administration between PACU admission and hospital discharge	0.1392	0.0325

© 2023 Santa Cruz Mercado LA et al. *JAMA Surgery*.

O. 12-month chronic pain

Coefficient	Value	Std. Error
(Intercept)	-4.3069	2.8795
Intraoperative fentanyl exposure	-0.0601	0.0291
Intraoperative hydromorphone exposure	-0.0833	0.0300
Pain scale (verbal/numeric)	0.2654	0.0803
Intraoperative non-analgesic opioid usage	-8.7713	220.4613
PACU non-analgesic opioid usage	-1.1751	0.3559
Intraoperative Vasopressors	0.0464	0.0204
Intraoperative CYP3A4 Inducers	-0.3092	0.0868
Intraoperative CYP3A4 Inhibitors	-0.5595	0.5086
Intraoperative Antihypertensive Drugs	0.3564	0.1066
Intraoperative hypotensive duration	-0.3003	0.0519
Intraoperative hypertensive duration	-0.1254	0.0404
Intraoperative tachycardic duration	-0.2525	0.0741
Intraoperative bradycardic duration	0.1392	0.0466
N2O dosage	0.0172	0.0309
Isoflurane dosage	-0.0017	0.0336
Propofol dosage	-0.1209	0.2322
Sevoflurane dosage	-0.0333	0.0359
Neuromuscular blocking agent usage	-0.0198	0.0791
Intraoperative mean blood pressure	0.0977	0.0337
Intraoperative mean heart rate	0.0533	0.0544
Surgical duration	0.0697	0.0510
Intraoperative blood loss	-0.0624	0.0492
Intraoperative blood transfusions	-0.0308	0.2290
Surgery type (Ambulatory/Inpatient)	-0.6531	0.0915
Intraoperative antiemetic prophylaxis	0.3225	0.0721
Intraoperative ketorolac dosage	-0.0915	0.0327
Intraoperative acetaminophen dosage	-0.2616	0.0673
Intraoperative lidocaine dosage	0.0344	0.0161
Intraoperative ketamine dosage	0.1169	0.0218
Intraoperative dexmedetomidine dosage	-0.2359	0.0984
Intraoperative esmolol dosage	-0.7524	0.4760
Intraoperative dexamethasone dosage	-0.0311	0.0420
Difficult intubation	0.0368	0.4793
Conversion	-12.4314	259.1249
Postoperative ketorolac dosage	0.0156	0.0052
Postoperative acetaminophen dosage	0.0001	0.0001
Postoperative gabapentin dosage	0.0017	0.0002
Postoperative care location: PACU	0.2176	0.1156
Postoperative care location: CPC	0.4774	0.1006
Naloxone usage	-0.0986	0.0471
Total opioid administration between time of extubation and PACU admission	-0.0523	0.0706
Total opioid administration between PACU admission and hospital discharge	0.0974	0.0284

© 2023 Santa Cruz Mercado LA et al. *JAMA Surgery*.

P. 30-day Opioid Prescription

Coefficient	Value	Std. Error
(Intercept)	-1.2843	0.0186
Intraoperative fentanyl exposure	-0.0813	0.0091
Intraoperative hydromorphone exposure	0.0455	0.0085
Intraoperative non-analgesic opioid usage	-0.1091	0.1726
PACU non-analgesic opioid usage	-0.0619	0.0415
Intraoperative Vasopressors	0.0365	0.0140
Intraoperative CYP3A4 Inducers	0.1637	0.0248
Intraoperative CYP3A4 Inhibitors	-0.4548	0.1298
Intraoperative Antihypertensive Drugs	-0.0931	0.0330
Intraoperative hypotensive duration	0.0285	0.0143
Intraoperative hypertensive duration	0.0803	0.0116
Intraoperative tachycardic duration	0.0634	0.0118
Intraoperative bradycardic duration	-0.0419	0.0147
N2O dosage	0.0261	0.0099
Desflurane dosage	-0.0397	0.0129
Isoflurane dosage	-0.0304	0.0112
Propofol dosage	-0.2778	0.0718
Sevoflurane dosage	-0.0418	0.0120
Neuromuscular blocking agent usage	-0.2080	0.0245
Intraoperative mean blood pressure	0.0109	0.0109
Intraoperative mean heart rate	-0.0338	0.0159
Surgical duration	-0.0212	0.0166
Intraoperative blood loss	0.0213	0.0106
Intraoperative blood transfusions	0.1037	0.0619
Surgery type (Ambulatory/Inpatient)	0.2616	0.0270
Intraoperative antiemetic prophylaxis	0.0466	0.0237
Postoperative nausea/vomiting in PACU	-0.1323	0.0579
Intraoperative ketorolac dosage	-0.0637	0.0103
Intraoperative acetaminophen dosage	-0.0136	0.0107
Intraoperative lidocaine dosage	0.0043	0.0090
Intraoperative ketamine dosage	0.0709	0.0084
Intraoperative dexmedetomidine dosage	-0.0032	0.0104
Intraoperative esmolol dosage	0.0056	0.0041
Intraoperative dexamethasone dosage	-0.1752	0.0135
Difficult intubation	0.0444	0.1399
Conversion	0.4874	0.2355
Postoperative ketorolac dosage	0.0104	0.0089
Postoperative acetaminophen dosage	0.0670	0.0101
Postoperative gabapentin dosage	0.0927	0.0099
Postoperative care location: PACU	0.1613	0.0343
Postoperative care location: CPC	0.3128	0.0283
Naloxone usage	-0.3866	0.0904
Total opioid administration between time of extubation and PACU admission	-0.0713	0.0223
Total opioid administration between PACU admission and hospital discharge	0.1702	0.0229

© 2023 Santa Cruz Mercado LA et al. *JAMA Surgery.*

Q. 90-day Opioid Prescription

Coefficient	Value	Std. Error
(Intercept)	-1.2037	0.0184
Intraoperative fentanyl exposure	-0.0917	0.0090
Intraoperative hydromorphone exposure	0.0624	0.0084
Intraoperative non-analgesic opioid usage	-0.1100	0.1751
PACU non-analgesic opioid usage	-0.0794	0.0413
Intraoperative Vasopressors	0.0364	0.0141
Intraoperative CYP3A4 Inducers	0.1599	0.0244
Intraoperative CYP3A4 Inhibitors	-0.5098	0.1287
Intraoperative Antihypertensive Drugs	-0.0640	0.0324
Intraoperative hypotensive duration	0.0218	0.0141
Intraoperative hypertensive duration	0.0667	0.0115
Intraoperative tachycardic duration	0.0546	0.0117
Intraoperative bradycardic duration	-0.0425	0.0145
N2O dosage	0.0292	0.0097
Desflurane dosage	-0.0362	0.0125
Isoflurane dosage	-0.0300	0.0110
Propofol dosage	-0.3287	0.0708
Sevoflurane dosage	-0.0528	0.0118
Neuromuscular blocking agent usage	-0.2022	0.0241
Intraoperative mean blood pressure	0.0162	0.0107
Intraoperative mean heart rate	-0.0324	0.0156
Surgical duration	-0.0359	0.0164
Intraoperative blood loss	0.0552	0.0112
Intraoperative blood transfusions	0.3230	0.0605
Surgery type (Ambulatory/Inpatient)	0.2525	0.0266
Intraoperative antiemetic prophylaxis	0.0291	0.0234
Postoperative nausea/vomiting in PACU	-0.1808	0.0577
Intraoperative ketorolac dosage	-0.0706	0.0101
Intraoperative acetaminophen dosage	-0.0199	0.0106
Intraoperative lidocaine dosage	0.0010	0.0092
Intraoperative ketamine dosage	0.0773	0.0083
Intraoperative dexmedetomidine dosage	0.0005	0.0101
Intraoperative esmolol dosage	0.0052	0.0041
Intraoperative dexamethasone dosage	-0.1758	0.0132
Difficult intubation	0.0730	0.1369
Conversion	0.4021	0.2362
Postoperative ketorolac dosage	0.0015	0.0088
Postoperative acetaminophen dosage	0.0572	0.0100
Postoperative gabapentin dosage	0.0986	0.0101
Postoperative care location: PACU	0.1467	0.0339
Postoperative care location: CPC	0.3298	0.0279
Naloxone usage	-0.2042	0.0855
Total opioid administration between time of extubation and PACU admission	-0.0892	0.0230
Total opioid administration between PACU admission and hospital discharge	0.2685	0.0334

© 2023 Santa Cruz Mercado LA et al. *JAMA Surgery*.

R. 180-day Opioid Prescription

Coefficient	Value	Std. Error
(Intercept)	-1.1194	0.0180
Intraoperative fentanyl exposure	-0.0860	0.0088
Intraoperative hydromorphone exposure	0.0545	0.0083
Intraoperative non-analgesic opioid usage	0.2296	0.1305
PACU non-analgesic opioid usage	-0.0947	0.0409
Intraoperative Vasopressors	0.0352	0.0141
Intraoperative CYP3A4 Inducers	0.1863	0.0240
Intraoperative CYP3A4 Inhibitors	-0.4952	0.1258
Intraoperative Antihypertensive Drugs	-0.0873	0.0321
Intraoperative hypotensive duration	0.0259	0.0140
Intraoperative hypertensive duration	0.0679	0.0114
Intraoperative tachycardic duration	0.0613	0.0117
Intraoperative bradycardic duration	-0.0475	0.0144
N2O dosage	0.0313	0.0096
Desflurane dosage	-0.0302	0.0122
Isoflurane dosage	-0.0254	0.0107
Propofol dosage	-0.4430	0.0701
Sevoflurane dosage	-0.0500	0.0116
Neuromuscular blocking agent usage	-0.1958	0.0238
Intraoperative mean blood pressure	0.0217	0.0106
Intraoperative mean heart rate	-0.0363	0.0154
Surgical duration	-0.0443	0.0163
Intraoperative blood loss	0.0729	0.0116
Intraoperative blood transfusions	0.3251	0.0603
Surgery type (Ambulatory/Inpatient)	0.2321	0.0262
Intraoperative antiemetic prophylaxis	0.0615	0.0231
Postoperative nausea/vomiting in PACU	-0.2235	0.0573
Intraoperative ketorolac dosage	-0.0832	0.0100
Intraoperative acetaminophen dosage	-0.0173	0.0104
Intraoperative lidocaine dosage	-0.0044	0.0095
Intraoperative ketamine dosage	0.0834	0.0083
Intraoperative dexmedetomidine dosage	-0.0031	0.0102
Intraoperative esmolol dosage	0.0051	0.0041
Intraoperative dexamethasone dosage	-0.1742	0.0131
Difficult intubation	0.0885	0.1346
Conversion	0.4688	0.2337
Postoperative ketorolac dosage	0.0001	0.0087
Postoperative acetaminophen dosage	0.0459	0.0099
Postoperative gabapentin dosage	0.0973	0.0101
Postoperative care location: PACU	0.1105	0.0336
Postoperative care location: CPC	0.2941	0.0276
Naloxone usage	-0.2057	0.0849
Total opioid administration between time of extubation and PACU admission	-0.0739	0.0219
Total opioid administration between PACU admission and hospital discharge	0.2924	0.0353

© 2023 Santa Cruz Mercado LA et al. *JAMA Surgery.*

S. Persistent use

Coefficient	Value	Std. Error
(Intercept)	-1.6853	0.0210
Intraoperative fentanyl exposure	-0.0733	0.0104
Intraoperative hydromorphone exposure	0.0726	0.0094
Intraoperative non-analgesic opioid usage	0.3541	0.1173
PACU non-analgesic opioid usage	-0.1180	0.0498
Intraoperative Vasopressors	-0.0043	0.0171
Intraoperative CYP3A4 Inducers	0.1220	0.0284
Intraoperative CYP3A4 Inhibitors	-0.5436	0.1611
Intraoperative Antihypertensive Drugs	0.0379	0.0374
Intraoperative hypotensive duration	-0.0042	0.0169
Intraoperative hypertensive duration	0.0807	0.0129
Intraoperative tachycardic duration	-0.0285	0.0159
Intraoperative bradycardic duration	-0.0335	0.0170
N2O dosage	0.0357	0.0112
Desflurane dosage	-0.0114	0.0145
Isoflurane dosage	-0.0048	0.0122
Propofol dosage	-0.4293	0.0828
Sevoflurane dosage	-0.0021	0.0136
Neuromuscular blocking agent usage	0.0004	0.0280
Intraoperative mean blood pressure	0.0313	0.0124
Intraoperative mean heart rate	-0.0804	0.0187
Surgical duration	-0.0802	0.0197
Intraoperative blood loss	0.0496	0.0117
Intraoperative blood transfusions	0.3708	0.0674
Surgery type (Ambulatory/Inpatient)	0.1321	0.0306
Intraoperative antiemetic prophylaxis	0.0426	0.0274
Postoperative nausea/vomiting in PACU	-0.2186	0.0690
Intraoperative ketorolac dosage	-0.0878	0.0119
Intraoperative acetaminophen dosage	-0.0170	0.0125
Intraoperative lidocaine dosage	0.0173	0.0091
Intraoperative ketamine dosage	0.0508	0.0095
Intraoperative dexmedetomidine dosage	-0.0066	0.0126
Intraoperative esmolol dosage	0.0098	0.0041
Intraoperative dexamethasone dosage	-0.1628	0.0155
Difficult intubation	0.3048	0.1458
Conversion	0.4510	0.2582
Postoperative ketorolac dosage	0.0079	0.0101
Postoperative acetaminophen dosage	0.0099	0.0117
Postoperative gabapentin dosage	0.0583	0.0096
Postoperative care location: PACU	-0.1999	0.0412
Postoperative care location: CPC	0.0211	0.0327
Naloxone usage	-0.0216	0.0988

© 2023 Santa Cruz Mercado LA et al. *JAMA Surgery.*

T. PONV in PACU

Coefficient	Value	Std. Error
(Intercept)	-3.9588	1.7807
Intraoperative fentanyl exposure	-0.0237	0.0285
Intraoperative hydromorphone exposure	0.2047	0.0379
Fentanyl effect site concentration at PACU admission	0.1038	0.0256
Hydromorphone effect site concentration at PACU admission	-0.1319	0.0407
Intraoperative non-analgesic opioid usage	-8.4226	136.3432
PACU non-analgesic opioid usage	-0.0915	0.1013
Intraoperative Vasopressors	-0.7550	0.1667
Intraoperative CYP3A4 Inducers	0.0973	0.0605
Intraoperative CYP3A4 Inhibitors	0.9717	0.1908
Intraoperative Antihypertensive Drugs	0.2012	0.0768
Naloxone usage	-0.1029	0.2840
Intraoperative hypotensive duration	0.2088	0.0296
Intraoperative hypertensive duration	0.0788	0.0252
Intraoperative tachycardic duration	-0.0720	0.0292
Intraoperative bradycardic duration	0.2102	0.0317
N2O dosage	0.1890	0.0234
Desflurane dosage	0.1217	0.0187
Isoflurane dosage	0.0299	0.0186
Propofol dosage	-1.3917	0.1879
Sevoflurane dosage	0.0578	0.0276
Neuromuscular blocking agent usage	0.2328	0.0570
Intraoperative mean blood pressure	0.0691	0.0278
Intraoperative mean heart rate	0.2986	0.0392
Surgical duration	0.0668	0.0363
Intraoperative blood loss	-0.2036	0.0505
Intraoperative blood transfusions	-1.3473	0.3003
Surgery type (Ambulatory/Inpatient)	0.0680	0.0642
Intraoperative antiemetic prophylaxis	0.3770	0.0572
Intraoperative ketorolac dosage	0.2358	0.0232
Intraoperative acetaminophen dosage	0.0434	0.0231
Intraoperative lidocaine dosage	0.0096	0.0188
Intraoperative ketamine dosage	-0.1927	0.0348
Intraoperative dexmedetomidine dosage	-0.0513	0.0335
Intraoperative esmolol dosage	-0.3200	0.2117
Intraoperative dexamethasone dosage	-0.0683	0.0343
Difficult intubation	0.2079	0.3296
Conversion	0.5826	0.4720
Postoperative ketorolac dosage	0.1375	0.0167
Postoperative acetaminophen dosage	0.0713	0.0246
Postoperative gabapentin dosage	0.0473	0.0158
Postoperative care location: PACU	-0.2391	0.0891
Postoperative care location: CPC	-0.2939	0.0714

© 2023 Santa Cruz Mercado LA et al. *JAMA Surgery.*

U. Respiratory depression

Coefficient	Value	Std. Error
(Intercept)	-4.6307	1.7073
Intraoperative fentanyl exposure	0.0043	0.0423
Intraoperative hydromorphone exposure	0.2548	0.0631
Fentanyl effect site concentration at PACU admission	0.0759	0.0412
Hydromorphone effect site concentration at PACU admission	-0.2900	0.0655
Intraoperative non-analgesic opioid usage	-8.0940	130.6636
PACU non-analgesic opioid usage	-0.3461	0.1933
Intraoperative Vasopressors	0.0396	0.0287
Intraoperative CYP3A4 Inducers	0.3022	0.0838
Intraoperative CYP3A4 Inhibitors	0.4992	0.2956
Intraoperative Antihypertensive Drugs	0.1722	0.1161
Naloxone usage	2.1963	0.1662
Intraoperative hypotensive duration	-0.1252	0.0460
Intraoperative hypertensive duration	0.0428	0.0337
Intraoperative tachycardic duration	-0.0884	0.0418
Intraoperative bradycardic duration	-0.0805	0.0515
N2O dosage	-0.1252	0.0384
Desflurane dosage	-0.0469	0.0473
Isoflurane dosage	0.1838	0.0280
Propofol dosage	0.2161	0.2447
Sevoflurane dosage	-0.0507	0.0397
Neuromuscular blocking agent usage	-0.0274	0.0850
Intraoperative mean blood pressure	0.0178	0.0385
Intraoperative mean heart rate	0.1060	0.0577
Surgical duration	0.1649	0.0459
Intraoperative blood loss	0.1020	0.0204
Intraoperative blood transfusions	0.6619	0.1744
Surgery type (Ambulatory/Inpatient)	0.0258	0.0997
Intraoperative antiemetic prophylaxis	-0.3956	0.0935
Intraoperative ketorolac dosage	0.0082	0.0360
Intraoperative acetaminophen dosage	-0.1186	0.0475
Intraoperative lidocaine dosage	-0.2634	0.0915
Intraoperative ketamine dosage	0.1192	0.0209
Intraoperative dexmedetomidine dosage	0.0296	0.0287
Intraoperative esmolol dosage	-1.3444	0.5667
Intraoperative dexamethasone dosage	-0.0215	0.0487
Difficult intubation	0.9513	0.2659
Conversion	0.6694	0.6039
Postoperative ketorolac dosage	0.0143	0.0303
Postoperative acetaminophen dosage	-0.0190	0.0361
Postoperative gabapentin dosage	-0.0009	0.0298
Postoperative care location: PACU	-0.2248	0.1417
Postoperative care location: CPC	0.2751	0.1000

© 2023 Santa Cruz Mercado LA et al. *JAMA Surgery.*

V. 30-day postoperative mortality

Coefficient	Value	Std. Error
(Intercept)	-11.4937	76.6772
Intraoperative fentanyl exposure	0.1449	0.0729
Intraoperative hydromorphone exposure	-0.3366	0.0931
Intraoperative non-analgesic opioid usage	-9.6331	5,872.7358
PACU non-analgesic opioid usage	-16.1193	1,416.8145
Intraoperative Vasopressors	0.0334	0.0570
Intraoperative CYP3A4 Inducers	-0.3920	0.3461
Intraoperative CYP3A4 Inhibitors	-17.7333	3,046.0472
Intraoperative Antihypertensive Drugs	-0.5482	0.3915
Intraoperative hypotensive duration	-0.3659	0.1506
Intraoperative hypertensive duration	-0.0895	0.1052
Intraoperative tachycardic duration	0.2397	0.0490
Intraoperative bradycardic duration	0.4146	0.1089
N2O dosage	-0.1341	0.0798
Isoflurane dosage	-0.5458	0.1541
Propofol dosage	-7.2028	0.7570
Sevoflurane dosage	-0.4061	0.1085
Neuromuscular blocking agent usage	-0.3109	0.2528
Intraoperative mean blood pressure	-0.3386	0.0980
Intraoperative mean heart rate	0.3868	0.0915
Surgical duration	-0.0121	0.1537
Intraoperative blood loss	0.0782	0.0252
Intraoperative blood transfusions	1.0455	0.2227
Surgery type (Ambulatory/Inpatient)	3.4856	1.0386
Intraoperative antiemetic prophylaxis	0.2844	0.1782
Postoperative nausea/vomiting in PACU	-0.1752	0.7262
Intraoperative ketorolac dosage	-0.8034	0.2524
Intraoperative acetaminophen dosage	0.0895	0.0835
Intraoperative lidocaine dosage	-0.0464	0.1431
Intraoperative ketamine dosage	-0.5140	0.2153
Intraoperative dexmedetomidine dosage	0.0583	0.0827
Intraoperative esmolol dosage	-4.3865	2.6538
Intraoperative dexamethasone dosage	-0.3993	0.1408
Difficult intubation	1.1580	1.1218
Conversion	-16.2306	7,581.7123
Postoperative ketorolac dosage	-0.3077	0.1786
Postoperative acetaminophen dosage	0.0179	0.0856
Postoperative gabapentin dosage	-0.0692	0.0965
Postoperative care location: PACU	1.0562	0.3547
Postoperative care location: CPC	1.0267	0.3327
Naloxone usage	1.1487	0.3476
Total opioid administration between time of extubation and PACU admission	-10.5773	416.6411
Total opioid administration between PACU admission and hospital discharge	0.0002	0.0000

© 2023 Santa Cruz Mercado LA et al. *JAMA Surgery.*

W. 30-day postoperative readmission

Coefficient	Value	Std. Error
(Intercept)	-3.7594	0.0446
Intraoperative fentanyl exposure	-0.0930	0.0165
Intraoperative hydromorphone exposure	-0.0112	0.0143
Intraoperative non-analgesic opioid usage	0.3202	0.1747
PACU non-analgesic opioid usage	0.2894	0.0596
Intraoperative Vasopressors	0.0521	0.0149
Intraoperative CYP3A4 Inducers	-0.1074	0.0505
Intraoperative CYP3A4 Inhibitors	0.8453	0.1437
Intraoperative Antihypertensive Drugs	-0.1990	0.0571
Intraoperative hypotensive duration	0.0159	0.0251
Intraoperative hypertensive duration	0.0432	0.0184
Intraoperative tachycardic duration	0.0617	0.0168
Intraoperative bradycardic duration	-0.1211	0.0271
N2O dosage	0.0437	0.0176
Desflurane dosage	-0.0466	0.0275
Isoflurane dosage	-0.0139	0.0206
Propofol dosage	-1.0678	0.1362
Sevoflurane dosage	0.0332	0.0230
Neuromuscular blocking agent usage	-0.0948	0.0438
Intraoperative mean blood pressure	0.1170	0.0190
Intraoperative mean heart rate	0.0040	0.0275
Surgical duration	-0.0618	0.0287
Intraoperative blood loss	0.0573	0.0130
Intraoperative blood transfusions	0.8479	0.0717
Surgery type (Ambulatory/Inpatient)	0.9145	0.0571
Intraoperative antiemetic prophylaxis	0.1661	0.0400
Postoperative nausea/vomiting in PACU	0.1581	0.1018
Intraoperative ketorolac dosage	-0.2112	0.0229
Intraoperative acetaminophen dosage	0.0496	0.0167
Intraoperative lidocaine dosage	-0.0474	0.0302
Intraoperative ketamine dosage	0.0215	0.0143
Intraoperative dexmedetomidine dosage	-0.0313	0.0219
Intraoperative esmolol dosage	-0.0101	0.0229
Intraoperative dexamethasone dosage	-0.1996	0.0246
Difficult intubation	0.4964	0.2143
Conversion	0.3021	0.3657
Postoperative ketorolac dosage	0.0043	0.0169
Postoperative acetaminophen dosage	-0.0690	0.0183
Postoperative gabapentin dosage	0.0134	0.0142
Postoperative care location: PACU	0.4806	0.0582
Postoperative care location: CPC	0.6971	0.0491
Naloxone usage	0.0538	0.1275
Total opioid administration between time of extubation and PACU admission	-0.0482	0.0182
Total opioid administration between PACU admission and hospital discharge	0.0002	0.0000

© 2023 Santa Cruz Mercado LA et al. *JAMA Surgery.*

eTable 2: Most frequent CPT codes in study cohort

CPT	Description	n	Frequency (%)
47562	PR LAP,CHOLECYSTECTOMY	2,318	3.78
29881	PR KNEE SCOPE,MED OR LAT MENISECTOMY	1,910	3.12
58571	PR LAPAROSCOPY W TOT HYSTERECTUTERUS <=250 GRAM W TUBE/OVARY	1,591	2.60
52356	PR CYSTO/URETERO W/LITHOTRIPSY &INDWELL STENT INSRT	1,540	2.51
58661	PR LAP,RMV ADNEXAL STRUCTURE	1,116	1.82
44970	PR LAP,APPENDECTOMY	985	1.61
49505	PR REPAIR ING HERNIA,5+Y/O,REDUCIBL	966	1.58
27130	PR TOTAL HIP ARTHROPLASTY	900	1.47
19301	PR MASTECTOMY, PARTIAL	897	1.46
55866	PR LAP,PROSTATECTOMY,RADICAL,W/NERVE SPARE,INCL ROBOTIC	829	1.35
43775	PR LAP, GAST RESTRICT PROC, LONGITUDINAL GASTRECTOMY	827	1.35
52648	PR LASER VAPORIZATION SURGERY PROSTATE, COMPLETE	796	1.30
49320	PR LAP,DIAGNOSTIC ABDOMEN	775	1.27
29880	PR KNEE SCOPE,MED & LAT MENISECTOMY	751	1.23
52000	PR CYSTOURETHROSCOPY	725	1.18
29877	PR KNEE SCOPE,SHAVE ARTICULAR CART	675	1.10
20680	PR REMOVAL DEEP IMPLANT	665	1.09
45990	PR SURG DIAGNOSTIC EXAM, ANORECTAL	585	0.96
55840	PR REMV PROSTATE,RETROPUB,RADICAL	584	0.95
63047	PR LAMINEC/FACETECT/FORAMIN,LUMBAR 1 SEG	583	0.95
58558	PR HYSTEROSCOPY,W/ENDO BX	570	0.93
44207	PR LAP,SURG,COLECTOMY,W/ANAST	566	0.92
19318	PR BREAST REDUCTION	553	0.90
19120	PR EXCISE BREAST CYST	535	0.87
58662	PR LAP,FULGURATE/EXCISE LESIONS	523	0.85

© 2023 Santa Cruz Mercado LA et al. *JAMA Surgery*.

eFigure 1: Forest plot of exposure variable coefficients for propensity-weighted models of primary and secondary outcomes without adjustment for comorbidities and surgical complexity as represented by CPT and ICD-10 codes.

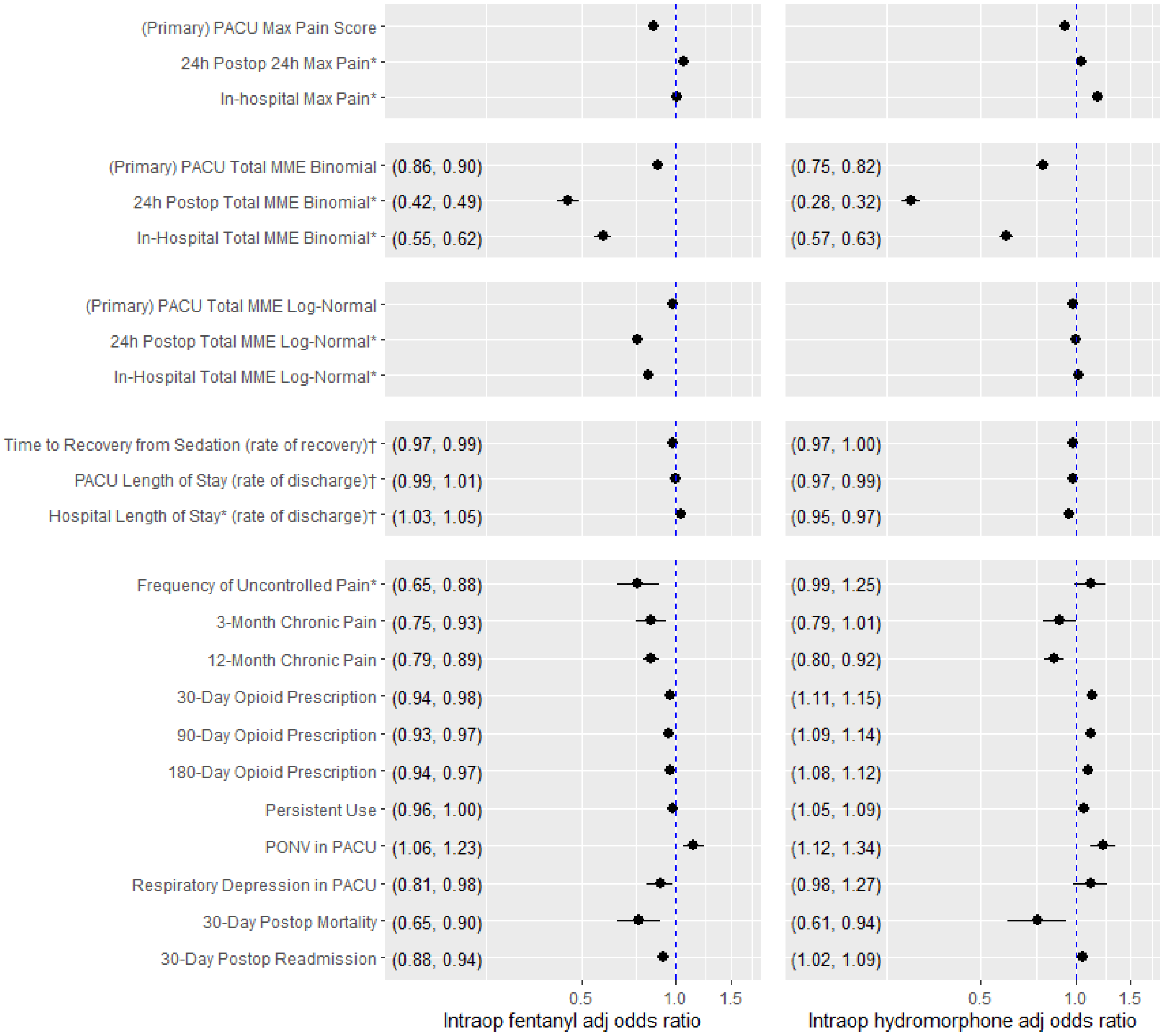

© 2023 Santa Cruz Mercado LA et al. *JAMA Surgery*.

eFigure 2: Forest plot of exposure variable coefficients with interaction term for propensity-weighted models of primary and secondary outcomes without adjustment for comorbidities and surgical complexity as represented by CPT and ICD-10 codes.

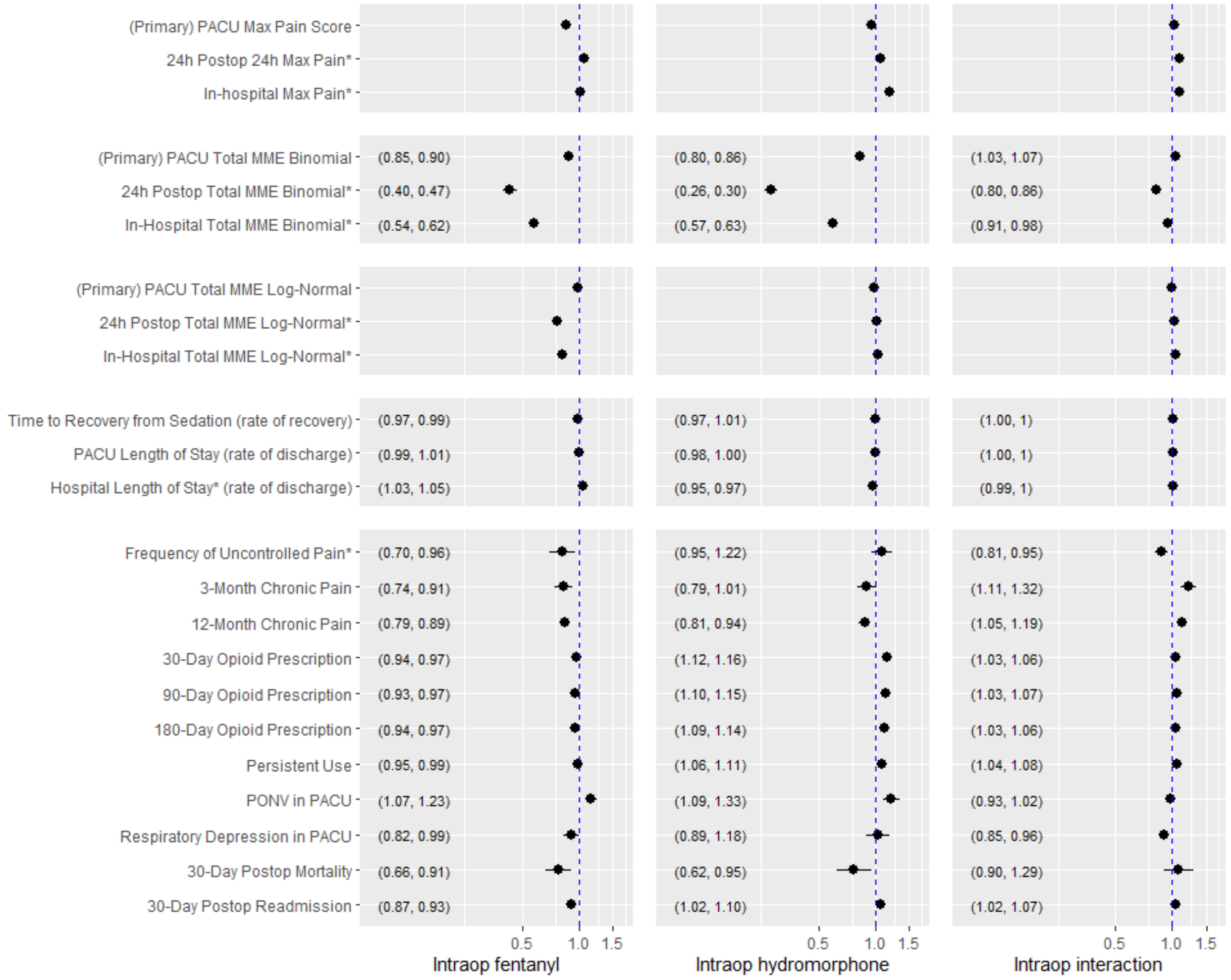

© 2023 Santa Cruz Mercado LA et al. *JAMA Surgery*.

eFigure 3: Forest plot of exposure variable coefficients with interaction term for propensity-weighted models of primary and secondary outcomes with adjustment for comorbidities and surgical complexity as represented by CPT and ICD-10 codes.

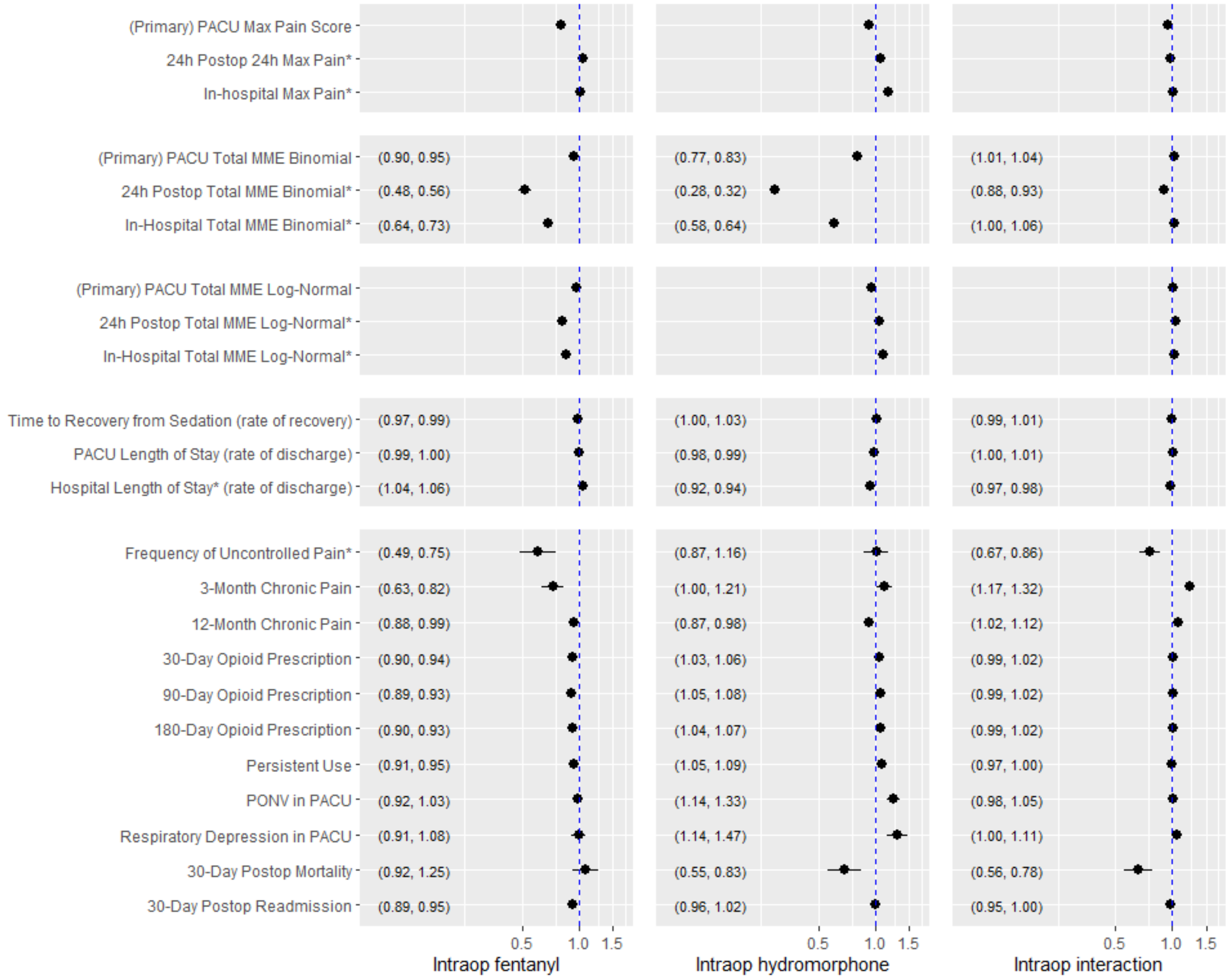

© 2023 Santa Cruz Mercado LA et al. *JAMA Surgery*.

eFigure 4: Subgroup analysis for patients with BMI >30 without adjustment for comorbidities and surgical complexity as represented by CPT and ICD-10 codes

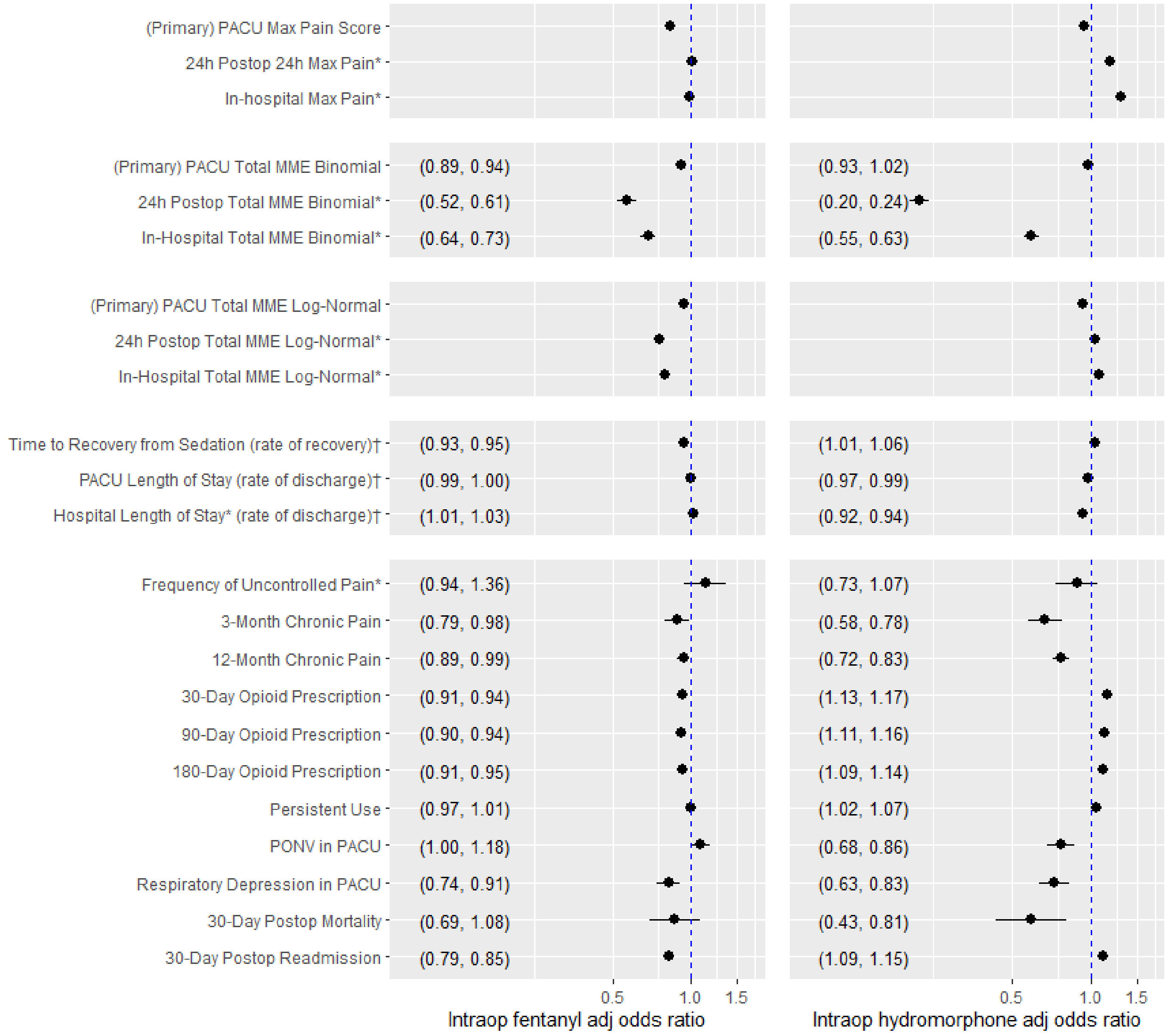

© 2023 Santa Cruz Mercado LA et al. *JAMA Surgery*.

eFigure 5: Subgroup analysis for patients with BMI >30 with adjustment for comorbidities and surgical complexity as represented by CPT and ICD-10 codes

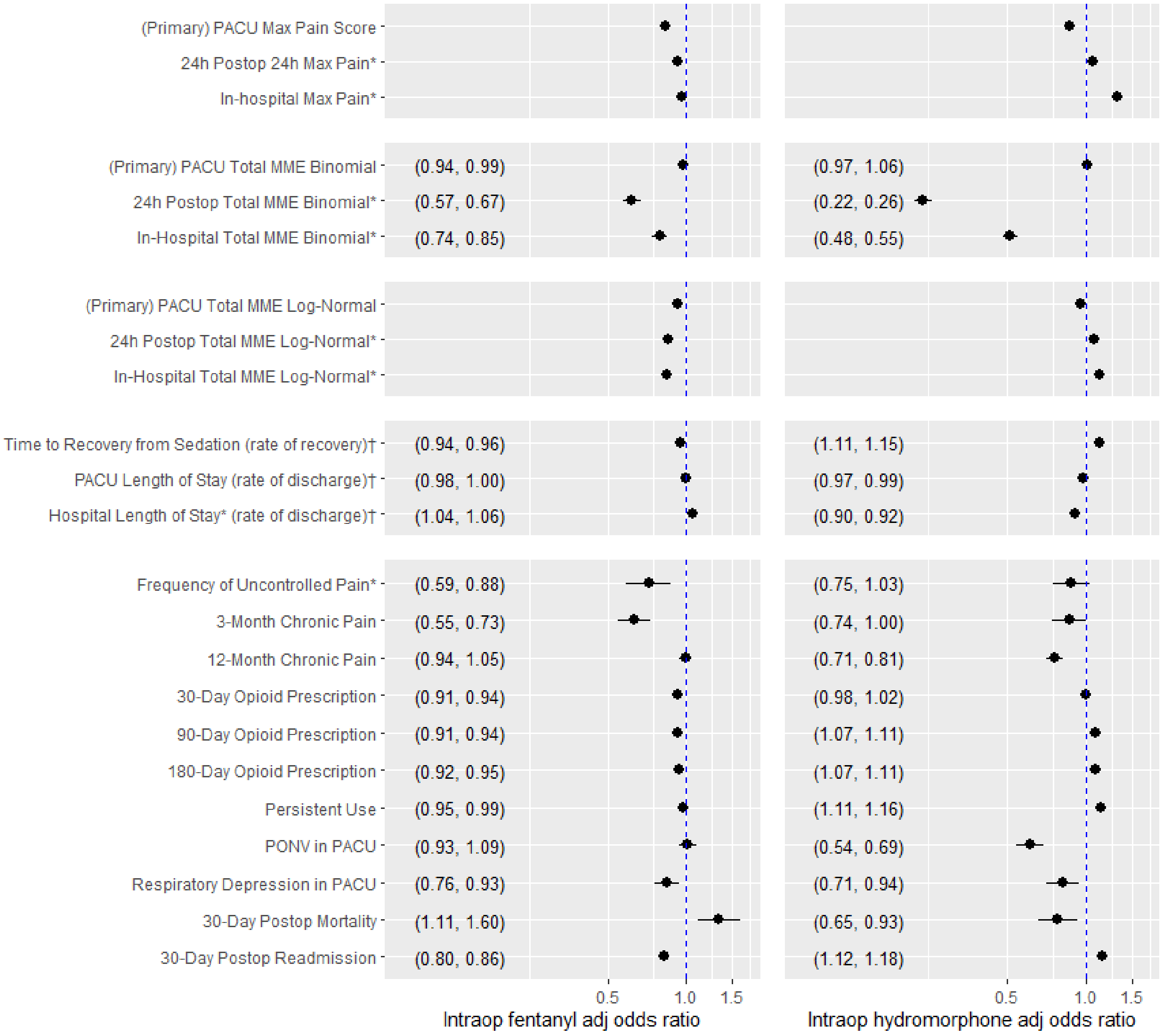

© 2023 Santa Cruz Mercado LA et al. *JAMA Surgery*.

eFigure 6: Distribution of timing of intraoperative opioid administration.

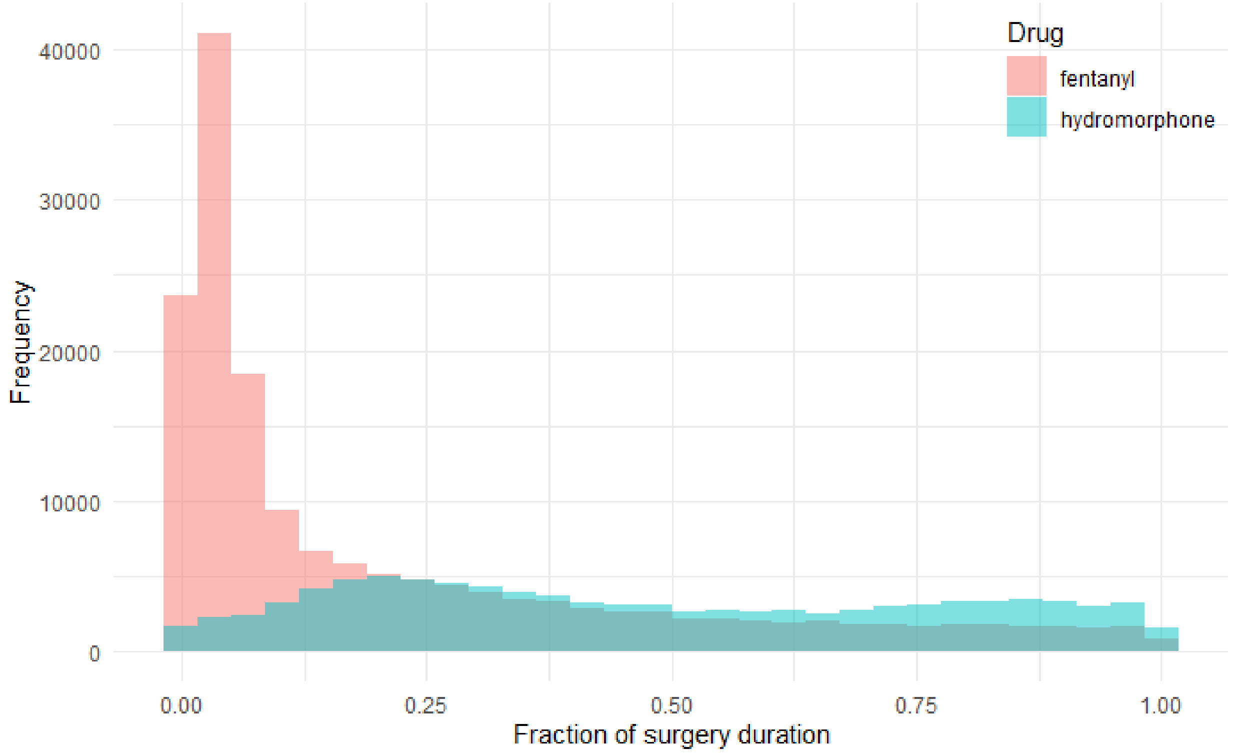

© 2023 Santa Cruz Mercado LA et al. *JAMA Surgery*.

eFigure 7: Distribution of secondary maximum pain score outcomes.

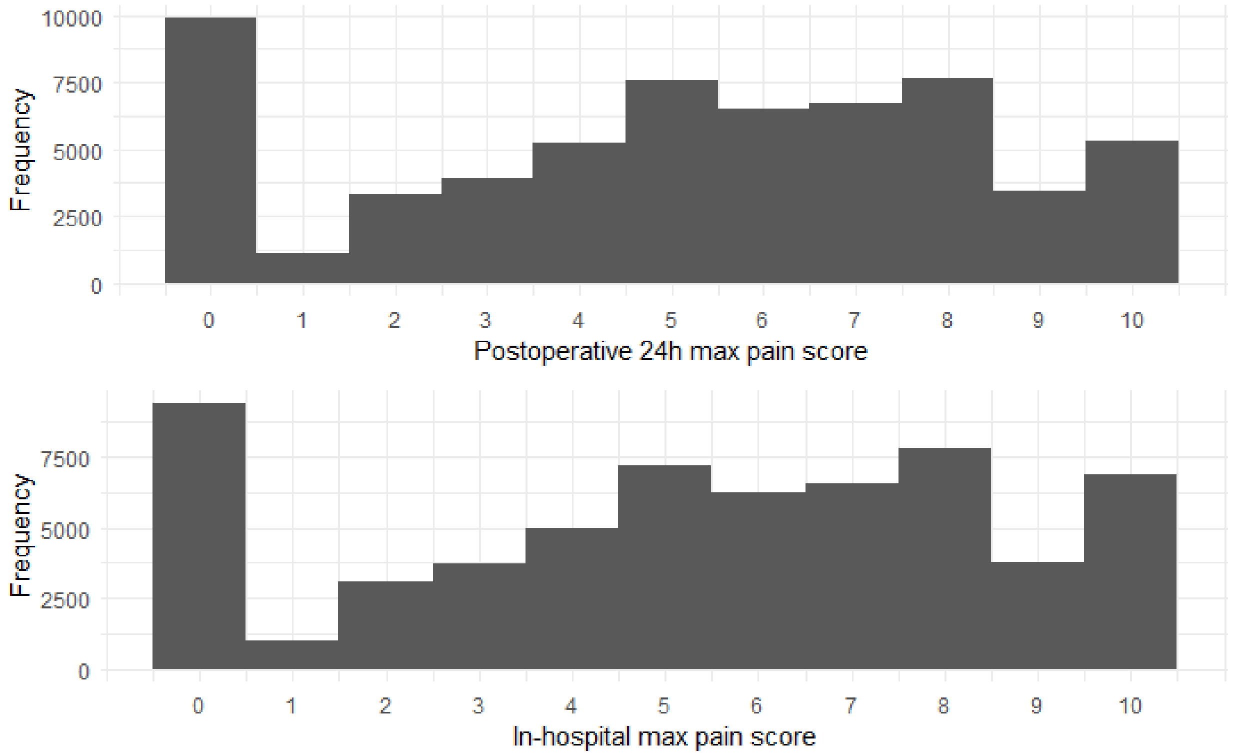

© 2023 Santa Cruz Mercado LA et al. *JAMA Surgery*.

eFigure 8: Number of pain assessments in each outcome window.
Mean values are indicated by the red dashed line. In PACU, the mean number of pain assessments is 4.75. In the 24 postoperative hours, the mean number of pain assessments is 14.93. The mean number of pain assessments in-hospital is 38.65. For the 24h postop and in-hospital windows, only inpatient surgeries are considered.

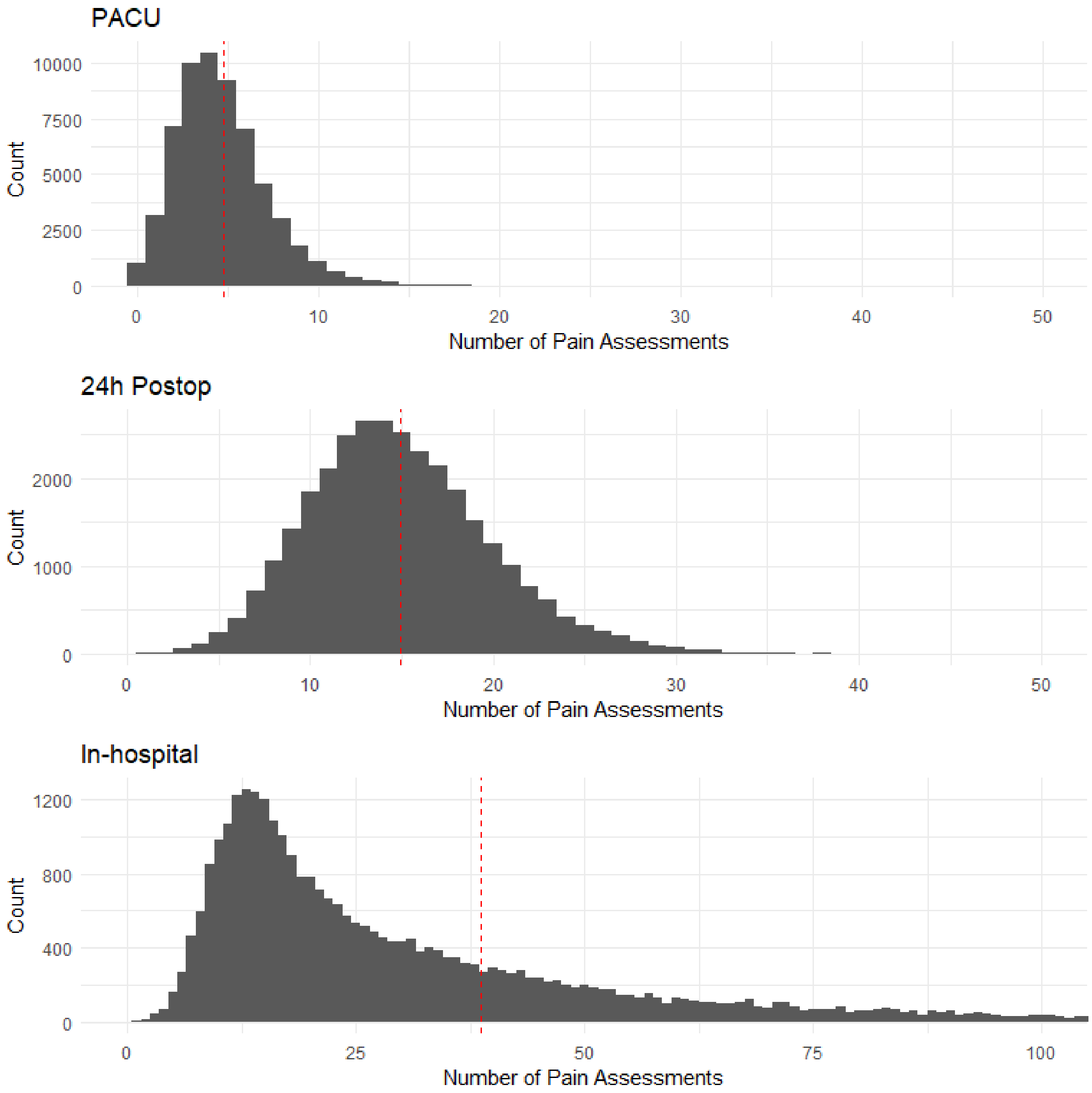

© 2023 Santa Cruz Mercado LA et al. *JAMA Surgery*.

eFigure 9: Smoothed plot of intraoperative exposure variables over time.

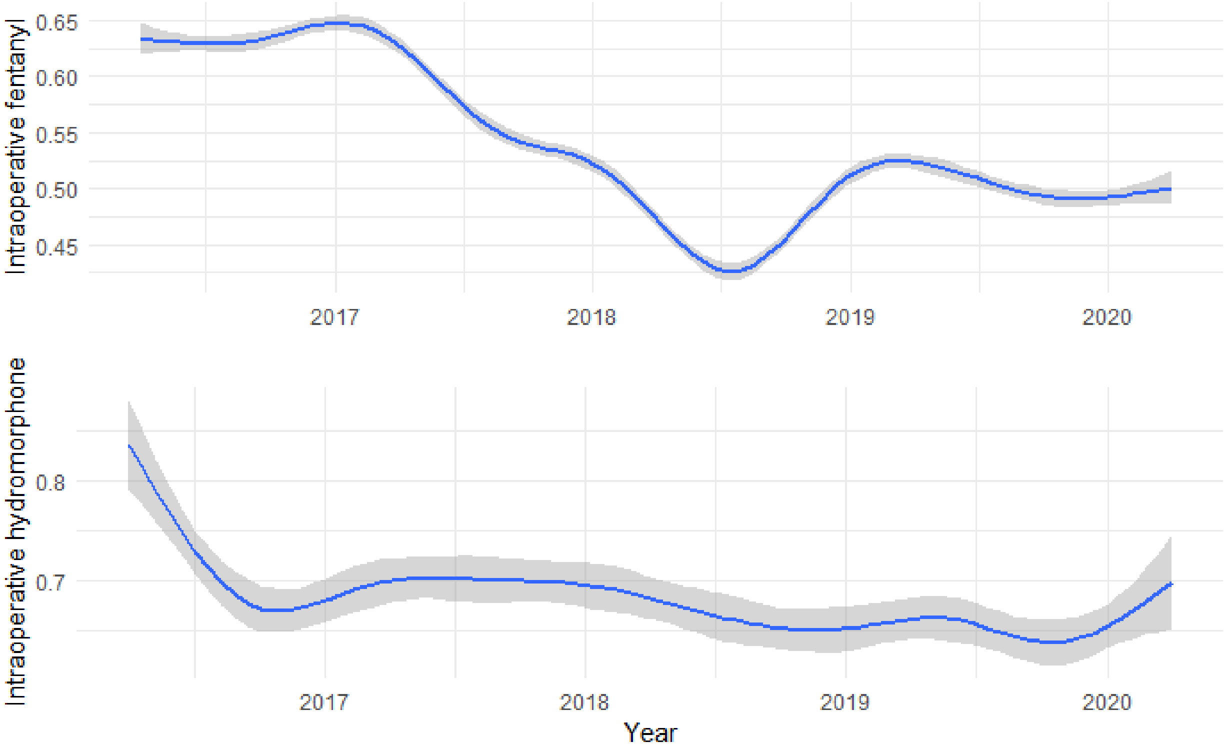

© 2023 Santa Cruz Mercado LA et al. *JAMA Surgery.*

eFigure 10: Distributions of total intraoperative opioid exposure, computed from time of patient arrival in the operating room to the end of the exposure window.

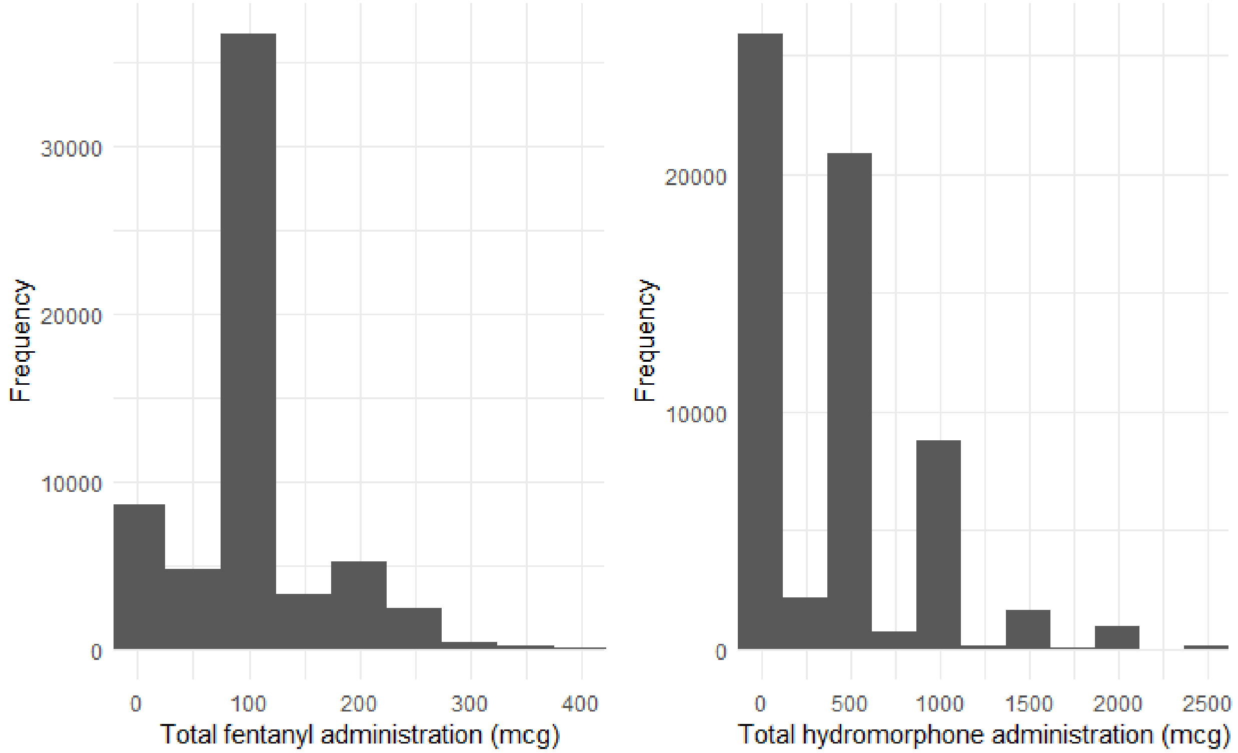

© 2023 Santa Cruz Mercado LA et al. *JAMA Surgery*.
