# Supplementary material for: Association of Intraoperative Opioid Administration With Postoperative Pain and Opioid Use
Source: JAMA Surg. 2023 Jun 14;158(8):854–64. doi: 10.1001/jamasurg.2023.2009 (PMC10267849; doi:10.1001/jamasurg.2023.2009)
Supplement: Supplement 2. — Data sharing statement [file jamasurg-e232009-s002.pdf]

Data Sharing Statement

Santa Cruz Mercado. Association of Intraoperative Opioid Administration With Postoperative Pain and Opioid Use. *JAMA Surg*. Published June 14, 2023. doi:10.1001/jamasurg.2023.2009

Data
Data available: No

Additional Information
Explanation for why data not available: Upon request to Dr. Patrick Purdon with a scientific question de-identified data can be shared.
